# Inflammatory markers after supplementation with marine n-3 or plant n-6 PUFAs: A randomized double-blind crossover study

**DOI:** 10.1016/j.jlr.2025.100770

**Published:** 2025-03-08

**Authors:** Elise Grytten, Johnny Laupsa-Borge, Kaya Cetin, Pavol Bohov, Jan Erik Nordrehaug, Jon Skorve, Rolf K. Berge, Elin Strand, Bodil Bjørndal, Ottar K. Nygård, Espen Rostrup, Gunnar Mellgren, Simon N. Dankel

**Affiliations:** 1 Hormone Laboratory, Department of Medical Biochemistry and Pharmacology, Haukeland University Hospital, Bergen, Norway; 2 Mohn Nutrition Research Laboratory, Department of Clinical Science, University of Bergen, Bergen, Norway; 3 Bevital AS, Bergen, Norway; 4 Department of Clinical Science, University of Bergen, Bergen, Norway; 5 Department of Heart Disease, Haukeland University Hospital, Bergen, Norway

**Keywords:** PUFAs, omega-3, omega-6, inflammation, endothelial function, blood pressure, adipose tissue, crossover study, abdominal obesity

## Abstract

Omega-3 (n-3) (e.g., EPA/DHA) and omega-6 (n-6) (e.g., linoleic acid [LA]) FAs are suggested to have opposite effects on inflammation, but results are inconsistent and direct comparisons of n-3 and n-6 are lacking. In a double-blind, randomized, and crossover study, females (n = 16) and males (n = 23) aged 30–70 years with abdominal obesity were supplemented with 3–4 g/d EPA/DHA (fish oil) or 15–20 g/d LA (safflower oil) for 7 weeks, with a 9-week washout phase. Cytokines and chemokines (multiplex assay), acute-phase proteins (MALDI-TOF mass spectrometry), endothelial function (vascular reaction index), blood pressure, FA composition (red blood cell membranes/serum/adipose tissue, GC-MS/MS), and adipose gene expression (microarrays, quantitative PCR) were measured. While significant differences between treatments in relative change scores were found for systolic blood pressure (n-3 vs. n-6: −1.81% vs. 2.61%, *P* = 0.003), no differences between n-3 and n-6 were found for any circulatory inflammatory markers. However, compared with baseline, n-3 was followed by reductions in circulating TNF (−24.9%, *P* < 0.001), regulated upon activation, normal T cell expressed and secreted (−12.1%, *P* < 0.001), and macrophage inflammatory protein 1-beta (−12.5%, *P* = 0.014), and n-6 by lowered TNF (−18.8%, *P* < 0.001), regulated upon activation, normal T cell expressed and secreted (−7.37%, *P* = 0.027), monocyte chemoattractant protein-1 (−7.81%, *P* = 0.020), and macrophage inflammatory protein 1-beta (−14.2%, *P* = 0.010). Adipose tissue showed significant treatment differences in weight percent of EPA (n-3 vs. n-6: 50.2%∗ vs. −1.38%, *P* < 0.001, ∗: significant within-treatment change score), DHA (16.0%∗ vs. −3.67%, *P* < 0.001), and LA (−0.033 vs. 4.91%∗, *P* < 0.001). Adipose transcriptomics revealed overall downregulation of genes related to inflammatory processes after n-3 and upregulation after n-6, partly correlating with changes in circulatory markers. These data point to tissue-specific proinflammatory effects of high n-6 intake, but a net systemic anti-inflammatory effect as for n-3.

Inflammatory factors play an important role in the pathogenesis of atherosclerosis, the underlying cause of most CVD (1). Proinflammatory cytokines can cause endothelial dysfunction (2) and induce expression of cell adhesion molecules, which in turn mediate a series of inflammatory reactions that promote plaque formation (3). Elevation of inflammatory markers has been reported in several CVD studies (4, 5), and the clinical relevance of the proatherosclerotic effect was indicated by the CANTOS trial, where anti-inflammatory therapy targeting interleukin-1β (IL-1β) reduced inflammation and cardiovascular events in high-risk patients (6).

Numerous inflammatory markers have been linked to CVD. The acute-phase protein C-reactive protein (CRP), produced by the liver in response to interleukin-6 (IL-6), is considered a strong predictor of CVD risk (7). Other acute-phase reactants, including serum amyloid A (SAA) and calprotectin (also known as the S100A8/9 [S100 calcium-binding protein A8/A9] complex) are also associated with CVD (8, 9, 10), in addition to cytokines and chemokines, such as TNF, monocyte chemoattractant protein-1 (MCP-1), macrophage inflammatory protein 1-alpha (MIP-1α), and beta (MIP-1β), regulated upon activation, normal T cell expressed and secreted (RANTES), and interleukin-1 receptor antagonist (IL-1RA) (11, 12, 13). In contrast, the adipokine adiponectin has anti-inflammatory properties and is inversely related to CVD (14).

Proinflammatory mediators are often higher in individuals with obesity than in normal-weight people (15). White adipose tissue (AT) is an endocrine organ secreting a wide range of adipokines and chemokines that regulate inflammation both locally and systemically (16). Excessive body fat, especially visceral adiposity, is associated with dysfunctional AT and a chronic low-grade inflammatory state. Chronic inflammation underlies several metabolic diseases, including CVD, and approaches to reduce inflammation in obesity may have great clinical value (17).

Dietary patterns are important factors affecting AT function and whole-body physiology, including inflammation (18). PUFAs, which include omega-3 (n-3) and omega-6 (n-6), are recommended as a replacement of saturated FAs to reduce risk of CVD (19, 20), in part by lowering of LDL-C (21, 22) and triacylglycerols (TAGs) (23, 24). It is also commonly thought that n-3 PUFAs, especially the marine-derived EPA and to a lesser extent DHA, are anti-inflammatory (25, 26) including in patients with diabetes and CVD (27). n-3 PUFAs are also used to treat inflammatory diseases, such as rheumatoid arthritis (28) and inflammatory bowel disease (29). However, results from intervention studies in healthy people with overweight and obesity or other metabolic alterations (e.g., hypertriglyceridemia) have been inconsistent (30, 31, 32), and recent studies investigating cardiovascular benefits have also shown conflicting results (33, 34, 35).

Linoleic acid (LA), the most common n-6 PUFA in the diet, has on the other hand been assumed to promote inflammation as it is converted into arachidonic acid (ARA), which can be used for production of proinflammatory eicosanoids (36, 37). Also, there are concerns regarding LA serving as a precursor of oxidized LA metabolites (38) and the potential of LA to lower the conversion of α-linolenic acid to longer-chain n-3 PUFAs by competing for the enzymes in the same metabolic pathways (39). There is however controversy over its potential inflammatory properties (40). While several in vitro and in vivo studies have found proinflammatory actions of LA (41, 42, 43), randomized controlled trials (RCTs) in humans have failed to show any relationship between increased n-6 intake and elevations in inflammatory markers (44). Epidemiological studies have rather shown an inverse association between circulating levels of n-6 and inflammatory markers (45, 46), which, at least in part, might be explained by the discovery that LA and ARA also are involved in anti-inflammatory signaling pathways (40). The picture is further complicated as studies investigating LA might be confounded by a concomitant increase in n-3 PUFAs as oils rich in LA also often contain α-linolenic acid. Moreover, previous research has demonstrated limited enzymatic conversion of LA to ARA, estimated to be 0.3–0.6% in stable isotope tracer studies (47), which is in line with our previous findings (48). Thus, the effects of PUFA intake on inflammation need clarification, especially for n-6.

In previous studies evaluating the effect of n-3 PUFAs on circulating inflammatory markers, much focus has been on classic proinflammatory cytokines, including TNF, IL-1β, and IL-6 (49), and fewer studies have analyzed a broad range of inflammatory parameters, which might better reflect the overall inflammatory status. In many RCTs of n-6 PUFAs using oils high in LA and low in n-3 PUFA, such as safflower oil and sunflower oil, CRP was the single marker of inflammation reported (50, 51, 52, 53, 54), and only some studies measured a few additional markers, such as IL-6, IL-10, and TNF (55, 56, 57, 58, 59, 60, 61, 62, 63). Moreover, many n-3 PUFA intervention trials have used a relatively low dose of EPA and/or DHA, and few studies have been performed with doses above 3 g/day in individuals with overweight/obesity (64, 65). Also, studies have typically not provided detailed characterization of the intervention oils, including FA composition, oxidation status, and content of trans FAs and FFAs, which may influence inflammatory potential and/or other biological responses (66).

Addressing secondary outcomes of a double-blind crossover trial on high-dose supplementation with n-3 or n-6 PUFAs from high-quality oils (48, 67), the aim of the present study was to determine possible changes in circulatory and adipose inflammatory status, along with assessment of other key CVD risk factors (endothelial function and blood pressure). To gain further insight into biological mechanisms in AT, we also investigated effects of PUFA treatment on inflammatory markers in primary human adipocytes in vitro.

## Materials and Methods

### Subjects and ethics

Healthy males and females aged 30–70 years were recruited among respondents to advertisements in local newspapers in Bergen, Norway. Inclusion criteria were increased waistline (≥94 cm in males and ≥80 cm in females) and an overall sedentary lifestyle (<2 h vigorous/active exercise training each week). Subjects were excluded if using medications that could influence the study outcomes (e.g., lipid-lowering drugs) or if they were diagnosed with diabetes type 1 or 2, severe psychiatric illness, or malabsorption disorders. Other criteria for exclusion were fasting serum levels of TAGs >5 mmol/l, cigarette smoking, alcohol or drug abuse, previous bariatric surgery, pregnancy or lactation, previous coronary intervention, pacemaker, or implantable cardioverter defibrillator.

The study was conducted in accordance with the Declaration of Helsinki guidelines and was approved by the Regional Committee for Medical and Health Research Ethics (2014/2336/REK Sør-Øst). Before inclusion in the study, written informed consent was obtained from all study participants. The study was registered at clinicaltrials.gov (NCT02647333).

### Study design

This randomized, double-blind, and single-center crossover trial has been described previously (48, 67). There were two 7-week intervention periods separated by a 9-week washout phase. Following a 15-week run-in period, participants were randomized to treatment sequence AB (n-3 fish oil in the first period, n-6 safflower oil in the second period) or BA (n-6 in the first period, n-3 in the second period). The n-3 supplement was a hydrolyzed and re-esterified TAG fish oil, containing 38.6 weight percent (wt%) EPA, 23.5 wt% DHA, and 1.1 wt% LA, of which females received 3 g (1.8 g EPA and 1.2 g DHA) and males 4 g (2.4 g EPA and 1.6 g DHA) each day (5.6 ml and 7.5 ml oil/day for females and males, respectively). The n-6 supplement was an organic, cold-pressed safflower oil, containing 66.7 wt% LA and 0.29 wt% EPA/DHA, of which females received 15 g and males 20 g LA each day (24.5 ml and 32.7 ml oil/day for females and males, respectively). The doses were chosen based on the expected efficacy of the respective PUFAs and to provide similar doses per kg body weight for males and females. The daily dosage was divided in two; one in the morning and one in the afternoon with meals. Both oils were given in liquid form and were similarly flavored with citrus and stevia to enable the double-blind design. Emphasis was made on high-quality, low FFA, and oxidized FA content, where the total oxidation values (ToTox) were 3.3 and 10.9 for the fish and safflower oils, respectively, and the levels of FFAs were 0.09% and 0.17% at delivery. The oils were produced by Pharmatech AS (Rolvsøy, Norway), and oxidation products, FFAs, and acid values were measured by Multilab Østfold AS (Rolvsøy, Norway).

The study was carried out from May 2015 to March 2016. During the study, participants were instructed to maintain their habitual diet and their usual activity status. Habitual dietary intake was assessed by 7-day dietary records twice during the run-in period (baseline) and during week 5 in both intervention periods. The recordings were performed electronically by using a self-developed application designed using the database program FileMaker Pro 12 Advanced (FileMaker, Inc, Santa Clara, CA). Subjects met for study visits at baseline (two visits; baseline visit 1.1 and 1.2), during week 7 and 8 in the first intervention period (two visits; follow-up visit 1.1 and 1.2), at the second baseline visit after the washout period (one visit; baseline visit 2), and in week 7 and 8 of the second intervention period (one visit; follow-up visit 2). Participants attended two visits for baseline and follow-up measurements in period 1 due to collection of AT biopsy only in this period (baseline visit 1.2 and follow-up visit 1.2). At all the other visits, anthropometrics, bioelectrical impedance analysis, blood sampling, blood pressure measurement, and endothelial function test were performed. Prior to the visits, the participants were asked to abstain from alcohol consumption for 48 h, to fast for 10–12 h, and to avoid exercise the day before. Compliance to the intervention was determined by measuring the leftover oil, asking about supplement intake at the visits, and by analyzing the FA composition in red blood cell membranes (RBCMs).

### Laboratory analysis

Venous blood samples from subjects were collected into tubes containing disodium-EDTA or gel after an overnight fast. Tubes containing disodium-EDTA were immediately centrifuged at 1,800 g for 10 min for collecting plasma, whereas tubes containing gel were centrifuged after a minimum of 30 min and a maximum of 2 h at 1,800 g for 10 min for recovering serum. All samples were stored at −80°C until further analyses (performed in 2019, except analyses of FAs in AT performed in 2023). Plasma adiponectin was measured using Human ADIPOQ/Adiponectin ELISA kit (Sigma-Aldrich), and serum apelin was measured by Apelin EIA kit (Sigma-Aldrich) according to the manufacturer’s instructions. Serum levels of cytokines and chemokines, including eotaxin, MCP-1, MIP-1α, MIP-1β, RANTES, TNF-α, IL-1RA, and interleukin-8 (IL-8), were measured simultaneously by using Bio-Plex Pro Human Cytokine Assay (Bio-Rad) according to the manufacturer’s procedures. Plasma concentrations of SAA (SAAt [SAA total], SAA1t, and serum amyloid A 2 total [SAA2t]) and calprotectin (S100t, S100A8, and S100A9), as well as high-sensitive C-reactive protein were quantified by MALDI-TOF mass spectrometry at Bevital AS, Norway (www.bevital.no). The FA composition of RBCMs (isolated from frozen whole blood) was assessed by gas LC-MS/MS as previously described (48).

### Blood pressure

Blood pressure was measured by a BP-200 plus blood oscillometric monitor (Schiller AG, Baar, Switzerland). After 15 min supine rest, three measurements were performed at 1-min intervals. All measurements were recorded, and the mean of the last two measurements was calculated.

### Endothelial function

Vascular function was measured using a digital thermal monitoring system (VENDYS; Endothelix Inc, Houston, TX) in a lying posture after 30 min of rest in a quiet, dimmed, and temperature-controlled room, according to the manufacturer’s protocol. The test started with a measurement of the blood pressure in the left arm, followed by 5 min of stabilization, 5 min of cuff occlusion to 50 mm Hg greater than systolic blood pressure, and 5 min of deflation causing reactive hyperemia (in the right arm). Thermal changes in the fingertip of both the occluded and nonoccluded arm were monitored before, during, and after the arm-occlusive reactive hyperemia test. Post-cuff-deflation temperature rebound and area under the temperature curve were measured automatically by using VENDYS software. The results are presented as a vascular reactivity index (VRI) that indicate poor (<1), intermediate (1-2), or good (>2) vascular reactivity.

### Analyses of AT

#### AT biopsy collection

Biopsies from subcutaneous adipose tissue (SAT) were obtained from all but one participant under local anesthesia (10–15 ml xylocaine) and sterile conditions after an overnight fast before and after the intervention but for ethical reasons only in the first 7-week period of the study (before the crossover). One female was unwilling to donate a biopsy at the follow-up visit due to discomfort and possible scarring after the first biopsy. A 2 mm skin incision was made 5–10 cm lateral to the umbilicus, and a Hepafix Syringe was used to create vacuum and aspirate the tissue. Biopsies collected at the follow-up visit were obtained from the opposite side of the umbilicus than at the baseline visit. Each biopsy was immediately washed with 0.9% sodium chloride, gently pressed flat in a sealable polyethylene bag, and snap-frozen in liquid nitrogen before storing at −80°C until analysis.

#### FA analysis

About 30 mg of SAT was homogenized in 6 ml of ice-cold mixture of chloroform-methanol (2:1). The homogenate was then shaken for 2 min every 15 min for 1 h, and lipids were extracted overnight at −20°C. The aliquot of extraction mixture corresponding to about 110 μg of tissue was transferred into a derivatization vial, and solvents were evaporated under stream nitrogen. FA methyl esters were prepared and analyzed by gas-LC using the internal standard C21:0 as previously described (68).

#### RNA isolation

Approximately 200 mg of frozen SAT was sliced on dry ice and transferred to a microcentrifuge tube. RNA was isolated by the RNA/DNA/Protein Purification Plus Kit (Norgen) following the manufacturer’s protocol. The RNA yield was quantified by a NanoDrop® ND-1000 spectrophotometer (NanoDrop Technologies), and RNA integrity was assured with an Agilent 2100 Bioanalyzer (Agilent Technologies).

#### Microarray gene expression analysis in AT

For the microarray analysis, AT biopsies from 35 of the participants before and after the first intervention period were analyzed. One male in each sequence was randomly removed because of space limitation on the array. Reverse transcription and amplification of biotin-labeled antisense mRNA was performed with 500 ng of total RNA using the Illumina TotalPrep RNA Amplification Kit (Applied Biosystems/Ambion). Seven hundred fifty nanograms of the biotinylated RNA (complementary RNA) were further fragmentated and hybridized onto the HumanRef-8 v.3 Illumina Sentrix BeadChip, covering about 24,500 probes or 18,600 unique genes, and analyzed by the Illumina iScan system.

Rank Product Analysis (RPA), a nonparametric algorithm that assigns a rank to each microarray probe (69), was used to identify differentially expressed genes, based on the product and fold change of each probe for each intervention and its *P* value (100 permutations). Probe intensities were log_2_ transformed and Robust Multichip Average normalized, and RPA was then implemented via the R package RankProd v3.1.0. RPA results were submitted to gene set enrichment analysis (GSEA) via the R package fgsea v1.16.0. Genes were ranked by fold change and submitted for enrichment analysis against Gene Ontology sets provided by MSigDB as well as against the MSigDB hallmark gene sets (70). Ontology and gene expression results were visualized via fgsea and the R package ggplot2 v3.3.3.

#### Validation of microarray data by quantitative PCR

Expression of a selection of genes was validated by quantitative PCR (qPCR). Four hundred nanograms of RNA were reverse transcribed to single-stranded complementary DNA (cDNA) using High-Capacity cDNA Reverse Transcription Kit (Applied Biosystems), according to the manufacturer’s procedure, and the cDNA was diluted to 1:10 by PCR-grade water. qPCR was performed using SYBR Green on the LightCycler® 480 DNA system (Roche). Primers were designed using Primer-BLAST software (sequences shown in supplemental Table S1). Relative gene expression was calculated using the delta-delta CT method. Due to stable gene expression in AT, *HPRT* mRNA was used as a reference to normalize expression levels between samples. All samples were analyzed in triplicate.

### Cell culture experiments

#### Isolation of stromal-vascular fraction

For in vitro experiments, SAT was obtained from patients undergoing abdominal liposuction at Aleris Hospital in Bergen. Stromal-vascular fraction was isolated as previously described (71). Briefly, liposuction aspirate was digested by incubating with equal amounts of KRP buffer (Krebs-Ringer Phosphate buffer; 0.1% BSA and ∼55 Wunch/liter collagenase with thermolysin [Liberase Blendzyme Thermolysin Medium 10X; Roche]) on a shaker for about 1 h at 37°C. The dissolved tissue was then filtered through a 210 μm nylon mesh into a vacuum filtration funnel. After 10 min of gravimetric separation, the stromal-vascular fraction in the bottom phase was drained and centrifugated at 300 g for 5 min at room temperature. After the supernatant was carefully discarded, erythrocytes were removed by hemolysis through incubation of the cell pellet with a buffer containing 155 mM ammonium chloride, 5.7 mM dipotassium phosphate, and 0.1 mM EDTA for 10 min. The solution was then centrifuged at 300 g for 5 min, before 10 ml preheated cell medium (37°C) was added to the cell pellet. Cells were finally filtered through a 70 μm nylon mesh cell strainer (BD Falcon), counted with a Bürker chamber, and either seeded out in 24-well plates (“freshly isolated” preadipocytes) (∼100,000 cells/well) or expanded and frozen in liquid nitrogen for later use (“frozen” preadipocytes).

#### Culturing, differentiation, and treatment of primary human adipocytes

Freshly isolated preadipocytes were grown in DMEM Glutamax medium supplemented with 10% FBS and 1% gentamycin. Differentiation medium containing insulin (66 nM), rosiglitazone (10 μM), pantothenate (17 μM), T3 (1 nM), Hepes buffer (15 mM), cortisol (100 nM), transferrin (10 μg/ml), and biotin (33 μM) were added after 24 h. Rosiglitazone was only included until day 6. “Frozen” preadipocyte cultures were seeded out in 24-well plates with DMEM Glutamax medium supplemented with 10% FBS and 1% GENT, epidermal growth factor (0.01 μg/ml), fibroblast growth factor (0.001 μg/ml), and insulin (8.7 μM). Differentiation was induced after 24 h by adding medium containing insulin (66 nM), rosiglitazone (10 μM), and IBMX (0.5 mM). After 5 days, pantothenate (17 μM), T3 (1 nM), Hepes buffer (15 mM), cortisol (100 nM), transferrin (10 μg/ml), and biotin (33 μM) were also added. As with fresh cells, rosiglitazone was not added after day 6.

Cells coming from both the fresh (n = 4) and frozen (n = 2) preadipocytes were considered mature adipocytes at day 12 and treated with EPA (*cis*-5,8,11,14,17-EPA; Sigma-Aldrich), DHA (*cis*-4,7,10,13,16,19-DHA; Sigma-Aldrich), or LA (9-*cis*,12-*cis*-LA; Sigma-Aldrich). Each FA was dissolved in ethanol to prepare a stock solution of 100 mM. The stock solutions were then diluted in warmed FBS (37°C) for complexing the FAs to albumin in the serum before addition to the differentiation medium. The final concentrations of FAs (200 μM LA or 100 μM EPA + 100 μM DHA) in the medium were based on dosages used in previous studies (72, 73). Controls received only ethanol and the differentiation medium containing 10% FBS. After incubation for 24 h, cells were lysed for RNA extraction and stored at −80°C until further analysis.

#### RNA isolation, cDNA synthesis, and qPCR

RNA from lysed cultured human adipocytes was extracted by using RNeasy mini kit (Qiagen) according to the manufacturer’s procedures, and yield and quality were measured with NanoDrop ND-1000 spectrophotometer (NanoDrop Technologies). cDNA synthesis and qPCR for selected genes were performed as described for RNA from AT biopsy samples.

### Statistics

The results reported in this article are from analyses of secondary outcomes and exploratory of nature. Outcome measurements are between-group differences in relative and absolute change scores derived from an intention-to-treat analysis including available data from all randomized participants (16 females and 23 males), and the sample size calculation for the primary outcomes has been previously reported (48).

Analyses of study outcomes were performed by baseline-adjusted constrained linear mixed-effect modeling (cLMM), a constrained longitudinal data analysis technique (74, 75, 76, 77), with “subject” as the random factor to account for repeated measurements. For the primary mixed model, we followed a design-driven approach and defined a fixed effects structure including “time,” “sex,” “period,” and “treatment × time,” chose a random effects structure with random intercepts and slopes for “time”, and used the correlation structure “compound symmetry,” which is appropriate for a crossover design (78). By excluding the main term “treatment” and thereby constraining the baseline values to be equal across treatment groups, a reasonable assumption in RCTs, the cLMM inherently adjusts for baseline differences (74, 76, 77), i.e., period-specific baselines (within-subject effect) in a crossover design. We also included subject-averaged baselines (between-subject effect) as a fixed covariable to control for cross-level bias in this design (78). The primary analysis was further adjusted for period (crossover design) and sex (stratified randomization). In the case of heterogeneity, the model included a variance function structure allowing for different variances per stratum of “treatment” and/or “time.” cLMM was performed by using the *lme* function in the *nlme* package v3.1-159. Values were transformed by natural logarithm before analyses of responses in relative terms.

To estimate both baseline-adjusted within-group changes and between-group differences in change scores, a computational trick was implemented in the cLMM. Specifically, the “treatment × time” interaction term was replaced with a time-dependent covariate representing the treatment effect. This new variable was added to the data in a long format and set equal to the “time” variable for one treatment group and to zero (i.e., baseline) for all time points in the other (reference) group. Since the cLMM assumes that the baseline means are equal across arms, the new time-varying covariable essentially estimates the mean difference between treatments at the follow-up time, which is analytically equivalent to an ANCOVA approach (76). However, in contrast to this approach, the cLMM also provides baseline-adjusted within-treatment change scores.

The Shapiro-Wilk test for normality, the D’Agostino test for skewness, and graphical tools, including boxplots, quantile-quantile plots, and histograms, were used to assess the distribution of standardized residuals as part of the model validation procedure and exploration of potential outliers. Cook’s distance and standardized DFBETAs were also used to identify influential outliers. All data are shown as raw unadjusted means (SDs), geometric means (1 SD ranges), or mean score differences (±absolute/relative effect estimates [95% CIs]). The geometric SD ranges used in descriptive statistics were calculated by dividing and multiplying the geometric means with the geometric SD factors to obtain the lower and upper limits, respectively (79, 80). Relative within-treatment changes from baseline to follow-up and between-treatment differences are calculated from the regression coefficient by using the formula 100 × (exp^estimate^ −1). Sympercent (s%) changes, calculated as the difference between the natural logarithm of two numbers multiplied by 100 (81), i.e.,100×ln(a) – 100×ln(b), are displayed in the figures, as previously explained (48). All inferential tests were two tailed with a nominal alpha level of 0.05.

To investigate correlations between variables, bivariate and partial Pearson’s correlation analyses were performed, and 95% CIs were obtained from a bootstrapping procedure (bias-corrected and accelerated, BCa) using 1,000 or 2,000 replicates.

Missing data handling, stratification analyses, and secondary analyses are described in the Supplemental Methods section.

The statistical analyses were performed with R, v4.1.2 (https://www.r-project.org), and data transformation and exploration were done by using the *tidyverse* packages (https://tidyverse.tidyverse.org). Correlation analyses were performed in SPSS Statistics, version 27 (SPSS, Inc, IBM Company) and in R with the *RVAideMemoire* package v0.9-81-2 using the *pcor.test* function. Analysis of significance of cell culture experiments was performed in GraphPad Prism v8.0.1 (GraphPad Software, San Diego, CA) using nonparametric ANOVA (Friedman’s test with Dunn’s multiple comparison test). All plots were made in GraphPad Prism.

## Results

The baseline characteristics of the participants, as well as dietary intake, lipid levels, and RBCM FA composition throughout the study, were previously reported (48). In summary, the participants were middle aged (56 [SD 9.3] years) and overweight (BMI, kg/m^2^: 28.5 [4.5] in females, 29.8 [3.8] in males), had abdominal obesity (waist circumference: 100 [11.2] cm in females, 107 [8.7] cm in males), and were normolipidemic (n = 7) or hyperlipidemic (n = 32). During the n-3 supplementation, the dietary intake of n-3 PUFA increased from a baseline level of 1.1 (SD 0.55) energy percent (E%) to 2.9 (0.84) E% (*P* < 0.001) at follow-up, whereas the total dietary intake of n-6 PUFA increased from 3.6 (1.25) E% at baseline to 9.4 (1.64) E% at follow-up (*P* < 0.001) during the n-6 supplementation period (reference).

**Fig. 1:**
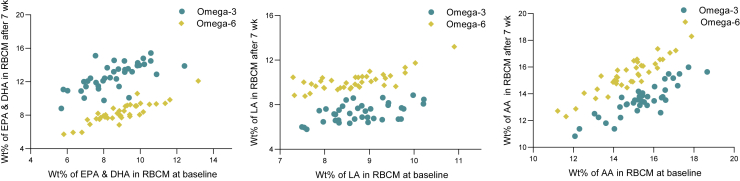
Weight percent (wt%) of EPA/DHA, LA, and ARA FAs in RBCMs at baseline and after 7 weeks of supplementation with n-3 and n-6 PUFAs. FAs were measured by gas-LC in RBCMs isolated from fasting whole blood samples.

At baseline, all participants had an n-3 index (sum of EPA and DH in RBCMs) over 4 wt% and therefore were at low (>8 wt%) or intermediate (4–8 wt%) risk for CVD according to the n-3 index risk zones (82). The wt% of LA in RBCMs were similar to the n-3 index and thus higher compared with other studies (83). Looking at relative changes of FAs in RBCMs, the n-3 index increased by 40.6% (*P* < 0.001) during the n-3 intervention, whereas the level decreased by 3.8% (*P* < 0.001) among the participants receiving the n-6 oil (Fig. 1 and supplemental Table S2). The level of LA in RBCMs increased by 12.5% from baseline (*P* < 0.001) after n-6 supplementation and decreased by 18.2% (*P* < 0.001) after the n-3 intervention. ARA wt% in RBCMs was not significantly changed in relative terms during the n-6 intervention, whereas it decreased by 10.0% (*P* < 0.001) from baseline after n-3 supplementation (Fig. 1 and supplemental Table S2). Overall, the RBCM content of total n-3 PUFAs and n-6 PUFAs was changed by 33.2% (*P* < 0.001) and −12.5% (*P* < 0.001) after n-3 supplementation and by −4.28% (*P* < 0.001) and 4.41% (*P* < 0.001) after n-6 supplementation. Total content of MUFAs was significantly reduced after both interventions (−3.10% [*P* < 0.001] and −4.42% [*P* < 0.001] after n-3 and n-6, respectively), whereas RBCM saturated fatty acids (SFAs) were only significantly changed after n-3 (0.45%, *P* < 0.001) (supplemental Table S2). The relative change in RBCM content of all measured FAs is presented in supplemental Table S2. We also analyzed the wt% of FAs in serum, which reflects the more current intake of the supplemented FAs (supplemental Table S3). Notably, some of the FAs measured in RBCMs, particularly the n-3s, did not fully rebound to the baseline levels, whereas a full rebound was seen in serum (supplemental Fig. S1). Nonetheless, there was a strong correlation between the change in wt % of the FAs in RBCMs and serum (supplemental Table S4).

### Circulatory inflammatory cytokines and adipokines

**Fig. 2:**
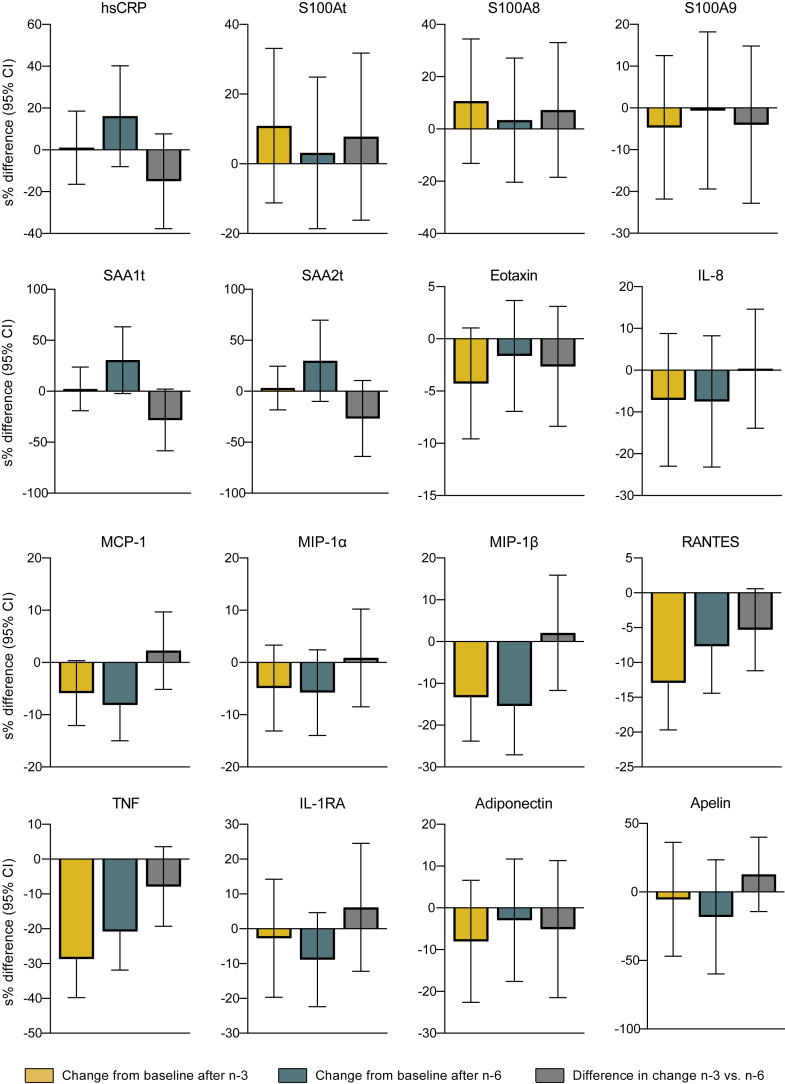
Pooled relative change from baseline (s%) in inflammatory markers. The bar plots show relative changes from baseline to follow-up in acute-phase proteins, cytokines, and adipokines. Data are analyzed with cLMM. Before the analysis, values were transformed by the natural logarithm and multiplied by 100 to show within-group changes and between-group differences as additive, symmetric percentages (sympercents; see main text). Error bars represent 95% CIs. Relative within-treatment changes from baseline to follow-up after the n-3 supplementation are shown in yellow bars and after the n-6 intervention in green bars. Between-treatment differences in relative change scores are shown in light gray bars.

There were no statistically significant between-treatment differences in relative change scores for any of the cytokines or adipokines measured (Table 1 and Fig. 2). However, within-group analysis revealed a significant decrease after both n-3 and n-6 supplementation in MIP-1β, TNF, and RANTES and a significant decrease in MCP-1 after the n-6 intervention only (Table 1 and Fig. 2). Notably, GM-CSF, INF-γ, IL-1β, IL-6, and IL-10 were in most samples not found at detectable levels, neither at week 0 nor at week 7. For acute-phase proteins, there was a notable but nonsignificant increase in CRP, SAAt, SAA1t, and SAA2t after the n-6 supplementation (Table 1 and Fig. 2). Absolute changes in inflammatory markers are presented in supplemental Table S5.

**Table 1:** Relative changes in inflammatory markers after 7 weeks of supplementation with n-3 or n-6 PUFAs^a^

Variable and treatment	Baseline (n = 39)^b^	Follow-up (n = 38)^b^	Relative change^c^	Time^d^	Tx ⨯ time^e^
Acute-phase proteins					
High-sensitive CRP, μg/ml^f^					0.191
n-3	1.66 (0.73, 3.76)	1.73 (0.66, 4.57)	1.02 (−15.2, 20.3)	0.909	
n-6	1.76 (0.73, 4.27)	2.03 (0.77, 5.32)	17.4 (−7.74, 49.4)	0.190	
SAAt, μg/ml^f^					0.249
n-3	2.36 (1.31, 4.24)	2.94 (1.55, 5.58)	6.89 (−14.5, 33.7)	0.557	
n-6	2.94 (1.53, 5.64)	3.74 (1.52, 9.20)	33.0 (−8.14, 92.4)	0.130	
SAA1t, μg/ml^f^					0.068
n-3	1.78 (0.98, 3.27)	2.27 (1.17, 4.39)	2.35 (−17.4, 26.8)	0.830	
n-6	2.25 (1.17, 4.32)	2.85 (1.14, 7.11)	35.6 (−2.20, 88.1)	0.067	
SAA2t, μg/ml^f^					0.158
n-3	0.53 (0.27, 1.04)	0.62 (0.31, 1.24)	3.30 (−16.6, 27.9)	0.764	
n-6	0.64 (0.31, 1.33)	0.83 (0.33, 2.09)	34.9 (−9.41, 101)	0.139	
S100At, μg/ml^f^					0.521
n-3	2.18 (0.84, 5.68)	2.37 (0.94, 5.94)	11.5 (−10.6, 39.2)	0.331	
n-6	2.14 (0.95, 4.82)	2.58 (1.14, 5.80)	3.19 (−16.9, 28.2)	0.775	
S100A8, μg/ml^f^					0.579
n-3	1.45 (0.51, 4.08)	1.60 (0.60, 4.26)	11.2 (−12.3, 41.0)	0.379	
n-6	1.47 (0.63, 3.44)	1.74 (0.73, 4.14)	3.42 (−18.4, 31.1)	0.779	
S100A9, μg/ml					0.671
n-3	0.36 (0.14, 0.91)	0.37 (0.16, 0.87)	−4.56 (−19.6, 13.3)	0.590	
n-6	0.34 (0.15, 0.76)	0.42 (0.19, 0.92)	−0.63 (−17.7, 19.9)	0.947	
Cytokines and chemokines					
Eotaxin, pg/ml					0.365
n-3	52.7 (35.9, 77.3)	50.3 (32.4, 78.1)	−4.18 (−9.13, 1.05)	0.114	
n-6	52.5 (35.7, 77.4)	51.7 (34.3, 77.7)	−1.62 (−6.70, 3.74)	0.544	
IL-8, pg/ml^f^					0.960
n-3	4.06 (2.13, 7.71)	3.73 (2.01, 6.92)	−6.86 (−20.5, 9.14)	0.376	
n-6	3.77 (1.69, 8.38)	3.84 (2.18, 6.76)	−7.20 (−20.7, 8.56)	0.347	
MCP-1, pg/ml					0.544
n-3	43.4 (27.9, 67.5)	41.2 (25.1, 67.8)	−5.69 (−11.4, 0.36)	0.065	
n-6	44.7 (29.7, 67.3)	40.3 (27.1, 60.0)	−7.81 (−13.9, −1.28)	0.020	
MIP-1α, pg/ml^f^					0.853
n-3	1.55 (1.08, 2.22)	1.45 (0.96, 2.19)	−4.77 (−12.3, 3.37)	0.240	
n-6	1.52 (0.98, 2.35)	1.44 (0.93, 2.23)	−5.60 (−13.0, 2.46)	0.166	
MIP-1β pg/ml^f^					0.767
n-3	17.2 (9.27, 31.9)	13.8 (5.77, 33.2)	−12.5 (−21.2, −2.76)	0.014	
n-6	18.1 (10.0, 32.5)	14.5 (6.76, 31.1)	−14.2 (−23.7, −3.61)	0.010	
RANTES, ng/ml					0.077
n-3	10.6 (7.70, 14.6)	9.42 (6.85, 12.9)	−12.1 (−17.9, −6.01)	<0.001	
n-6	10.9 (7.58, 15.7)	9.93 (7.35, 13.4)	−7.37 (−13.4, −0.90)	0.027	
TNF, pg/ml					0.175
n-3	9.54 (6.82, 13.3)	7.13 (4.14, 12.3)	−24.9 (−32.8, −16.1)	<0.001	
n-6	9.45 (6.61, 13.5)	7.72 (4.31, 13.8)	−18.8 (−27.3, −9.24)	<0.001	
IL-1RA, pg/ml^f^					0.509
n-3	134 (69.8, 256)	105 (27.2, 404)	−2.71 (−17.9, 15.2)	0.748	
n-6	103 (27.9, 381)	99.2 (28.2, 349)	−8.51 (−20.1, 4.73)	0.195	
Other adipokines					
Adiponectin, pg/ml^f^					0.540
n-3	97.9 (55.7, 172)	93.0 (52.0, 166)	−7.70 (−20.3, 6.84)	0.280	
n-6	104 (49.6, 217)	97.9 (57.1, 168)	−2.89 (−16.1, 12.4)	0.692	
Apelin, ng/ml^f^					0.352
n-3	183 (48.9, 685)	215 (62.7, 740)	−5.24 (−37.5, 43.7)	0.798	
n-6	205 (52.4, 804)	186 (53.9, 643)	−16.6 (−45.0, 26.4)	0.388	

a Pooled period data of fasting serum and plasma levels were analyzed with cLMM adjusted for the main effect of period and subject-averaged baselines. Values were transformed by the natural logarithm before the analyses.

b Values are geometric means (1 SD ranges) of fasting plasma or serum levels at baseline and follow-up^g^.

c Relative model-adjusted mean change scores (95% CIs) from baseline to follow-up as percentages calculated from the model estimates: % = (exp^estimate^ −1) × 100^g^.

d *P* values for relative changes from baseline to follow-up within treatments (time effects).

e *P* values for relative changes from baseline to follow-up between treatments (group differences in time effects).

f One influential outlier was excluded from the final analyses of high-sensitive CRP, SAAt, SAA1t, SAA2t, S100A8, S100At, IL-8, MIP-1α, MIP-1β, IL-1RA, and adiponectin. Two outliers were excluded from apelin.

g Arithmetic means (SDs) and absolute change scores (95% CIs) from mixed modeling of untransformed data are shown in supplemental Table S5.

Looking at the intervention periods separately (supplemental Table S6), a period-specific response was found in relative change scores for S100At (S100 calcium-binding protein total; P1 vs. P2: −12.6% vs. 66.7%; *P* = 0.004), S100A8 (−17.2% vs. 69.0%; *P* = 0.003), S100A9 (−4.83% vs. 58.0%; *P* = 0.020), and RANTES (−17.6% vs. −0.78%; *P* = 0.005) after the n-6 supplementation and for S100A9 (−15.5% vs. 28.3%; *P* = 0.050), MIP-1β (−4.20% vs. −33.1%; *P* = 0.015), TNF (−14.4% vs. −34.5%; *P* = 0.027), and IL-1RA (−45.7% vs. 15.1%; *P* = 0.024) after the n-3 supplementation.

### Inflammatory cytokines after stratifying participants by low and high RBCM FA status

Because baseline n-3 or n-6 status might have influenced the responses in inflammatory markers, we also performed analyses for participants with high or low baseline n-3 index or LA concentrations in RBCMs (reflecting the intake of different dietary FAs). Participants were stratified by n-3 index <8 wt% (n = 14, “low”) or ≥8 wt% (n = 24, “high”) in RBCMs at baseline and by wt% of LA under (n = 18, “low”) or over (n = 20, “high”) the median LA value (8.88 wt%). Overall, n-6 supplementation lowered several inflammatory markers particularly in participants with a higher n-3 index at baseline (e.g., eotaxin, MCP-1, MIP-1β, TNF, IL-1RA), with a significant response difference between low- and high n-3-index groups for eotaxin and MCP-1 (supplemental Table S7). For n-3 supplementation, baseline n-3 index had less impact (supplemental Table S7 and Supplemental Results), and there were no clear differences in n-3 responses according to a high LA or low LA in RBCMs either (supplemental Table S8). For n-6 supplementation, there were significant within-treatment reductions in TNF, MCP-1, RANTES, and IL-1RA for participants with low baseline LA levels, where the reduction in TNF was significantly different from the lack of change in participants with high LA levels (supplemental Table S8 and Supplemental Results).

### Relationship between inflammatory markers and FAs in the circulation

To further investigate the relationship between PUFA status and inflammatory processes, we analyzed associations between relative change scores of RBCM FAs (reflecting dietary intake) and inflammatory markers. We found significant correlations between change score of circulating TNF and change score of RBCM EPA (r = −0.335, *P* = 0.040), EPA + DHA (r = −0.332, *P* = 0.042), and ARA (*P* = 0.510, r = 0.001) in the participants receiving n-3 PUFAs (supplemental Table S9). After the n-3 intervention, we also found positive correlations between RBCM change score of ARA and circulating RANTES (r = 0.341, *P* = 0.036) and IL-8 (r = 0.419, *P* = 0.01). After n-6 supplementation, we found significant correlations between change score of RBCM EPA and circulating RANTES (r = −0.426, *P* = 0.008), S100At (r = −0.336, *P* = 0.039), S100A8 (−0.338, *P* = 0.038), and adiponectin (r = 0.340, *P* = 0.037), as well as between change score of RBCM ARA and circulating MCP-1 (r = −0.399, *P* = 0.013), RANTES (r = 0.485, *P* = 0.002), S100At (r = 0.411, *P* = 0.010), S100A8 (r = 0.419, *P* = 0.009), and S100A9 (r = 0.375, *P* = 0.020) (supplemental Table S9).

We also correlated the circulatory inflammatory markers with FA concentrations in serum to see if changes in the inflammatory markers also reflected FA changes that returned to baseline by the second supplementation period (supplemental Fig. S1). This analysis showed significant associations after n-3 treatment between change scores of serum LA and circulating SAAt (r = −0.401, *P* = 0.015), SAA1t (r = −0.385, *P* = 0.021), and SAA2t (r = −0.330, *P* = 0.050), and between change scores of serum ARA and circulating IL-8 (r = 0.417, *P* = 0.010) (supplemental Table S10). After the n-6 intervention, we found significant associations between change scores of plasma adiponectin and serum levels of EPA (r = 0.382, *P* = 0.018), DHA (r = 0.363, *P* = 0.025), EPA + DHA (r = 0.394, *P* = 0.014), and LA (r = 0.329, *P* = 0.043). Significant relationships were also found between change scores after n-6 of serum ARA and circulating MCP-1 (r = −0.536, *P* = 0.001), MIP-1α (r = −0.502, *P* = 0.001), and MIP-1β (r = −0.455, *P* = 0.004) (supplemental Table S10).

### Blood pressure and endothelial function

The relative change from baseline to follow-up differed significantly between treatments for systolic blood pressure after nonsignificant changes in opposite directions (reduced and increased after n-3 and n-6, respectively) (Table 2). Diastolic blood pressure, VRI, and heart rate showed small changes from baseline after both treatments, and the relative changes did not differ significantly between treatments. Mixed modeling of period-specific changes showed a notable but nonsignificant between-period difference in VRI after n-3 supplementation (P1 vs. P2: −5.00% vs. 16.7%; *P* = 0.277) and in systolic blood pressure after n-6 (3.92% vs. −1.48%; *P* = 0.055) (supplemental Table S11).

**Table 2:** Relative changes in endothelial function, blood pressure, and pulse after 7 weeks of supplementation with n-3 or n-6 PUFAs^a^

Variable and treatment	Baseline (n = 39)^b^	Follow-up (n = 38)^b^	Relative change^c^	Time^d^	Tx ⨯ time^e^
VRI, index					0.396
n-3	1.38 (0.93, 2.04)	1.44 (0.95, 2.20)	0.56 (−15.8, 20.1)	0.951	
n-6	1.46 (1.05, 2.01)	1.31 (0.74, 2.32)	−7.63 (−22.7, 10.3)	0.378	
Systolic blood pressure, mm Hg					0.003
n-3	116 (105, 130)	116 (104, 129)	−1.81 (−4.62, 1.08)	0.215	
n-6	120 (106, 136)	121 (110, 134)	2.61 (−0.33, 5.63)	0.081	
Diastolic blood pressure, mm Hg					0.786
n-3	73.0 (63.8, 83.6)	73.6 (64.0, 84.6)	−0.71 (−4.39, 3.11)	0.708	
n-6	75.3 (65.8, 86.1)	73.3 (63.3, 84.9)	−1.16 (−4.82, 2.65)	0.543	
Heart rate, beats/min					0.214
n-3	59.1 (51.2, 68.1)	60.7 (52.4, 70.3)	1.20 (−1.58, 4.05)	0.399	
n-6	60.7 (53.4, 69.0)	62.3 (53.3, 72.9)	3.78 (−0.63, 8.38)	0.093	

a Pooled period data of endothelial function, blood pressure, and pulse were analyzed with cLMM adjusted for the main effect of period and subject-averaged baselines. Values were transformed by the natural logarithm before the analyses.

b Values are geometric means (1 SD ranges) at baseline and follow-up^f^.

c Relative model-adjusted mean change scores (95% CIs) from baseline to follow-up as percentages calculated from the model estimates: % = (exp^estimate^ −1) × 100^f^.

d *P* values for relative changes from baseline to follow-up within treatments (time effects).

e *P* values for relative changes from baseline to follow-up between treatments (group differences in time effects).

f Arithmetic means (SDs) and absolute change scores (95% CIs) from mixed modeling of untransformed data are shown in supplemental Table S5.

### Relationships between inflammatory markers and heart rate, blood pressure, and VRI

Because inflammatory factors can alter blood pressure and endothelial function, we performed correlation analyses to investigate possible relationships between these measurements. After n-3 supplementation, we found a negative relationship between change score of VRI and changes in both TNF (r = −0.44, *P* = 0.006) and MIP-1α (r = −0.351, *P* = 0.031) (supplemental Table S12). Following n-3 intervention, we also found positive correlations between change score of heart rate and changes in SAAt (r = 0.437, *P* = 0.008), SAA1t (r = 0.440, *P* = 0.007), SAA2t (r = 0.365, *P* = 0.028), and MIP-1α (r = 0.407, *P* = 0.011) (supplemental Table S12). After the n-6 supplementation, a positive relationship was found between changes in systolic blood pressure and MCP-1 (r = 0.479, *P* = 0.002) (supplemental Table S12).

### FA composition in AT

In AT, we found significant differences between treatments in wt% of the n-3 PUFAs eicosatetraenoic acid (n-3 vs. n-6: +5.29% vs. −1.41%, *P* = 0.005), EPA (+50.2% vs. −1.38%, *P* < 0.001), heneicosapentaenoic acid (+44.3% vs. −2.20%, *P* < 0.001), docosapentaenoic acid (+7.36% vs. −2.00%, *P* < 0.001), and DHA (+16.0% vs. −3.67%, *P* < 0.001), as well as in the SFA myristic acid (+1.17% vs. −1.29%, *P* = 0.007) (supplemental Table S13). These differences arose from significant within-treatment increases after the n-3 intervention. A significant between-group difference was also observed for LA (−0.033% vs. +4.91%, *P* < 0.001), which was significantly increased after the n-6 treatment. The alterations in individual FAs resulted in significant differences between treatments in total n-3 PUFAs (+8.60% vs. −1.67%, *P* < 0.001) and n-6 PUFAs (−0.18% vs. +4.56%, *P* < 0.001) as well as in SFA (+0.86% vs. −0.86%, *P* < 0.001). Furthermore, within-group analyses revealed significant reductions in oleic acid (−0.65%, *P* = 0.001), adrenic acid (−2.91%, *P* = 0.049), and total MUFA (−0.72%, *P* = 0.002) following the n-3 intervention, and in oleic acid (−0.54%, *P* = 0.031) after n-6. Notably, a significant difference between interventions was observed for trans FAs (n-3 vs. n-3: +1.65% vs. −1.87%, *P* = 0.021) due to nonsignificant within-treatment changes in n-3 and n-6 in opposite directions (supplemental Table S13). In correlation analyses between FAs in SAT and RBCMs, there were all over good correlations at baseline and the follow-up values for EPA and DHA, whereas levels of LA and ARA showed weaker correlations between SAT and RBCMs (supplemental Table S14).

### Relationship between inflammatory markers and FAs in AT

Since AT produces and secretes proinflammatory and anti-inflammatory cytokines into the circulation, we furthermore analyzed associations between relative change in wt% of PUFAs in SAT and inflammatory circulating markers (supplemental Table S15). Among the participants receiving n-3, significant positive correlations were found between change score of LA and changes in circulating RANTES (r = 0.539, *P* = 0.007) and adiponectin (r = 0.470, *P* = 0.043) as well as between changes in wt% of ARA and circulating TNF (r = 0.616, *P* = 0.005). After n-6 supplementation, significant correlations were observed between change in circulating eotaxin and change in wt% of EPA (r = −0.537, *P* = 0.022), DHA (r = −0.647, *P* = 0.004), EPA + DHA (r = −0.712, *P* = 0.001), and ARA (r = −0.657, *P* = 0.003), between change in circulating MCP-1 and changes in wt% of DHA (r = −0.591, *P* = 0.010), EPA + DHA (r = −0.625, *P* = 0.006), and ARA (r = −0.588, *P* = 0.001), as well as in change score of IL-1RA and changes in wt% of EPA + DHA (r = −0.511, *P* = 0.036) and ARA (r = −0.539, *P* = 0.026).

### Gene expression in AT

**Fig. 3:**
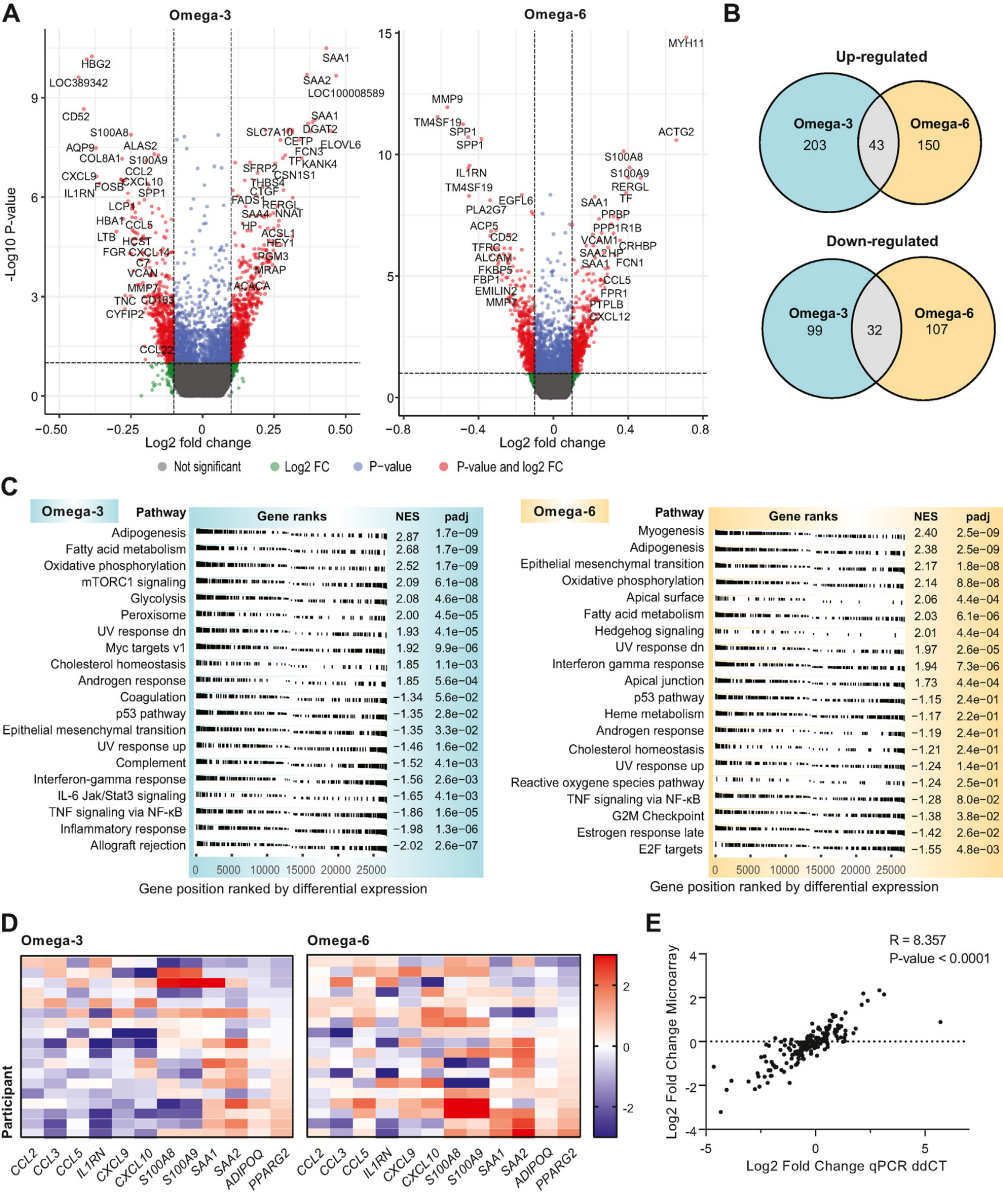
Gene expression in AT after supplementation with n-3 and n-6 PUFAs. A and B: Plot showing upregulated and downregulated genes after n-3 and n-6 interventions. The *x*-axis indicates the differential expression profile, plotting the fold change (FC) in a log_2_ scale. The *y*-axis indicates statistical significance of the difference in expression (*P* value) on a log_10_ scale. Colors represent different genes: Red represents significantly regulated genes with FC ≥10% or FC ≤−10%; blue represents significantly regulated genes with FC ≤10% or FC ≥−10%; green represents genes without significant different expression and FC ≥10% or FC ≤−10%; black represents genes without significantly different expression. B: Venn diagrams illustrating the numbers of upregulated and downregulated genes after the two interventions. C: GSEA of microarray data showing enriched hallmark categories for upregulated and downregulated genes in the groups. D: Heat map showing gene expression measured by qPCR. The genes in the heatmap showed significant change in expression in the RPA from the microarray. Each row represents a single person, whereas each column represents a single gene. The gene expression is shown as FC (log_2_) within each participant. E: Plot showing strong correlation between gene expression measured by qPCR and microarrays. ddCT, delta-delta CT method.

Global gene expression analyses revealed differences in gene expression in AT after supplementation with both n-3 and n-6 PUFAs. Using RPA, 379 differentially expressed genes with false discovery rate <10% and fold change ≥10% or ≤−10% were found following the n-3 intervention, of which 246 were upregulated and 131 downregulated (Fig. 3A). After n-6 supplementation, 334 differentially expressed genes were identified, of which 193 were upregulated and 139 downregulated (Fig. 3A). Venn diagrams (Fig. 3B) showed 43 upregulated and 32 downregulated genes that overlapped in the groups, including *SAA1* (serum amyloid A variant 1), *SAA2* (serum amyloid A variant 2), and *CETP* (commonly upregulated), and *CCL2*, *MMP9*, and *CD52* (commonly downregulated) (supplemental Table S16). We also found several divergently regulated genes after n-3 and n-6 interventions (supplemental Fig. S2), including the inflammatory genes *S100A8*, *S100A9*, and *CCL5* (downregulated in n-3 and upregulated in n-6). The top 50 regulated genes in the two groups are shown in supplemental Table S17.

To further investigate the functions of the differential expressed genes, we subjected the lists of significantly regulated genes to a GSEA, which revealed that multiple processes related to inflammation and lipid metabolism were altered in both groups. After the n-3 intervention, we found significant enrichment for downregulated genes related to several inflammatory pathways, including inflammatory response (normalized enrichment score [NES] = −1.98), TNFα signaling via NF-KB (NES = −1.86), IL-6 JAK/STAT3 signaling (NES = −1.65), and interferon γ response (NES = −1.56) (Fig. 3C). A significant enrichment for downregulated genes was also found in allograft rejection, which had the strongest NES score (−2.02) after n-3 consumption. Among upregulated genes by the n-3 supplementation, significant enrichment was found for several metabolic processes, including adipogenesis (NES = 2.87), FA metabolism (NES = 2.68), oxidative phosphorylation (NES = 2.52), mTORC1 signaling (NES = 2.09), and glycolysis (NES = 2.08).

Contrasting the finding after n-3 supplementation, we found after n-6 consumption evidence for increased inflammatory processes, with enrichment for upregulated genes related to interferon γ response (NES = 1.94) and inflammatory response (NES = 1.6) (Fig. 3C and supplemental Table S18). Also upregulated were, e.g., myogenesis (NES = 2.40), adipogenesis (NES = 2.38), oxidative phosphorylation (NES = 2.14), and FA metabolism (NES = 2.03). Pathways significantly downregulated following the n-6 treatment included E2F targets (NES = −1.55), estrogen response late (NES = −1.42), and G2M checkpoint (NES = −1.38). A list with leading edge genes in the different hallmark categories in the GSEA analysis is provided in supplemental Table S10. GSEA analysis showing significantly enriched categories related to biological pathways are also provided in supplemental Fig. S3.

qPCR analysis showed high consistency with the microarray expression data, confirming both magnitudes of expression changes and a notable interindividual variation in response (Fig. 3D, E). Interestingly, the heat map showed a pattern where participants with increased expression of *PPARG* (encoding peroxisome proliferator-activated receptor-γ) particularly after n-3 supplementation also had a reduced expression of most inflammatory genes measured, and Pearson’s correlation analysis showed a significant relationship between changes in expression level of several of the inflammatory markers and changes in *PPARG* mRNA expression (supplemental Fig. S4).

### Correlations between circulating and adipose inflammatory marker levels

**Fig. 4:**
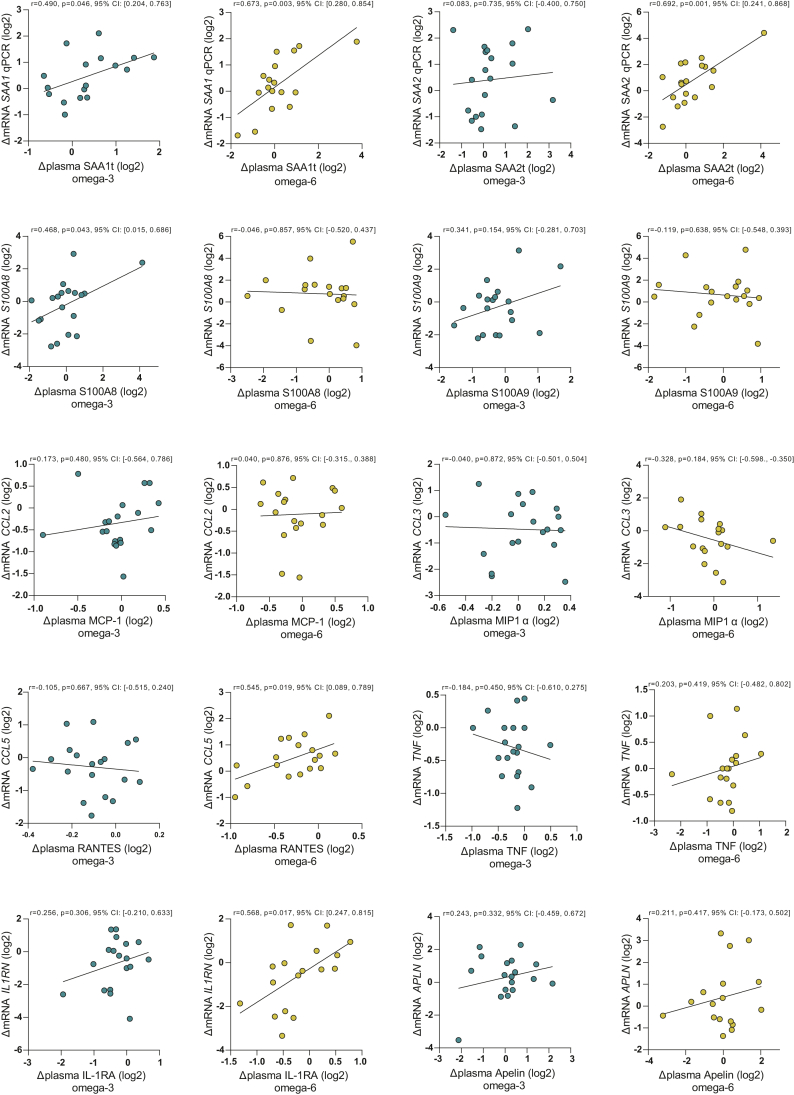
Correlation analyses of changes in circulating levels of inflammatory biomarkers with changes in AT gene expression. Correlation coefficients (95% BCa CIs) were obtained by bootstrapped Pearson’s correlation analyses using fold change values. Data were log_2_ transformed before analysis. One outlier was removed from *SAA1* mRNA and SAA1t, IL-1RA, and apelin in the n-3 group and one outlier from *SAA1* mRNA and apelin in the n-6 group. 95% BCa CI, 95% bootstrapped (bias-corrected and accelerated) CI.

Since we found altered gene expression of several inflammatory genes, we next investigated whether the adipose gene expression correlated with circulating levels of the corresponding factors. Interestingly, in the individuals on n-6 supplementation, the circulating serum level of RANTES was positively correlated with change in gene expression of the RANTES-encoding gene, *CCL5*, in AT (r = 0.545, *P* = 0.019) (Fig. 4). After the n-6 intervention, we also found a positive relationship between change scores of circulating levels of IL-1RA and change in adipose *IL1RN* mRNA (r = 0.568, *P* = 0.017) and changes in circulating levels of SAA1t and adipose *SAA1* mRNA (r = 0.673, *P* = 0.003) and SAA2t and *SAA2* mRNA (r = 0.692, *P* = 0.001) (Fig. 4). After n-3 supplementation, we observed a positive relationship between the change scores of circulating SAA1t and adipose *SAA1* mRNA (r = 0.490, *P* = 0.046) and changes in circulating S100A8 and adipose *S100A8* mRNA (r = 0.468, *P* = 0.043). These data support a contribution by AT to the individual changes in specific circulating inflammatory factors in response to n-3 and n-6 PUFA interventions.

### Cell-autonomous effects in primary human adipose cultures

**Fig. 5:**
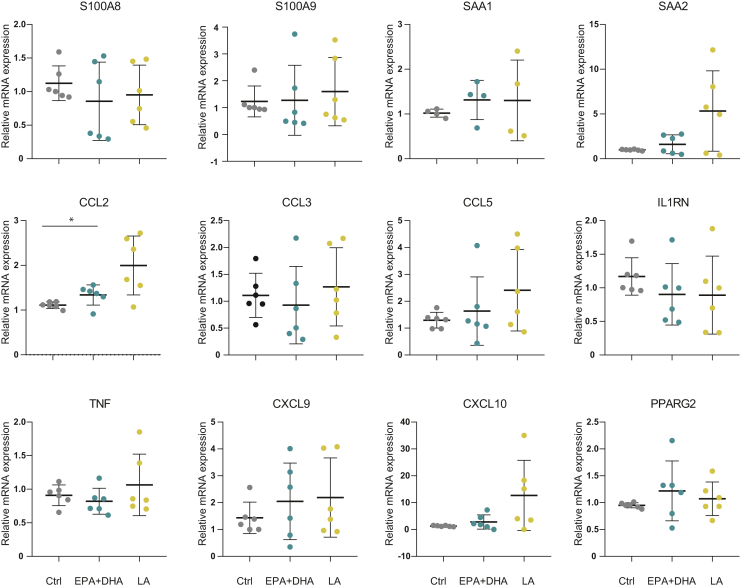
Gene expression of inflammatory markers in FA-treated human adipocytes. Human primary adipose cultures obtained from liposuction material were induced to differentiate for 12 days and treated for 24 h with EPA (100 μM) and DHA (100 μM) in combination or LA (200 μM). mRNA for different inflammatory genes was measured by qPCR calculated relative to *HPRT* mRNA. Data are normalized to the median of the control and presented as mean ± SD. Experiments were performed in triplicates from six different individuals. Ctrl, control; *HPRT*, hypoxanthine phosphoribosyltransferase.

To investigate possible direct effects of FA supplementation in adipocytes, we treated in vitro differentiated human adipocyte cultures from six different individuals with EPA/DHA or LA. For all the cultures combined, we observed a significant upregulation of *CCL2* expression in cells treated with LA compared with controls (Fig. 5). Although mRNA for *SAA2*, *CCL5*, and *CXCL10* showed a similar tendency of an increase with LA treatment, no significant changes were found after treatment in any of the other genes measured.

## Discussion

In secondary analyses of this randomized double-blind crossover trial, we here investigated whether supplementation with high quality n-3 (fish oil) or n-6 (safflower oil) PUFAs affected inflammatory factors, as well as blood pressure, endothelial function, and adipose FAs and gene expression, in healthy but physically inactive people with abdominal obesity. Both interventions overall gave reductions in circulating levels of inflammatory markers, including RANTES, TNF, and MIP-1β after n-3 supplementation and RANTES, TNF, MIP-1β, and MCP-1 after n-6 treatment. The respective interventions also resulted in significant increases in the proportion of n-3 and n-6 FAs in subcutaneous AT, supporting effects in this tissue. But in contrast to suppressive or neutral effects of n-3 in AT, n-6 (LA) supplementation was followed by upregulation of adipose inflammatory gene expression including mRNA for RANTES (*CCL5*) and with similar tendencies in LA-treated cultured human primary adipocytes including a significant upregulation of *CCL2*. Moreover, a significant difference between treatments was observed in systolic blood pressure, owing to a slight nonsignificant reduction and increase following n-3 and n-6, respectively.

While some other studies have suggested proinflammatory effects of increased LA intake, linked to a potential increased formation of ARA (25, 41, 42, 43), we found no evidence of increased circulatory inflammatory markers following the high-dose high-quality LA supplementation. Notably, the wt% of ARA in RBCMs, serum, or AT in our study were overall not changed after the increased LA intake. This is consistent with a systematic review of 36 RCTs, which found no relationship between intake of LA and changes in ARA in the phospholipid pool of plasma/serum (84), and with human isotope tracer studies showing low enzymatic conversion rates (under 1%) of dietary LA to ARA (47, 85). Moreover, we found that increased LA intake lowered the levels of several circulating inflammatory markers, including MCP-1, MIP-1β, RANTES, and TNF. Previous RCTs performed on healthy people showed no significant changes in inflammatory markers (50, 51, 53, 54, 56, 57, 58, 59, 61, 62, 63), except two that reported lower circulating IL-1RA and TNF-R2 levels after 6 weeks of sunflower oil supplementation (60) and lower IL-6, TNF, and sVCAM after a high-PUFA meal (86). Taken together, our study does not support systemic proinflammatory effects but rather an overall anti-inflammatory potential of n-6 PUFAs in people with abdominal obesity.

In comparison to studies on n-6 PUFAs, numerous clinical studies have investigated the effects of n-3 PUFAs on inflammation. Although n-3 PUFAs are considered to reduce inflammation, with several observational, in vivo and in vitro studies showing anti-inflammatory properties (45, 87, 88, 89, 90), results are inconsistent, especially in healthy people (30, 31, 32). This is probably due to methodological differences, including high variance in duration of supplementation, treatment groups, doses, and oil compositions. Few studies have been performed in individuals with abdominal obesity (65, 91, 92), and to our knowledge, the present study is the first that directly compares n-3 and n-6 supplementation in people with abdominal obesity. Similar to n-6 supplementation, we found reduced levels of several inflammatory markers after n-3 supplementation, including in the cytokines TNF, RANTES, and MIP-1β. A study by Chan *et al.* (65) comparing the effects of supplementation for 6 weeks with statins and/or 4 g fish oil (45% EPA and 39% DHA) showed no effect on CRP, TNF, or IL-6 in the fish oil group. Another study investigating the effect of 2 g fish oil (649 mg EPA and 480 mg DHA), compared with placebo (olive oil) in individuals with visceral obesity, also showed no effect on IL-6, TNF, TNF receptor 1 and 2, or CRP after 6 weeks with supplementation (92). However, a study comparing EPA (2.7 g) and DHA (2.7 g) in individuals with abdominal obesity and low-grade systemic inflammation found lowering of CRP, IL-6, IL-18, and TNF, where DHA was more potent than EPA (91).

This is the first RCT investigating and reporting reduced circulating levels of RANTES and MIP-1β (CCL4) after n-3 supplementation in healthy people. The few studies investigating RANTES to date, which were performed in patients with chronic kidney disease or coronary heart disease and with different dosages, have shown inconsistent results (93, 94). However, our findings are supported by some previous in vitro and in vivo (e.g., mouse) studies reporting lower RANTES after treatment with n-3 PUFAs (95, 96, 97, 98). Also, a recent RCT in patients with Alzheimer’s disease found reduced circulatory MIP-1β levels after n-3 treatment for 24 months (99). Moreover, the decrease in TNF agrees with several ex vivo studies reporting lower TNF production from endotoxin-stimulated monocytes or mononuclear cells after providing EPA and DHA to healthy individuals (100, 101, 102, 103). Taken together, our results are consistent with other studies showing anti-inflammatory effects of n-3 and data showing an inverse relationship between the n-3 index and markers of systemic inflammation (104).

One of the proposed mechanisms behind the anti-inflammatory effect of n-3 PUFAs is the replacement of ARA in cell membranes, as increased consumption of EPA and DHA results in a dose-dependent incorporation into phospholipids partly at the expense of ARA (25). Furthermore, EPA has been shown to inhibit the metabolism of ARA, resulting in a decreased production of ARA-derived eicosanoids (25, 49). In our study, after n-3 supplementation, we found a 11.5% reduction of ARA in the RBCMs concomitant with a 48.4% increase of EPA and DHA, where the reduction of ARA in cell membranes was positively correlated with the lowering of TNF and RANTES. However, because we also found a reduction of TNF and RANTES after n-6 supplementation, where the level of ARA was overall unaltered, other mechanisms independent of the synthesis of eicosanoids were likely also involved, at least following the n-6 treatment.

While the PUFA supplementation lowered several of the measured inflammatory markers (e.g., TNF, RANTES, MIP-1β), the lack of change in other markers (e.g., CRP, IL-8, MCP-1, MIP-1α, eotaxin) might be due to the high n-3 index among the participants in our study at baseline and relatively low baseline inflammatory marker levels, giving a lower potential for change. Notably, however, reductions after n-6 supplementation were generally more pronounced in participants with high n-3 status or low LA status assessed in RBCMs. Along the same lines, higher n-3 intake has been suggested to attenuate the proinflammatory potential of a high n-6 intake (105). Taken together, the data point to an importance of sufficient n-3 intake for optimal anti-inflammatory effects of n-6 intake and a limited anti-inflammatory potential of LA beyond a certain status level.

When correlating the changes in PUFA concentrations in RBCMs or serum with circulating inflammatory markers, regardless of supplementing n-3 or n-6, we found negative correlations between the n-3 PUFAs in RBCMs and TNF (n-3 treatment) or RANTES, S100At, and S100A8 (n-6 treatment). Additionally, specifically for the n-6 treatment, adiponectin showed a positive correlation with the n-3 PUFAs in both RBCMs and serum and with LA in serum. These data are consistent with a suppressive effect of n-3 on inflammatory markers. Interestingly, for participants supplementing n-3, LA in serum (but not RBCMs) showed an inverse correlation with SAAt, SAA1t, and SAA2t, suggesting that anti-inflammatory effects of n-3 might in part occur via altered circulating LA levels. Conversely, for both the n-3 and n-6 treatment, there were positive correlations between ARA in RBCMs or serum and inflammatory markers (RANTES and IL-8 for n-3 treatment; RANTES, S100At, and S100A8 for n-6 treatment), supporting that ARA is associated with proinflammatory factors. Yet, for the n-6 treatment, specifically there were also inverse correlations between ARA in RBCMs and MCP-1 and between ARA in serum and MCP-1, MIP-1α, and MIP-1β. The reason for and relevance of these opposite correlations between ARA and specific inflammatory markers requires further investigation.

It has been suggested that PUFAs have favorable effects on vascular function (106). Cross-sectional studies have found that dietary intake of both n-3 and n-6 PUFAs is associated with a lower prevalence of hypertension (107), but few RCTs have compared the effects of supplementation with n-3 and n-6 PUFAs on blood pressure in humans (108, 109). In our study, carried out in normotensive individuals, we found that modest nonsignificant changes in opposite directions (decrease after n-3 and increase after LA) resulted in a significant between-treatment difference in systolic blood pressure, which may be clinically significant. This is in line with an RCT in 33 normotensive and mildly hypercholesterolemic males, which found a reduction in systolic blood pressure (-5 mm Hg) after treatment with 3.4 g EPA/DHA when compared with treatment with 14.3 g LA (109). Furthermore, meta-analyses of RCTs have shown a lowering effect of EPA and DHA on both systolic and diastolic blood pressure (110, 111, 112, 113). However, most of these studies have been performed in hypertensive, elderly, and/or for a longer intervention period, and it is therefore possible that a larger effect had been observed in our study with a longer duration. Less information is so far available regarding the effect of LA on blood pressure. Although cross-sectional studies have shown an inverse relationship between LA intake/plasma levels and blood pressure (114, 115), the lack of a significant change in blood pressure after n-6 supplementation in our study is in line with two other RCTs investigating n-6 PUFA intake in normotensive subjects (116, 117).

In contrast to blood pressure, we observed no significant differences between treatments in endothelial function measured by VRI. Regarding n-3 PUFAs, this is consistent with a placebo-controlled clinical trial where 3.4 g/day EPA and DHA reduced TAG but failed to reduce flow-mediated dilation (FMD) in healthy women with moderate hypertriglyceridemia (118). However, two meta-analyses of RCTs found significant improvements in endothelial function at dosages ranging from 0.45 to 4.5 g/day (119, 120). In a subgroup analysis in one of these studies (120), improvement in endothelial function was only found in participants with poor health. The authors suggested that improvement in endothelial function might be limited in healthy individuals, which might also explain the lack of effects in our study. Of note, although no significant change was observed in VRI, a negative correlation was found for changes in both TNF and MIP-1α with changes in VRI after the n-3 intervention, suggesting that the anti-inflammatory effect of n-3 supplementation might be involved in improving endothelial function in abdominal obesity, at least in some individuals.

Studies investigating the effect of n-6 PUFAs on endothelial function are however limited (121). An RCT supplementing with 30 ml sunflower oil in elderly people with overweight or obesity for 90 days found improved FMD (122). However, in the DIVAS study comparing diets high in SFA, MUFAs, or n-6 PUFAs in 195 males and females with moderate CVD risk, no effect was seen on FMD or other measurements of vascular reactivity (61), in line with the lack of effect in our study. Our study also allowed direct comparison of how n-3 and n-6 supplementation affects endothelial function. In another study, 8 months' consumption of six different oils containing n-3 and/or n-6 was compared in 173 males and females, although at lower doses than in our study. Beneficial effects were observed in the group that increased the intake of n-3, which may have been due to the longer duration of that study compared with ours (123). Taken together, our data suggest that the potential of PUFAs to improve endothelial function may be limited, at least in normolipidemic and normotensive people.

The effect of PUFAs on AT biology in humans is poorly characterized (124). The high-dose n-3 and n-6 supplementation in the 7-week period in our study gave increased levels of the respective PUFAs in AT, supporting a lasting impact on tissue function and that FAs in AT reflect dietary intake (125). A shorter-term change in adipose FA composition is supported by a previous study of five healthy subjects, 20–22 years of age, showing a significant increase in LA levels in AT beginning already after 2 weeks on a diet high in safflower oil, and a gradual increase until the study’s end at day 35 (126). Another study of 26 patients with type 2 diabetes found that supplementation with fish oil (5.9 g n-3 FA) or corn oil (8.5 g LA) for 9 weeks significantly increased AT levels of EPA and DHA but not LA (127). However, a higher dose of LA was supplemented in our study. After the n-3 supplementation and corresponding increase in adipose EPA and DHA, we found a downregulation of inflammatory processes, in particular interferon-γ response, TNF-signaling via NF-κB, and inflammatory response, in line with previous RCTs measuring gene expression of inflammatory genes (128, 129, 130, 131), as well as animal studies (132, 133, 134). We also found correlations between changes in *SAA1* and *S100A8* mRNA in AT and serum concentrations of their respective proteins, suggesting that n-3 might affect the circulatory levels via AT. Regarding n-6 intake, previous studies found no impact on expression of AT gene expression in humans (60) or mice (135) for a selected few inflammatory genes. In our study, however, even though circulatory markers were decreased after the LA supplementation, AT showed upregulation of several pathways linked to inflammatory responses. This disconnect between inflammatory profiles in AT and the circulation might involve cell type-specific metabolism of LA, as observed in hepatocytes and monocytes (136), and/or a tissue-specific adaptive response in the integrated regulation of inflammation and metabolism (137). At the same time, we found positive correlations between changes in *IL1RN*, *CCL5*, *SAA1*, and *SAA2* mRNA in AT and circulating levels of their corresponding proteins, implying that the local effects in AT contributed to circulating levels of at least some of the inflammatory markers also after the n-6 intervention.

The effects on inflammatory genes can be interpreted in light of the observed changes in key metabolic processes, such as those related to PPARγ. This nuclear receptor, and direct target of both n-3 and n-6 PUFAs, is primarily expressed in adipocytes, and controls key functions of AT including lipid and glucose metabolism, as well as inflammation by reducing the expression of NF-κB in response to PUFAs (138, 139). In our study, *PPARG* mRNA and several PPARγ targets were among the upregulated genes in the microarray after treatment with both n-3 and n-6, although n-3 appeared to have a slightly stronger effect. Changes in expression of several of the inflammatory genes, particularly after the n-3 intervention, were strongly correlated with change in *PPARG* expression, implying that PPARγ was involved in the alteration of inflammatory response. Moreover, among the most upregulated categories in both groups were adipogenesis, which also is a process known to be promoted by PPARγ resulting in a healthier AT (140). Elevations of both n-3 and n-6 FAs in SAT have been linked to reduced adipocyte size (141). Also upregulated in both groups were several known PPARγ-modulated categories in lipid metabolism, including FA metabolism and TAG synthesis (142). Importantly, *SAA* was among the most affected genes after both n-3 and n-6 PUFA supplementation in our study, which was also related to changes in *PPARG*. This is consistent with earlier studies showing that SAA can increase PPARγ activity (143). Interestingly, SAA marks a functionally distinct subtype of white adipocytes (144, 145), possibly implying that the PUFA supplementation may have affected this subtype in particular. SAA is mainly secreted by the liver in the acute phase of inflammation, while adipocytes are the main source in the obese state (146). In agreement with our study, previous studies have shown an increased expression of *SAA1* after treatment with n-3 (EPA or DHA) or n-6 (ARA) in cultured human adipocytes (147, 148). These data overall support important metabolic effects of the PUFAs in human AT and modest proinflammatory effects of n-6 locally in AT despite a reduction in circulatory inflammatory markers.

The study has several limitations that need consideration. First, period-specific effects were found for some inflammatory markers, including in RANTES and calprotectin after n-6 treatment. This might be related to potential carry-over effects from n-3 supplementation in the first period into the second period, as the change in levels of n-3 in RBMCs did not rebound completely to the pretreatment levels after the washout phase (61), although we here show a complete rebound of the FA levels in serum. Also, the statistical approach (cLMM) inherently renders our analysis less vulnerable to baseline levels (74, 76, 77), and as recommended for crossover trials (149) (but unfortunately often not done (150)), we have presented results for each intervention in each period to help understand any treatment-by-period interactions. Second, the amount of oil was different for n-3 and n-6 due to their different potencies, but this might have affected the results as the energy content was slightly higher for the n-6 intervention. However, in secondary analyses adjusting for BMI, total energy intake, and hormones (estrogen, testosterone, and vitamin D_3_), the nominal significance was not changed for treatment differences in any variables except for MIP-1β, which no longer was reduced significantly in either group. Third, we do not know to what extent tocopherol in the safflower oil might have contributed to the reduction in circulatory inflammatory markers (151). Fourth, in the cell culture experiments, lower, more physiological doses of FAs, and BSA or oleic acid as a control instead of ethanol, may have affected the results. Fifth, the limited number of participants (n = 38) (although relatively large for a crossover study), the short duration, and the specific study population (including a higher percentage of males) limit the generalizability of our results. Finally, we did not perform a power calculation for the complex combination of outcomes variables, and the present study should be considered exploratory.

Among the strengths of our study were the careful randomized double-blind crossover design, the analysis of FA profile in both RBCMs and serum, which indicated good adherence to the intervention, and the availability of AT samples used for detailed gene expression analyses and FA profiling. We also measured a greater panel of markers than many other studies, which may better reflect the overall inflammatory response. Importantly, oil quality was emphasized, and analyses of oxidation products and the levels of FFAs showed that the oils were of high quality (48). In addition, participants were not allowed to use drugs (with a few exceptions), which also lowered confounding factors on treatment effects.

## Conclusion

In conclusion, supplementation with high doses of high-quality oils rich in n-3 and n-6 PUFAs similarly decreased circulating inflammatory markers in individuals with abdominal obesity, although the n-6 supplementation increased expression of several inflammatory genes locally in AT. Overall, the systemic anti-inflammatory effects might represent possible mechanisms for the favorable effect of n-3 and n-6 PUFAs in the prevention of cardiometabolic diseases.

## Data availability

The data that support the findings of this study are available on request from the corresponding author. The data are not publicly available due to privacy or ethical restrictions.

## Supplemental data

This article contains supplemental data (152).

## Conflict of interest

The authors declare that they have no conflicts of interest with the contents of this article.
