# Supplementary material for: Inflammatory markers after supplementation with marine n-3 or plant n-6 PUFAs: A randomized double-blind crossover study
Source: J Lipid Res. 2025 Mar 8;66(4):100770. doi: 10.1016/j.jlr.2025.100770 (PMC11999210; doi:10.1016/j.jlr.2025.100770)
Supplement: Supporting information [file mmc1.pdf]

Supporting information

Changes in circulatory and adipose inflammatory markers, endothelial function and blood pressure after supplementation with n-3 or n-6 PUFAs: A randomized double-blind crossover study

Elise Grytten [a, b], Johnny Laupsa-Borge [a, b, d], Kaya Cetin [a, b], Pavol Bohov [c], Jan Erik Nordrehaug [c, e], Jon Skorve [c], Rolf K, Berge [c], Elin Strand [c], Bodil Bjørndal [c], Ottar Nygård [c, e], Espen Rostrup [e], Gunnar Mellgren [a, b], Simon N, Dankel [a, b,*]

[a] Hormone Laboratory, Department of Medical Biochemistry and Pharmacology, Haukeland University Hospital, 5021 Bergen, Norway (EG, JLB, GM, SND).

[b] Mohn Nutrition Research Laboratory, Department of Clinical Science, University of Bergen, 5020 Bergen, Norway (EG, JLB, GM, SND, OKN).

[c] Department of Clinical Science, University of Bergen, 5020 Bergen, Norway (PB, BB, ES, JS, JEN, RKB).

[d] Bevital AS, Bergen, Norway (JLB).

[e] Department of Heart Disease, Haukeland University Hospital, 5021 Bergen, Norway (JEN, ER, OKN),

***Corresponding author:** Simon N, Dankel, Department of Clinical Science, University of Bergen, Jonal Lies Vei 91, 5021 Bergen, Norway, +4794308637, simon.dankel@uib.no

Contents

Supplementary Methods 5

Supplemental Results 6

Supplemental Figures 7

Supplemental Figure 1. Period-specific relative changes from baseline in fatty acids measured in RBCMs and serum 7

Supplemental Figure 2. Oppositely regulated genes in adipose tissue after 7 wk supplementation with n-3 (omega-3) and n-6 (omega-6). 8

Supplemental Figure 3. Gene set enrichment analysis (GSEA) showing enriched gene ontology categories for up- and down-regulated adipose genes after 7 wk supplementation with n-3 (omega-3) or n-6 (omega-6). 9

Supplemental Figure 4. Correlation of changes in adipose mRNA expression of inflammatory markers and PPARγ2 mRNA after 7 wk supplementation with n-3 (omega-3) or n-6 (omega-6) 9

Supplemental Tables 10

Supplemental Table 1. Primers used for qPCR 10

Supplemental Table 2. Relative changes in fatty acid levels in RBCMs after seven wk of supplementation with n-3 or n-6 PUFAs[1] 11

Supplemental Table 3. Relative changes in fatty acid levels in serum before and after seven wk of supplementation with n-3 or n-6 PUFAs[1] 15

Supplemental Table 4. Correlations between fasting fatty acid levels in serum and RBCMs[1] 21

Supplemental Table 5. Absolute changes in inflammatory markers after 7 wk of supplementation with n-3 or n-6 PUFAs [1] 22

Supplemental Table 6. Period-specific responses in relative changes for circulating levels of inflammatory markers after 7 wk of supplementation with n-3 or n-6 PUFAs[1] 24

Supplemental Table 7. Relative changes of circulating levels of inflammatory markers after 7 wk of supplementation with n-3 or n-6 stratified by n-3 index in RBCMs[1] 27

Supplemental Table 8. Relative changes of circulating levels of inflammatory markers after 7 wk of supplementation with n-3 or n-6 stratified by LA level in RBCMs[1] 31

Supplemental Table 9. Correlation analysis matrix between changes in fatty acid profile in RBCMs and circulating inflammatory markers after 7 wk supplementation with n-3 or n-6 34

Supplemental Table 10. Correlation analysis matrix between changes in fatty acid profile in serum and circulating inflammatory markers after 7 wk supplementation with n-3 or n-6 36

Supplemental Table 11. Period-specific responses in relative changes in endothelial function, blood pressure, and pulse markers after 7 wk of supplementation with n-3 or n-6 PUFAs[1] 38

Supplemental Table 12. Correlation analysis matrix between changes in blood pressure, pulse and VRI and inflammatory markers after 7 wk supplementation with n-3 or n-6 39

Supplemental Table 13. Relative changes in subcutaneous adipose tissue fatty acid levels after seven wk of supplementation with n-3 or n-6 PUFAs [1] 41

Supplemental Table 14. Correlations between fasting fatty acid levels in SAT and RBCMs[1] 44

Supplemental Table 15. Correlation analysis matrix between changes in fatty acid profile in subcutaneous adipose tissue (SAT) and circulating inflammatory markers after 7 wk supplementation with n-3 or n-6 46

Supplemental Table 16. Genes regulated in the same direction in adipose tissue after 7 wk supplementation with n-3 or n-6 (q-value > 0.1, FC ≥ 0.1 or ≤ −0.1) 48

Supplemental Table 17. Top 50 up- and down-regulated genes in adipose tissue after 7 wk supplementation with n-3 or n-6 49

Supplemental Table 18. Top up- and down-regulated “hallmark gene sets” from Gene Set Enrichment Analysis (GSEA) in adipose tissue after 7 wk supplementation with n-3 or n-6............ 53

Abbrevations:

CRP, c-reactive protein; SAAt, serum amyloid A total; SAA1t, serum amyloid A 1 total; SAA2t, serum amyloid A 2 total; S100At, S100 calcium-binding protein total; S100A8, S100 calcium-binding protein A8; S100A9, S100 calcium-binding protein A9; IL-8, interleukin-8; MCP-1, monocyte chemoattractant protein-1; MIP-1α, macrophage inflammatory protein-1α; MIP-1β, macrophage inflammatory protein-1β; RANTES, Regulated upon Activation, Normal T cell Expressed and Secreted; TNF, tumor necrosis factor; IL-1RA, interleukin-1 receptor antagonist.

Supplementary Methods

Missing data handling

Left-censored missing values caused by lower than the limit of detection or quantification, which can be considered as missing not at random, were imputed by the GSimp method (82). GSimp is a Gibbs sampler-based left-censored missing value imputation procedure that utilizes the predictive information of other variables by employing a prediction model and held a truncated normal distribution for each missing element simultaneously. Missing values were initialized by quantile regression imputation of left-censored data (QRILC). We natural log-transformed data before QRILC was conducted to improve the imputation accuracy and ensure positive values in the original scale after back-transformation. Elastic net from the R package glmnet v 4.1-4 was used as the prediction model. The minimum observed value of missing variable was applied as an informative upper truncation point and -Inf as a non-informative lower truncation point for left-censored missing (82).

Stratification analyses

To investigate if different background dietary intakes of PUFAs differently influenced the responses in inflammatory markers, participants were stratified by the relative content of supplemental PUFAs in RBCMs at baseline, i.e., n-3 index <8 wt% ("low") or ≥8 wt% ("high") in one stratification analysis (83), and wt% of LA under ("low") or over ("high") the median LA value (8.88 wt%) in another secondary analysis. The mixed modeling was similar to the primary analysis except for the fixed effects structure, which here also included a binary coded stratification factor (0: low; 1: high) and its interaction with 'time'.

Secondary analyses

In a separate model, we adjusted for covariates that can differ during the trial (period level factors). Total energy intake and BMI were controlled for due to different amounts of oils between the two study arms (5.6/7.5 mL fish oil vs. 24.5/32.7 mL safflower oil). Additionally, we adjusted for sex hormones, including estrogen and testosterone, because these steroids are known to influence the metabolism of n-3 and n-6 PUFAs, and hormone levels may vary across the intervention periods. We furthermore adjusted for vitamin D_3 to account for potential seasonal tissue variations. Since the amount of some FAs in RBCMs did not rebound to the baseline levels after the first intervention period, and thus differed across baseline visit 1 and 2, we adjusted for the n-3 index and wt% of LA in RBCMs at baseline in another separate model.

Supplemental Results

Stratification by high or low n-3 or n-6 in red blood cell membranes at baseline

When stratifying by n-3 index, significant differences in change score were found following the n-6 intervention for eotaxin (low vs. high n-3 index: +3.42% vs. −2.64%, p = 0.030), MCP-1 (−0.30% vs. −6.07%, p = 0.050), and apelin (+428% vs. −57.6%, p = 0.030) (**Supplemental Table 7**). Moreover, no significant differences were found between the stratified n-3 index levels, where n-6 supplementation affected the markers in similar directions for participants with both low and high n-3 index, with significant within-treatment changes reductions after n-6 supplementation among those with high n-3 index for MCP-1, MIP-1β, RANTES, and TNF (reduced), and for CRP, SAAt, SAA1t, and SAA2t (increased). Among the participants receiving n-3, a significant reduction was observed for adiponectin only among the participants with n-3 index <8 wt%, although there was no significant difference between low and high n-3 index (−19.3% vs. −2.49%, p = 0.112) (**Supplemental Table 7**).

When stratifying by baseline LA level, a significant difference after n-6 supplementation was observed in change score of TNF (low vs. high LA level: −1.99% vs. −0.48%, p = 0.026) after a larger reduction among the participants with low LA level (**Supplemental Table 8**). Also, although there were no significant differences in LA levels, significant within-treatment reductions in MCP-1, RANTES, and IL-1RA were only observed among the low LA level participants following the n-6 supplementation. After the n-3 treatment, no significant differences in change scores were found between the baseline LA levels. However, a significant within-treatment reduction in RANTES was only significant for the participants with low LA level, while significant reductions in MIP-1β and TNF were found among participants with both low and high LA levels. Adiponectin was on the other hand only significantly lowered among the participants with high LA level (**Supplemental Table 8**).

Covariate analyses

When analyzing possible covariate effects, the model adjusting for BMI, total energy intake and hormones (estrogen, testosterone, and vitamin D_3) gave no differences in nominal significance for between-treatment differences for inflammatory markers, endothelial function, or blood pressure (data not shown). However, the within-treatment analysis showed that there was no longer a difference in nominal significance for MIP-1β after n-3 (−9.03% [−20.4, 3.92], p = 0.161) and after n-6 (−11.4% [−23.3, 2.32], p = 0.098), while adiponectin now showed a significant decrease (−15.2% [−27.1, −1.43], p = 0.032) after n-3. When controlling for n-3 index and LA in RBCMs at baseline, no change in nominal significance was observed, except for a significant within-treatment reduction in MCP-1 after n-3 (−6.05% [−11.6, −0.19], p = 0.043).

Supplemental Figures

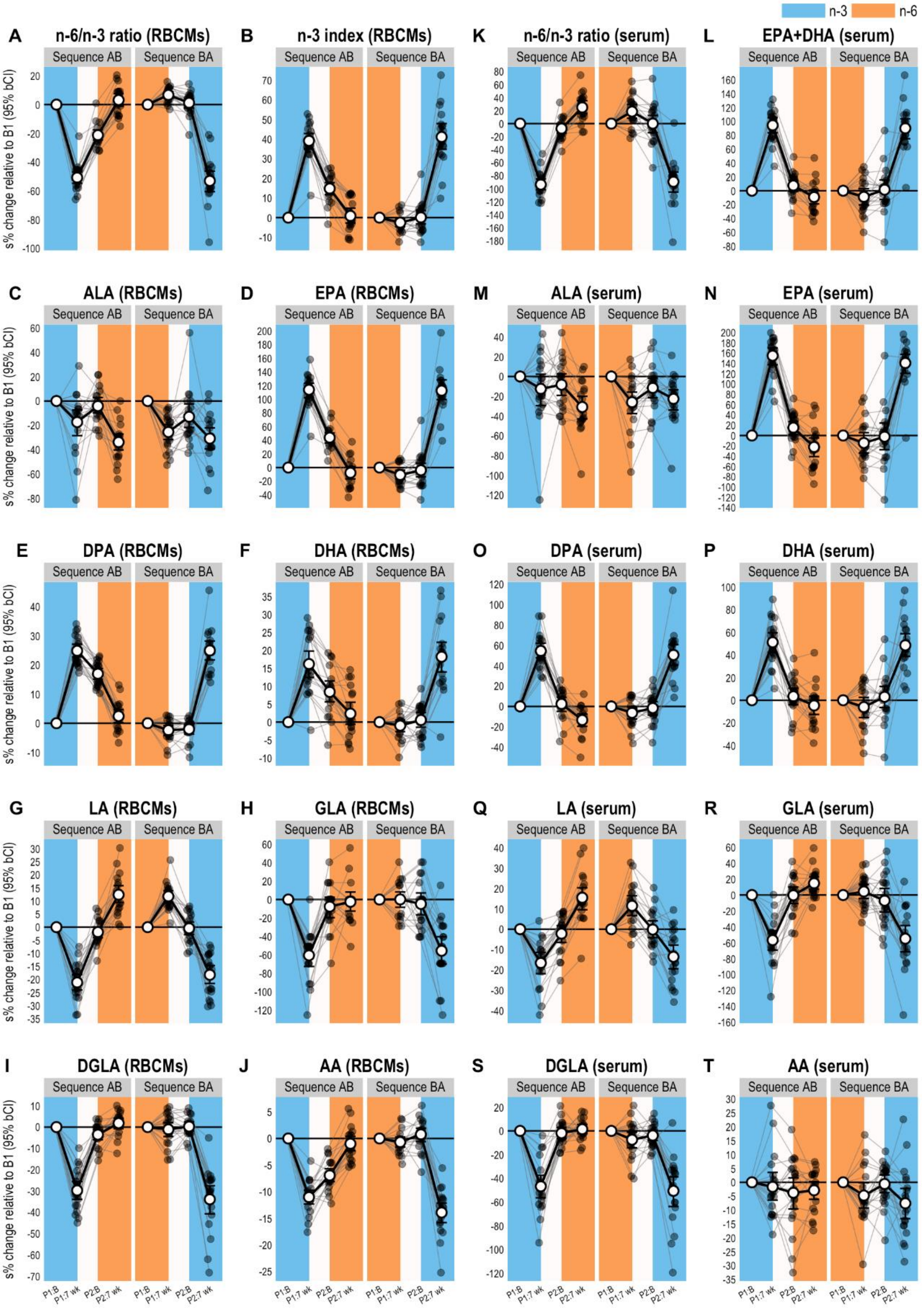

Supplemental Figure 1. Period-specific relative changes from baseline in fatty acids measured in RBCMs and serum. The bar plots show relative changes from baseline to follow-up within periods for each study participant. Abbreviations: P1:B, period 1 baseline; P1:7wk, period 1 7-week follow-up; P2:B, period 2 baseline; P2:7wk, period 2 7-week follow-up; RBCMs, red blood cell membranes; Sequence AB, n-3 in period 1 followed by n-6 in period 2; Sequence BA, n-6 in period 1 followed by n-3 in period 2; s%, sympercents.

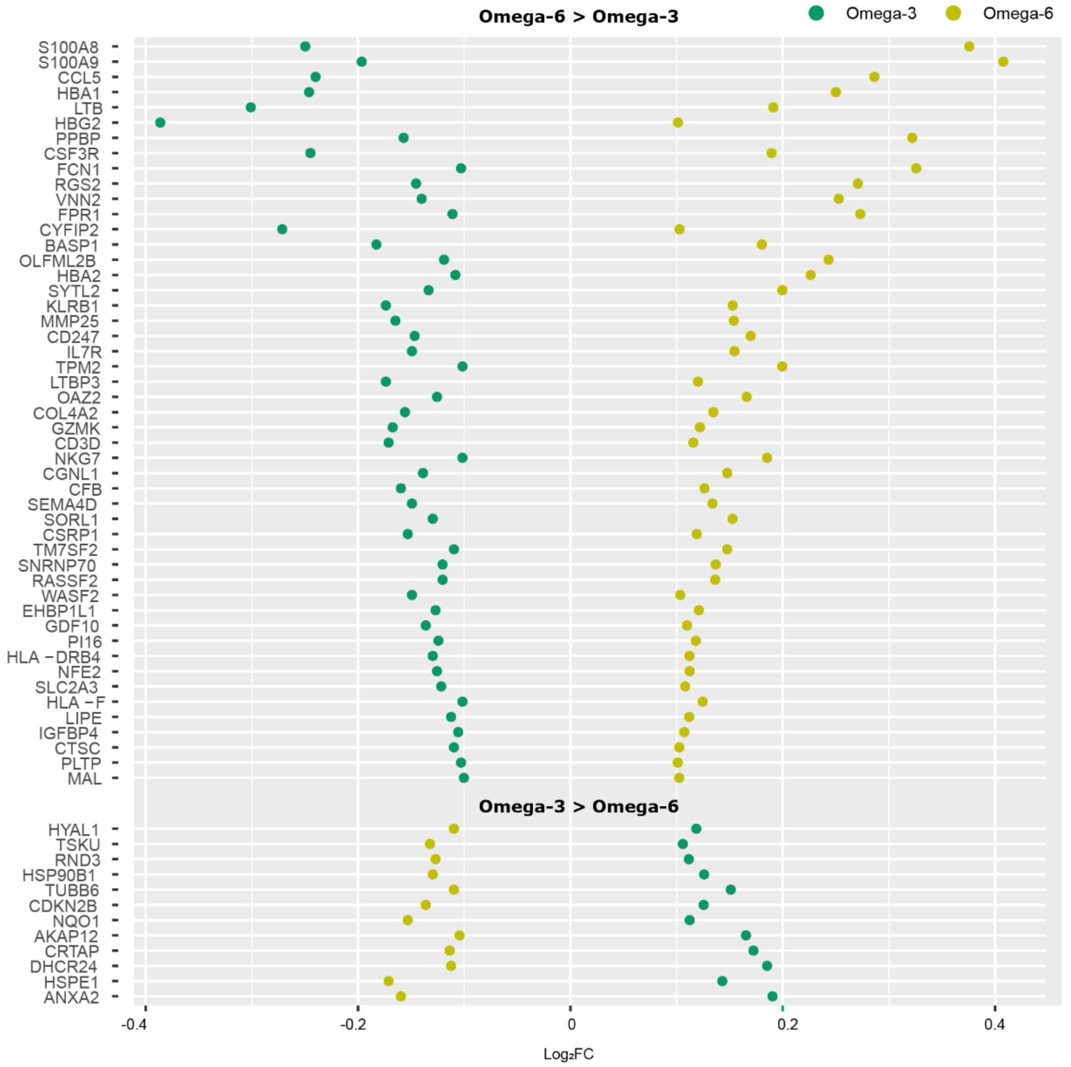

Supplemental Figure 2. Oppositely regulated genes in adipose tissue after 7 wk supplementation with n-3 (omega-3) and n-6 (omega-6). Rank Product Analysis was used to identify significantly differentially expressed genes in adipose tissue subjected to microarray analysis. Oppositely regulated genes after n-3 and n-6 supplementation with q-value > 0.1 and log2FC change ≥ 0.1 or ≤ −0.1 are presented.

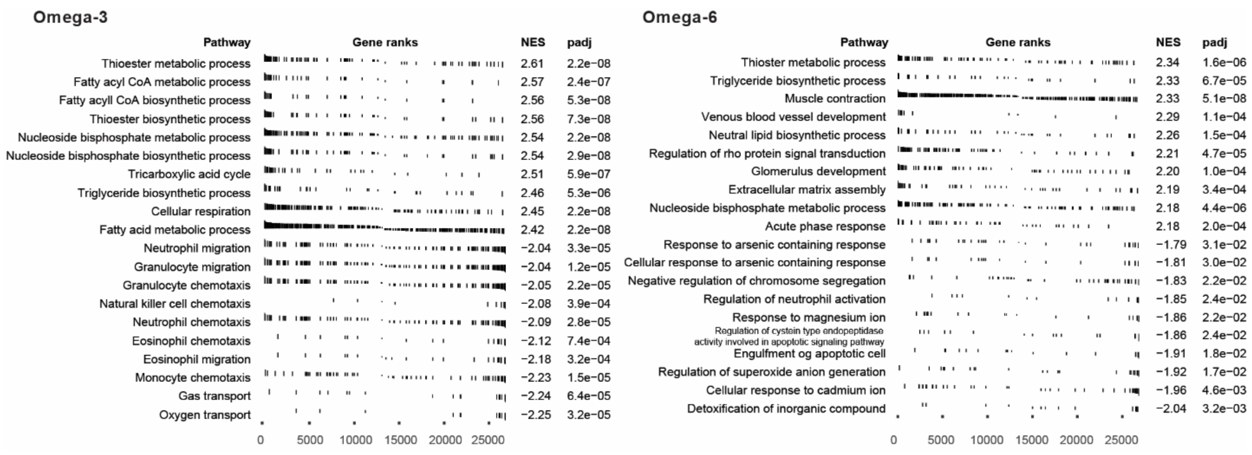

Supplemental Figure 3. Gene set enrichment analysis (GSEA) showing enriched gene ontology categories for up- and down-regulated adipose genes after 7 wk supplementation with n-3 (omega-3) or n-6 (omega-6). Differentially expressed genes were identified following analysis of microarray data by Rank Product Analysis, and subjected to GSEA using gene ontology categories from the molecular signatures database provided by the BROAD institute, Abbreviations: NES, normalized enrichment score; padj; adjusted p-value.

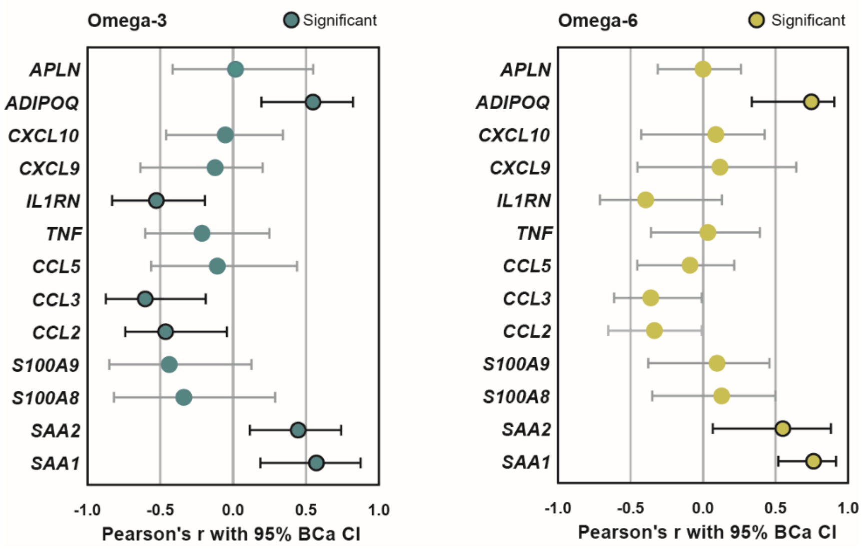

Supplemental Figure 4. Correlation of changes in adipose mRNA expression of inflammatory markers and PPARγ2 mRNA after 7 wk supplementation with n-3 (omega-3) or n-6 (omega-6), Gene expression was measured in abdominal subcutaneous adipose tissue by qPCR, Correlation coefficients (95% BCa Cls) were obtained by bootstrapped Pearson's correlation analyses using fold change values, Data were log_2 transformed before analyses, Abbreviations: BCa CI, bias-corrected and accelerated confidence interval

Supplemental Tables

Supplemental Table 1. Primers used for qPCR

Gene	Primer	Sequence 5’ to 3’
APLN	Forward	aagggacccatgcctttctg
	Reverse	tgcgaggtgagagctgaatg
ADIPOQ	Forward	agtggatctgacgacaccaaa
	Reverse	tgactgggcaggattaagagg
PPARG2	Forward	ccatgctgttatgggtgaa
	Reverse	tgtgtcaaccatggtcatttc
CXCL9	Forward	gtggtgttcttttcctcttggg
	Reverse	ctcactactggggttccttgc
CXCL10	Forward	gaaagcagttagcaaggaaaggt
	Reverse	gacatatactccatgtagggaagtga
S100A8	Forward	cgtctggttcaaagagttgga
	Reverse	gctgccacgcccatc
S100A9	Forward	acgcaacatagagaccatcatc
	Reverse	ccttttcattcttattctccttcttg
SAA1	Forward	ctgcagaagtgatcagcg
	Reverse	attgtgtacccttcccc
SAA2	Forward	ttctgctccttggtcctga
	Reverse	agagtaggctctccacatgtcc
IL1RN	Forward	atgtgcctgtcctgtgtcaa
	Reverse	gctttctgttctcgctcagg
CCL2	Forward	agtctctgccgcccttct
	Reverse	gtgactggggcattgattg
CCL3	Forward	gagcccacattccgtcac
	Reverse	gcgtgtcagcagcaagtg
CCL5	Forward	cgctgtcatcctcattgcta
	Reverse	ggtgtggtgtccgaggaata
TNF	Forward	cagcctcttctccttcctgat
	Reverse	gccagagggctgattagaga

Supplemental Table 2. Relative changes in fatty acid levels in RBCMs after seven wk of supplementation with n-3 or n-6 PUFAs[1]

Variable and treatment	Baseline (n = 39)[2]	Follow-up (n =38)[2]	Relative change[3]	Time[4]	Tx × time[5]	CV (%)[6]
**Total fatty acids**						
**PUFA n-3, wt%**					<0.001	0.49
n-3	11.9 (10.3, 13.7)	16.8 (15.0, 18.7)	33.2 (29.5, 37.0)	<0.001		
n-6	12.7 (11.1, 14.6)	11.6 (10.4, 13.1)	-4.28 (-5.78, -2.76)	<0.001		
**n-3 index, wt%**					<0.001	0.48
n-3	8.48 (7.04, 10.2)	12.5 (11.0, 14.3)	40.6 (35.8, 45.6)	<0.001		
n-6	9.04 (7.69, 10.6)	8.33 (7.19, 9.65)	-3.82 (-5.64, -1.96)	<0.001		
**PUFA n-6, wt%**					<0.001	0.23
n-3	28.8 (26.8, 30.9)	24.4 (22.7, 26.1)	-12.5 (-13.7, -11.3)	<0.001		
n-6	27.9 (26.0, 29.9)	29.9 (28.3, 31.5)	4.41 (3.63, 5.19)	<0.001		
**PUFA n-9, wt%**					<0.001	1.54
n-3	0.13 (0.11, 0.15)	0.11 (0.095, 0.12)	-18.2 (-21.3, -14.9)	<0.001		
n-6	0.13 (0.11, 0.15)	0.11 (0.099, 0.13)	-13.6 (-15.6, -11.6)	<0.001		
**MUFA, wt%**					<0.001	0.27
n-3	18.5 (17.5, 19.6)	17.9 (17.1, 18.7)	-3.10 (-3.98, -2.21)	<0.001		
n-6	18.5 (17.6, 19.3)	17.7 (16.8, 18.6)	-4.42 (-5.28, -3.54)	<0.001		
**SFA, wt%**					<0.001	0.15
n-3	40.2 (39.6, 40.8)	40.5 (39.9, 41.0)	0.45 (0.24, 0.66)	<0.001		
n-6	40.3 (39.6, 41.0)	40.3 (39.7, 40.9)	0.053 (-0.16, 0.26)	0.616		
**Trans FA, wt%**					0.783	1.19
n-3	0.25 (0.20, 0.30)	0.24 (0.20, 0.29)	-3.71 (-6.64, -0.69)	0.017		
n-6	0.25 (0.20, 0.30)	0.24 (0.20, 0.29)	-3.33 (-6.27, -0.30)	0.032		
**Omega-3 (n-3)**						
**C18:3 n-3, ALA, wt%**					0.129	1.66
n-3	0.18 (0.14, 0.22)	0.15 (0.12, 0.19)	-17.4 (-24.0, -10.2)	<0.001		
n-6	0.19 (0.15, 0.23)	0.14 (0.12, 0.17)	-21.8 (-25.9, -17.5)	<0.001		
**C20:4 n-3, ETA, wt%**					<0.001	1.76
n-3	0.14 (0.12, 0.18)	0.14 (0.12, 0.17)	-1.49 (-6.41, 3.68)	0.561		
n-6	0.15 (0.12, 0.18)	0.12 (0.10, 0.14)	-18.2 (-22.3, -13.9)	<0.001		

**C20:5 n-3, EPA, wt%**					<0.001	0.16
n-3	1.33 (0.91, 1.94)	4.14 (3.26, 5.26)	157 (134, 182)	<0.001		
n-6	1.66 (1.19, 2.31)	1.22 (0.90, 1.63)	-10.2 (-15.8, -4.37)	0.001		
**C22:5 n-3, DPA, wt%**					<0.001	0.60
n-3	3.00 (2.74, 3.29)	3.88 (3.61, 4.18)	18.6 (16.2, 21.1)	<0.001		
n-6	3.30 (2.92, 3.72)	3.03 (2.78, 3.30)	-3.78 (-4.98, -2.55)	<0.001		
**C22:6 n-3, DHA, wt%**					<0.001	0.58
n-3	7.11 (6.08, 8.32)	8.36 (7.56, 9.24)	15.0 (12.1, 18.1)	<0.001		
n-6	7.34 (6.42, 8.39)	7.09 (6.21, 8.09)	-1.59 (-2.97, -0.19)	0.027		
**Omega-6 (n-6)**						
**C18:2 n-6, LA, wt%**					<0.001	0.35
n-3	8.74 (8.06, 9.49)	7.21 (6.45, 8.06)	-18.2 (-20.2, -16.3)	<0.001		
n-6	8.69 (7.92, 9.54)	9.90 (9.14, 10.7)	12.5 (9.87, 15.2)	<0.001		
**C18:3 n-6, GLA, wt %**					<0.001	3.26
n-3	0.050 (0.037, 0.068)	0.029 (0.021, 0.040)	-42.3 (-49.0, -34.8)	<0.001		
n-6	0.049 (0.036, 0.066)	0.051 (0.038, 0.068)	1.90 (-5.65, 10.1)	0.629		
**C20:2 n-6, Eicosadienoic acid, wt%**					<0.001	0.33
n-3	0.21 (0.19, 0.24)	0.18 (0.16, 0.20)	-13.3 (-15.6, -10.9)	<0.001		
n-6	0.20 (0.18, 0.23)	0.23 (0.21, 0.26)	13.1 (10.8, 15.4)	<0.001		
**C20:3 n-6, DGLA, wt%,**					<0.001	0.40
n-3	1.59 (1.35, 1.87)	1.16 (0.97, 1.38)	-26.6 (-29.8, -23.2)	<0.001		
n-6	1.56 (1.33, 1.84)	1.60 (1.36, 1.87)	1.26 (-1.04, 3.61)	0.282		
**C20:4 n-6, ARA, wt%**					<0.001	0.26
n-3	15.3 (13.8, 16.9)	13.5 (12.3, 14.8)	-10.0 (-11.4, -8.61)	<0.001		
n-6	14.8 (13.3, 16.3)	15.2 (13.9, 16.5)	-0.40 (-1.92, 1.15)	0.610		
**C22:2 n-6, Docosadienoic acid, wt%**					<0.001	2.67
n-3	0.041 (0.033, 0.051)	0.035 (0.027, 0.045)	-12.1 (-16.8, -7.11)	<0.001		
n-6	0.037 (0.028, 0.048)	0.048 (0.039, 0.059)	21.6 (15.1, 28.4)	<0.001		
**C22:4 n-6, Adrenic acid, wt%**					<0.001	0.53
n-3	2.42 (1.87, 3.12)	1.87 (1.49, 2.35)	-13.4 (-15.7, -11.1)	<0.001		
n-6	2.16 (1.67, 2.80)	2.44 (1.94, 3.06)	5.78 (3.86, 7.72)	<0.001		

**C22:5 n-6, Docosapentaenoic acid, wt%**					<0.001	0.63
n-3	0.33 (0.26, 0.43)	0.27 (0.23, 0.33)	-13.3 (-15.8, -10.8)	<0.001		
n-6	0.30 (0.24, 0.38)	0.32 (0.26, 0.40)	0.33 (-1.77, 2.48)	0.756		
**Omega-7 (n-7)**						
**C16:1 n-7, Palmitoleic acid, wt%**					0.089	0.65
n-3	0.26 (0.18, 0.38)	0.21 (0.15, 0.29)	-21.2 (-29.2, -12.3)	<0.001		
n-6	0.27 (0.19, 0.37)	0.22 (0.17, 0.30)	-15.5 (-20.6, -10.1)	<0.001		
**C18:1 n-7, Cis-vaccenic acid, wt%**					0.020	0.29
n-3	0.97 (0.89, 1.06)	0.95 (0.87, 1.03)	-2.00 (-3.54, -0.44)	0.013		
n-6	0.96 (0.89, 1.04)	0.93 (0.85, 1.01)	-3.94 (-5.45, -2.41)	<0.001		
**C20:1 n-7, Paullinic acid, wt%**					<0.001	3.03
n-3	0.022 (0.018, 0.028)	0.026 (0.021, 0.032)	14.7 (6.79, 23.3)	<0.001		
n-6	0.023 (0.019, 0.028)	0.022 (0.018, 0.026)	-1.73 (-6.51, 3.30)	0.489		
**C22:1 n-7, Cis-15-docosenoic acid, wt%**					<0.001	1.11
n-3	0.017 (0.012, 0.024)	0.021 (0.018, 0.025)	23.4 (12.7, 35.1)	<0.001		
n-6	0.018 (0.013, 0.026)	0.017 (0.012, 0.024)	-2.22 (-10.7, 7.05)	0.624		
**Omega-9 (n-9)**						
**C16:1 n-9, Cis-7-hexadecenoic acid, wt%**					0.824	1.03
n-3	0.043 (0.034, 0.054)	0.038 (0.032, 0.045)	-8.67 (-13.4, -3.63)	0.001		
n-6	0.041 (0.033, 0.051)	0.038 (0.031, 0.047)	-8.10 (-12.9, -3.04)	0.002		
**C18:1 n-9, Oleic acid, wt%**					<0.001	0.33
n-3	11.9 (11.2, 12.7)	11.4 (10.9, 12.0)	-3.67 (-4.81, -2.51)	<0.001		
n-6	11.9 (11.3, 12.6)	11.2 (10.6, 11.9)	-5.57 (-6.69, -4.43)	<0.001		
**C20:1 n-9, Gondoic acid, wt%**					<0.001	0.26
n-3	0.27 (0.23, 0.31)	0.24 (0.21, 0.28)	-8.90 (-11.0, -6.71)	<0.001		
n-6	0.26 (0.22, 0.31)	0.26 (0.23, 0.30)	-2.33 (-4.63, 0.016)	0.052		
**C20:3 n-9, Mead acid, wt%**					<0.001	1.54
n-3	0.13 (0.11, 0.15)	0.11 (0.095, 0.12)	-18.2 (-21.3, -14.9)	<0.001		
n-6	0.13 (0.11, 0.15)	0.11 (0.099, 0.13)	-13.6 (-15.6, -11.6)	<0.001		
**C22:1 n-9, Erucic acid, wt%**					0.839	6.33
n-3	0.076 (0.063, 0.092)	0.073 (0.061, 0.087)	-9.13 (-14.3, -3.66)	0.002		

n-6	0.077 (0.060, 0.099)	0.074 (0.062, 0.088)	-9.63 (-14.8, -4.20)	0.001		
**C24:1 n-9, Nervonic, wt%**					0.329	1.43
n-3	4.69 (4.24, 5.18)	4.62 (4.28, 4.99)	-0.16 (-1.51, 1.20)	0.810		
n-6	4.60 (4.24, 5.00)	4.60 (4.23, 5.00)	-0.78 (-2.12, 0.58)	0.257		
**Omega 11 (n-11)**						
**C20:1n-11, Gadoleic acid, wt%**					0.012	2.93
n-3	0.033 (0.027, 0.041)	0.032 (0.027, 0.039)	-0.21 (-5.11, 4.95)	0.936		
n-6	0.032 (0.026, 0.039)	0.029 (0.023, 0.036)	-6.74 (-11.3, -1.92)	0.007		
**Saturated fatty acids**						
**C14:0, Myristic acid, wt%**					0.179	1.11
n-3	0.29 (0.24, 0.34)	0.29 (0.24, 0.34)	-3.51 (-9.17, 2.51)	0.245		
n-6	0.30 (0.26, 0.36)	0.27 (0.24, 0.32)	-7.64 (-13.1, -1.88)	0.010		
**C15:0, Pentadecanoic acid, wt%**					0.406	0.79
n-3	0.12 (0.10, 0.14)	0.12 (0.10, 0.14)	-1.84 (-4.80, 1.22)	0.234		
n-6	0.12 (0.10, 0.14)	0.12 (0.10, 0.14)	-2.95 (-5.88, 0.075)	0.056		
**C16:0, Palmitic acid, wt%**					0.001	0.30
n-3	18.0 (17.4, 18.6)	18.0 (17.5, 18.6)	-0.14 (-0.86, 0.59)	0.705		
n-6	18.0 (17.5, 18.6)	17.8 (17.3, 18.3)	-1.14 (-1.85, -0.42)	0.002		
**C17:0, Heptadecanoic acid, wt%**					<0.001	0.50
n-3	0.28 (0.24, 0.32)	0.29 (0.26, 0.32)	3.37 (1.81, 4.95)	<0.001		
n-6	0.28 (0.24, 0.32)	0.28 (0.24, 0.32)	-0.25 (-1.75, 1.28)	0.747		
**C18:0, Stearic acid, wt%**					0.024	0.28
n-3	15.0 (14.5, 15.4)	15.1 (14.7, 15.5)	0.60 (-0.076, 1.28)	0.081		
n-6	15.0 (14.6, 15.4)	15.2 (14.8, 15.6)	1.21 (0.53, 1.89)	0.001		
**C20:0, Arachidic acid, wt%**					0.098	0.63
n-3	0.40 (0.36, 0.44)	0.41 (0.37, 0.45)	1.99 (0.61, 3.39)	0.005		
n-6	0.40 (0.37, 0.44)	0.40 (0.37, 0.44)	0.93 (-0.44, 2.32)	0.182		
**C22:0, Behenic acid, wt%**					0.239	1.24
n-3	1.48 (1.34, 1.64)	1.49 (1.36, 1.64)	0.15 (-1.08, 1.40)	0.810		
n-6	1.49 (1.36, 1.64)	1.50 (1.38, 1.63)	0.77 (-0.47, 2.03)	0.224		
**C23:0, Tricosylic acid, wt%**					0.023	1.16

n-3	0.23 (0.21, 0.26)	0.24 (0.21, 0.28)	2.52 (0.95, 4.11)	0.002		
n-6	0.24 (0.21, 0.27)	0.24 (0.21, 0.27)	0.76 (-0.78, 2.32)	0.333		
**C24:0, Lignoceric acid, wt%**					0.227	1.56
n-3	4.04 (3.68, 4.43)	4.16 (3.77, 4.59)	2.62 (1.23, 4.03)	<0.001		
n-6	4.06 (3.71, 4.43)	4.13 (3.77, 4.53)	1.92 (0.54, 3.32)	0.007		

[1] Pooled data of fasting fatty acid levels in RBCMs (measured as wt%) were analyzed with cLMM adjusted for the main effect of period and subject-averaged baselines, Values were transformed by the natural logarithm before the analyses, The n-3 index is the total sum of EPA and DHA measured in RBCMs (wt%).
[2] Values are geometric means (1 SD ranges) of fasting levels at baseline and follow-up.
[3] Relative changes from baseline to follow-up as percentages (95% CIs) calculated from cLMM estimates: % = $(\exp^{estimate} - 1) \times 100$,
[4] P-values (from cLMMs) for relative changes from baseline to follow-up within treatments (time effects).
[5] P-values (from cLMMs) for relative changes from baseline to follow-up between treatments (group differences in time effects).
[6] CV was determined by using a random baseline sample from one participant (5 replicates),
Abbreviations: ALA, alpha-linolenic acid; ARA, arachidonic acid; cLMM, constrained linear-mixed effects model; CV, coefficient of variation; DHA, docosahexaenoic acid; DGLA, dihomo-gamma-linolenic acid; DPA, docosapentaenoic acid; EPA, eicosapentaenoic acid; ETA, eicosatetraenoic acid, GLA, gamma-linolenic acid; n-3, omega-3 PUFAs; n-6, omega-6 PUFAs; PUFAs, polyunsaturated fatty acids; RBCMs, red blood cell membranes; Tx, treatment; wt%, weight percentage of total fatty acid

Supplemental Table 3. Relative changes in fatty acid levels in serum before and after seven wk of supplementation with n-3 or n-6 PUFAs[1]

Variable and treatment	Baseline (n = 39)[2]	Follow-up (n =38)[2]	Relative change[3]	Time[4]	Tx × time[5]
**Total fatty acids**					
**PUFA n-3, wt%**					<0.001
n-3	7.01 (5.44, 9.03)	15.0 (12.3, 18.4)	116 (96.8, 136)	<0.001	
n-6	7.15 (5.84, 8.74)	6.16 (4.94, 7.68)	-11.1 (-16.2, -5.59)	<0.001	
**PUFA n-6, wt%**					<0.001
n-3	35.4 (32.3, 38.9)	30.8 (27.5, 34.5)	-13.0 (-15.5, -10.5)	<0.001	
n-6	35.2 (31.7, 39.2)	39.2 (36.4, 42.2)	10.6 (7.45, 13.9)	<0.001	
**PUFA n-9, wt%**					0.915
n-3	0.13 (0.10, 0.17)	0.11 (0.081, 0.14)	-20.1 (-26.9, -12.6)	<0.001	
n-6	0.14 (0.10, 0.18)	0.11 (0.077, 0.15)	-20.4 (-27.2, -12.9)	<0.001	

**MUFA, wt%**					0.023
n-3	25.9 (23.5, 28.4)	22.6 (20.5, 24.8)	-11.9 (-14.6, -9.12)	<0.001	
n-6	25.5 (22.8, 28.5)	23.4 (21.2, 25.7)	-8.87 (-11.7, -5.97)	<0.001	
**SFA, wt%**					0.799
n-3	30.7 (29.4, 32.2)	30.6 (29.2, 32.1)	-1.09 (-2.77, 0.61)	0.204	
n-6	31.1 (29.4, 32.9)	30.5 (29.2, 31.9)	-1.32 (-2.99, 0.38)	0.126	
**Trans FA, wt%**					0.999
n-3	0.27 (0.21, 0.35)	0.24 (0.19, 0.31)	-9.29 (-17.1, -0.73)	0.034	
n-6	0.26 (0.20, 0.35)	0.24 (0.19, 0.31)	-9.28 (-17.1, -0.73)	0.034	
**Omega-3 (n-3)**					
**C18:3 n-3, ALA, wt%**					0.010
n-3	0.85 (0.62, 1.15)	0.75 (0.56, 0.99)	-11.4 (-18.7, -3.46)	0.006	
n-6	0.85 (0.64, 1.14)	0.67 (0.52, 0.87)	-20.4 (-26.9, -13.2)	<0.001	
**C20:4 n-3, ETA, wt%**					<0.001
n-3	0.17 (0.13, 0.24)	0.19 (0.15, 0.25)	4.30 (-6.33, 16.1)	0.439	
n-6	0.19 (0.14, 0.26)	0.13 (0.10, 0.17)	-29.2 (-36.4, -21.2)	<0.001	
**C20:5 n-3, EPA, wt%**					<0.001
n-3	1.50 (0.97, 2.33)	6.78 (4.91, 9.37)	349 (281, 429)	<0.001	
n-6	1.61 (1.10, 2.37)	1.25 (0.84, 1.84)	-14.5 (-24.4, -3.37)	0.013	
**C21:5 n-3, Heneicosapentaenoic acid, wt%**					<0.001
n-3	0.012 (0.007, 0.021)	0.062 (0.039, 0.097)	411 (297, 558)	<0.001	
n-6	0.013 (0.007, 0.023)	0.011 (0.007, 0.018)	-3.48 (-17.6, 13.0)	0.657	
**C22:5 n-3, DPA, wt%**					<0.001
n-3	0.72 (0.64, 0.82)	1.23 (1.03, 1.46)	68.7 (56.9, 81.4)	<0.001	
n-6	0.74 (0.63, 0.86)	0.66 (0.56, 0.77)	-9.51 (-13.5, -5.29)	<0.001	
**C22:6 n-3, DHA, wt%**					<0.001
n-3	3.60 (2.74, 4.72)	5.78 (4.87, 6.87)	61.9 (51.0, 73.5)	<0.001	
n-6	3.57 (2.86, 4.47)	3.33 (2.60, 4.26)	-6.79 (-13.0, -0.087)	0.047	
**Omega-6 (n-6)**					
**C18:2 n-6, LA, wt%**					<0.001
n-3	26.0 (23.3, 29.1)	22.5 (19.3, 26.2)	-13.5 (-16.7, -10.1)	<0.001	

n-6	25.9 (22.7, 29.4)	29.9 (27.2, 32.9)	15.1 (10.8, 19.5)	<0.001	
**C18:3 n-6, GLA, wt %**					<0.001
n-3	0.33 (0.25, 0.44)	0.20 (0.13, 0.29)	-41.7 (-48.9, -33.5)	<0.001	
n-6	0.34 (0.25, 0.47)	0.38 (0.27, 0.54)	12.2 (3.52, 21.6)	0.005	
**C20:2 n-6, Eicosadienoic acid, wt%**					<0.001
n-3	0.20 (0.17, 0.23)	0.16 (0.14, 0.18)	-18.5 (-21.8, -15.0)	<0.001	
n-6	0.20 (0.17, 0.22)	0.22 (0.19, 0.25)	8.84 (4.37, 13.5)	<0.001	
**C20:3 n-6, DGLA, wt%,**					<0.001
n-3	1.67 (1.43, 1.95)	1.05 (0.80, 1.39)	-37.6 (-43.3, -31.3)	<0.001	
n-6	1.69 (1.41, 2.04)	1.66 (1.36, 2.02)	-1.50 (-6.21, 3.45)	0.543	
**C20:4 n-6, ARA, wt%**					0.802
n-3	6.68 (5.29, 8.43)	6.48 (5.50, 7.63)	-2.72 (-6.56, 1.29)	0.179	
n-6	6.66 (5.47, 8.09)	6.53 (5.36, 7.94)	-2.22 (-5.06, 0.70)	0.133	
**C22:2 n-6, Docosadienoic acid, wt%**					<0.001
n-3	0.013 (0.010, 0.018)	0.011 (0.008, 0.015)	-17.8 (-24.9, -9.96)	<0.001	
n-6	0.013 (0.010, 0.017)	0.016 (0.013, 0.020)	17.1 (9.83, 24.8)	<0.001	
**C22:4 n-6, Adrenic acid, wt%**					<0.001
n-3	0.17 (0.13, 0.22)	0.11 (0.085, 0.14)	-34.9 (-39.8, -29.6)	<0.001	
n-6	0.17 (0.13, 0.21)	0.16 (0.12, 0.21)	-3.60 (-7.92, 0.92)	0.115	
**C22:5 n-6, Docosapentaenoic acid, wt%**					0.192
n-3	0.11 (0.082, 0.14)	0.099 (0.079, 0.12)	-8.56 (-16.7, 0.38)	0.060	
n-6	0.11 (0.082, 0.14)	0.094 (0.071, 0.12)	-13.5 (-18.0, -8.71)	<0.001	
**Omega-7 (n-7)**					
**C16:1 n-7, Palmitoleic acid, wt%**					0.245
n-3	1.58 (1.13, 2.21)	1.28 (0.91, 1.81)	-18.7 (-25.8, -10.9)	<0.001	
n-6	1.57 (1.12, 2.18)	1.35 (0.98, 1.88)	-14.1 (-21.6, -5.86)	0.001	
**C18:1 n-7, Cis-vaccenic acid, wt%**					0.223
n-3	1.52 (1.35, 1.70)	1.36 (1.21, 1.54)	-8.70 (-11.9, -5.33)	<0.001	
n-6	1.47 (1.30, 1.66)	1.39 (1.25, 1.56)	-6.71 (-10.0, -3.28)	<0.001	
**C20:1 n-7, Paullinic acid, wt%**					<0.001
n-3	0.013 (0.011, 0.015)	0.015 (0.013, 0.018)	16.8 (9.11, 25.1)	<0.001	

n-6	0.013 (0.011, 0.016)	0.013 (0.011, 0.015)	0.20 (-3.75, 4.32)	0.920	
**C22:1 n-7, Cis-15-docosenoic acid, wt%**					<0.001
n-3	0.004 (0.003, 0.006)	0.006 (0.004, 0.008)	53.0 (36.9, 71.0)	<0.001	
n-6	0.004 (0.003, 0.006)	0.003 (0.002, 0.004)	-5.81 (-15.7, 5.26)	0.288	
**Omega-9 (n-9)**					
**C16:1 n-9, Cis-7-hexadecenoic acid, wt%**					<0.001
n-3	0.26 (0.22, 0.32)	0.24 (0.19, 0.30)	-12.3 (-17.2, -7.21)	<0.001	
n-6	0.25 (0.20, 0.31)	0.26 (0.21, 0.31)	-2.33 (-6.64, 2.18)	0.303	
**C18:1 n-9, Oleic acid, wt%**					0.016
n-3	20.7 (18.5, 23.1)	17.9 (16.1, 19.9)	-12.7 (-15.7, -9.63)	<0.001	
n-6	20.5 (18.0, 23.2)	18.6 (16.7, 20.8)	-9.14 (-12.2, -5.95)	<0.001	
**C20:1 n-9, Gondoic acid, wt%**					0.185
n-3	0.17 (0.14, 0.20)	0.15 (0.12, 0.18)	-12.7 (-18.2, -6.74)	<0.001	
n-6	0.17 (0.14, 0.20)	0.16 (0.13, 0.19)	-8.68 (-14.5, -2.48)	0.007	
**C20:3 n-9, Mead acid, wt%**					0.915
n-3	0.13 (0.10, 0.17)	0.11 (0.081, 0.14)	-20.1 (-26.9, -12.6)	<0.001	
n-6	0.14 (0.10, 0.18)	0.11 (0.077, 0.15)	-20.4 (-27.2, -12.9)	<0.001	
**C22:1 n-9, Erucic acid, wt%**					0.329
n-3	0.028 (0.024, 0.032)	0.026 (0.022, 0.031)	-10.2 (-16.9, -3.04)	0.006	
n-6	0.029 (0.024, 0.035)	0.027 (0.022, 0.033)	-6.58 (-13.5, 0.91)	0.083	
**C24:1 n-9, Nervonic, wt%**					0.068
n-3	1.14 (0.91, 1.44)	1.20 (0.98, 1.46)	5.36 (-0.35, 11.4)	0.066	
n-6	1.12 (0.88, 1.42)	1.13 (0.92, 1.39)	-0.13 (-5.54, 5.59)	0.963	
**Omega 11 (n-11)**					
**C20:1 n-11, Gadoleic acid, wt%**					<0.001
n-3	0.040 (0.030, 0.054)	0.040 (0.031, 0.052)	-1.96 (-8.97, 5.59)	0.598	
n-6	0.040 (0.030, 0.053)	0.034 (0.027, 0.043)	-14.2 (-20.0, -8.06)	<0.001	
**Saturated fatty acids**					
**C14:0, Myristic acid, wt%**					0.720
n-3	0.84 (0.65, 1.08)	0.81 (0.61, 1.07)	-7.51 (-16.1, 1.91)	0.113	
n-6	0.91 (0.66, 1.26)	0.82 (0.64, 1.06)	-5.72 (-14.4, 3.88)	0.231	

**C15:0, Pentadecanoic acid, wt%**					0.961
n-3	0.20 (0.16, 0.23)	0.18 (0.16, 0.22)	-5.93 (-10.8, -0.75)	0.026	
n-6	0.20 (0.16, 0.23)	0.18 (0.15, 0.22)	-6.05 (-10.9, -0.88)	0.023	
**C16:0, Palmitic acid, wt%**					0.581
n-3	20.1 (18.7, 21.5)	19.5 (18.1, 21.0)	-2.98 (-5.26, -0.65)	0.013	
n-6	20.2 (18.7, 21.8)	19.7 (18.4, 21.0)	-2.28 (-4.58, 0.069)	0.057	
**C17:0, Heptadecanoic acid, wt%**					0.236
n-3	0.26 (0.22, 0.30)	0.26 (0.22, 0.29)	0.71 (-2.88, 4.44)	0.699	
n-6	0.25 (0.22, 0.29)	0.25 (0.22, 0.29)	-1.28 (-4.80, 2.37)	0.483	
**C18:0, Stearic acid, wt%**					0.127
n-3	7.04 (6.58, 7.53)	7.41 (6.93, 7.93)	4.33 (1.94, 6.77)	<0.001	
n-6	7.16 (6.67, 7.68)	7.28 (6.73, 7.86)	2.36 (0.022, 4.76)	0.048	
**C20:0, Arachidic acid, wt%**					0.069
n-3	0.22 (0.18, 0.26)	0.23 (0.20, 0.26)	4.33 (0.23, 8.59)	0.038	
n-6	0.22 (0.19, 0.27)	0.22 (0.19, 0.26)	0.31 (-3.63, 4.41)	0.879	
**C22:0, Behenic acid, wt%**					0.157
n-3	0.64 (0.52, 0.78)	0.68 (0.58, 0.79)	4.58 (-0.45, 9.87)	0.074	
n-6	0.65 (0.53, 0.80)	0.65 (0.54, 0.79)	0.62 (-4.22, 5.71)	0.804	
**C23:0, Tricosylic acid, wt%**					0.055
n-3	0.26 (0.20, 0.33)	0.27 (0.22, 0.33)	4.20 (0.17, 8.39)	0.041	
n-6	0.26 (0.20, 0.33)	0.26 (0.20, 0.33)	-0.48 (-5.27, 4.55)	0.846	
**C24:0, Lignoceric acid, wt%**					0.169
n-3	0.56 (0.45, 0.69)	0.60 (0.51, 0.70)	5.22 (0.13, 10.6)	0.045	
n-6	0.57 (0.46, 0.71)	0.58 (0.47, 0.70)	1.31 (-3.59, 6.46)	0.604	

[1] Pooled data of fasting serum fatty acid levels (measured as wt%) were analyzed with cLMM adjusted for the main effect of period and subject-averaged baselines, Values were transformed by the natural logarithm before the analyses,

[2] Values are geometric means (1 SD ranges) of fasting levels at baseline and follow-up.

[3] Relative changes from baseline to follow-up as percentages (95% CIs) calculated from cLMM estimates: % = $(\exp^{\text{estimate}} - 1) \times 100$,

[4] P-values (from cLMMs) for relative changes from baseline to follow-up within treatments (time effects).

[5] P-values (from cLMMs) for relative changes from baseline to follow-up between treatments (group differences in time effects).

Abbreviations: ALA, alpha-linolenic acid; ARA, arachidonic acid; cLMM, constrained linear-mixed effects model; DHA, docosahexaenoic acid; DGLA, dihomo-gamma-linolenic acid; DPA, docosapentaenoic acid; EPA, eicosapentaenoic acid; ETA, eicosatetraenoic acid, GLA, gamma-linolenic acid; n-3, omega-3 PUFAs; n-6, omega-6 PUFAs; PUFAs, polyunsaturated fatty acids; Tx, treatment; wt%, weight percentage of total fatty acids.

Supplemental Table 4. Correlations between fasting fatty acid levels in serum and RBCMs[1]

	Baseline[2]		Follow-up[3]		Relative change[3]	
**Variable and group**	**Pearson's *r***	**P-value**	**Pearson's *r***	**P-value**	**Pearson's *r***	**P-value**
**n-3 index, wt%**	0.75 (0.49, 0.88)	<0.001	0.93 (0.88, 0.95)	<0.001	0.96 (0.93, 0.97)	<0.001
**n-3**	0.88 (0.72, 0.94)	<0.001	0.82 (0.70, 0.89)	<0.001	0.88 (0.76, 0.93)	<0.001
**n-6**	0.64 (0.28, 0.82)	<0.001	0.72 (0.44, 0.85)	<0.001	0.56 (0.30, 0.74)	<0.001
**ALA, wt%**	0.85 (0.74, 0.91)	<0.001	0.80 (0.70, 0.87)	<0.001	0.77 (0.65, 0.84)	<0.001
**n-3**	0.84 (0.72, 0.90)	<0.001	0.80 (0.64, 0.89)	<0.001	0.82 (0.68, 0.90)	<0.001
**n-6**	0.88 (0.80, 0.92)	<0.001	0.80 (0.65, 0.89)	<0.001	0.66 (0.45, 0.79)	<0.001
**EPA, wt%**	0.73 (0.51, 0.87)	<0.001	0.97 (0.95, 0.98)	<0.001	0.96 (0.94, 0.98)	<0.001
**n-3**	0.89 (0.76, 0.95)	<0.001	0.88 (0.77, 0.95)	<0.001	0.89 (0.73, 0.94)	<0.001
**n-6**	0.61 (0.31, 0.80)	<0.001	0.79 (0.52, 0.90)	<0.001	0.69 (0.46, 0.81)	<0.001
**DPA, wt%**	0.48 (0.22, 0.67)	0.003	0.84 (0.78, 0.88)	<0.001	0.92 (0.89, 0.95)	<0.001
**n-3**	0.48 (0.19, 0.67)	0.003	0.46 (0.20, 0.65)	0.004	0.74 (0.56, 0.87)	<0.001
**n-6**	0.35 (0.038, 0.61)	0.035	0.29 (-0.051, 0.53)	0.078	0.47 (0.14, 0.69)	0.004
**DHA, wt%**	0.80 (0.59, 0.91)	<0.001	0.82 (0.71, 0.88)	<0.001	0.90 (0.85, 0.93)	<0.001
**n-3**	0.86 (0.68, 0.93)	<0.001	0.74 (0.60, 0.82)	<0.001	0.83 (0.69, 0.90)	<0.001
**n-6**	0.68 (0.37, 0.85)	<0.001	0.72 (0.45, 0.86)	<0.001	0.48 (0.21, 0.69)	0.003
**LA, wt%**	0.54 (0.21, 0.78)	0.001	0.90 (0.85, 0.93)	<0.001	0.89 (0.84, 0.92)	<0.001
**n-3**	0.45 (0.12, 0.71)	0.005	0.79 (0.64, 0.88)	<0.001	0.70 (0.46, 0.82)	<0.001
**n-6**	0.61 (0.33, 0.78)	<0.001	0.68 (0.47, 0.78)	<0.001	0.59 (0.37, 0.74)	<0.001
**GLA, wt%**	0.80 (0.55, 0.91)	<0.001	0.92 (0.89, 0.95)	<0.001	0.91 (0.85, 0.94)	<0.001
**n-3**	0.81 (0.66, 0.91)	<0.001	0.84 (0.67, 0.91)	<0.001	0.82 (0.60, 0.93)	<0.001
**n-6**	0.82 (0.61, 0.93)	<0.001	0.89 (0.78, 0.94)	<0.001	0.81 (0.67, 0.89)	<0.001
**DGLA, wt%**	0.81 (0.66, 0.89)	<0.001	0.88 (0.80, 0.92)	<0.001	0.92 (0.88, 0.95)	<0.001
**n-3**	0.79 (0.61, 0.89)	<0.001	0.80 (0.55, 0.89)	<0.001	0.85 (0.70, 0.92)	<0.001
**n-6**	0.82 (0.68, 0.90)	<0.001	0.72 (0.44, 0.85)	<0.001	0.84 (0.66, 0.93)	<0.001
**ARA, wt%**	0.75 (0.51, 0.87)	<0.001	0.59 (0.40, 0.73)	<0.001	0.39 (0.16, 0.58)	0.001
**n-3**	0.74 (0.51, 0.86)	<0.001	0.69 (0.42, 0.83)	<0.001	0.63 (0.47, 0.78)	<0.001
**n-6**	0.70 (0.47, 0.83)	<0.001	0.71 (0.42, 0.86)	<0.001	0.61 (0.41, 0.78)	<0.001

[1] Values are Pearson's correlation coefficients (95% BCa CIs) from bivariate correlation analyses using fasting fatty acid levels (measured as wt%) in serum and RBCMs, Data were log-transformed before the analyses, The n-6/n-3 ratio was calculated from the total levels of n-6 and n-3 PUFAs, The n-3 index is the total sum of EPA and DHA measured in RBCMs (wt%),

[2] Correlations are shown for all participants at the first baseline visit (B1) before any intervention (grey cells) and for pooled period data (B1 and B2) within groups (white cells).

[3] Correlations are shown for pooled period data for all participants (grey cells) and pooled period data within treatment groups (white cells) for follow-up scores and for change scores measured as additive, symmetric percentages (sympercents; see main text).

Abbreviations: ARA, arachidonic acid; ALA, α-linolenic acid; DGLA, dihomo-γ-linolenic acid; DHA, docosahexaenoic acid; DPA, docosapentaenoic acid; EPA, eicosapentaenoic acid; GLA, γ-linolenic acid; LA, linoleic acid; n-3, omega-3 PUFAs; n-6, omega-6 PUFAs; RBCMs, red blood cell membranes; wt%, weight percentage of total fatty acids; 95% BCa CI, 95% bootstrapped (bias-corrected and accelerated, BCa) confidence interval.

Supplemental Table 5. Absolute changes in inflammatory markers after 7 wk of supplementation with n-3 or n-6 PUFAs [1]

Variable and treatment	Baseline (n= 39)[2]	Follow-up (n=38)[2]	Absolute change[3]	Time[4]	Tx ×
**Acute-phase proteins**					
**hsCRP, µg/ml[6]**					0.280
n-3	2.21 (1.65)	2.71 (2.92)	0.040 (-0.43, 0.51)	0.867	
n-6	2.54 (2.60)	3.11 (3.28)	0.47 (-0.39, 1.32)	0.283	
**SAAt, µg/ml[6]**					0.114
n-3	2.79 (1.70)	3.82 (4.44)	-0.10 (-0.87, 0.66)	0.787	
n-6	3.66 (2.83)	6.48 (10.3)	1.32 (-0.39, 3.03)	0.128	
**SAA1t, µg/ml[6]**					0.117
n-3	2.13 (1.32)	3.03 (3.91)	-0.094 (-0.68, 0.50)	0.753	
n-6	2.82 (2.29)	5.02 (7.97)	0.96 (-0.29, 2.22)	0.131	
**SAA2t, µg/ml[6]**					0.102
n-3	0.66 (0.46)	0.79 (0.66)	0.067 (-0.15, 0.28)	0.547	
n-6	0.84 (0.69)	1.46 (2.59)	0.44 (-0.062, 0.95)	0.085	
**S100At, µg/ml[6]**					0.613
n-3	3.41 (4.12)	3.90 (6.23)	0.71 (-0.58, 2.00)	0.275	
n-6	2.93 (2.58)	3.44 (2.56)	0.38 (-0.38, 1.13)	0.325	
**S100A8, µg/ml[6]**					0.633
n-3	2.40 (3.01)	2.80 (4.83)	0.50 (-0.45, 1.45)	0.296	
n-6	2.08 (1.95)	2.40 (1.89)	0.27 (-0.32, 0.85)	0.367	
**S100A9, µg/ml**					0.176
n-3	0.55 (0.66)	0.52 (0.44)	-0.048 (-0.17, 0.077)	0.447	
n-6	0.45 (0.35)	0.55 (0.39)	0.040 (-0.059, 0.14)	0.427	
**Cytokines and**					
**Eotaxin, pg/ml**					0.631
n-3	56.4 (20.4)	55.2 (24.0)	-1.17 (-3.83, 1.50)	0.387	
n-6	56.4 (21.1)	55.9 (22.0)	-0.45 (-3.11, 2.21)	0.738	
**IL-8, pg/ml[6]**					0.202
n-3	4.86 (2.90)	4.36 (2.31)	-0.53 (-1.08, 0.019)	0.058	
n-6	4.43 (1.85)	4.42 (2.25)	-0.15 (-0.67, 0.38)	0.574	
**MCP-1, pg/ml**					0.117
n-3	47.7 (21.7)	46.2 (21.7)	-1.87 (-4.75, 1.01)	0.201	
n-6	48.4 (19.9)	43.6 (18.4)	-4.43 (-7.31, -1.55)	0.003	
**MIP-1α, pg/ml[6]**					0.953
n-3	1.64 (0.56)	1.56 (0.58)	-0.072 (-0.18, 0.036)	0.189	
n-6	1.65 (0.61)	1.57 (0.63)	-0.068 (-0.18, 0.040)	0.213	
**MIP-1β pg/ml[6]**					0.471
n-3	20.2 (11.1)	17.7 (10.4)	-2.72 (-4.18, -1.26)		
n-6	21.0 (12.0)	18.8 (14.4)	-1.95 (-3.89, -0.001)	0.050	
**RANTES, pg/ml**					0.236
n-3	11.1 (3.08)	9.85 (2.74)	-0.95 (-1.70, -0.20)	0.013	
n-6	11.6 (4.63)	10.3 (2.78)	-0.70 (-1.44, 0.054)	0.069	
**TNF, pg/ml**					0.061
n-3	10.0 (2.88)	7.97 (3.13)	-1.95 (-2.52, -1.39)		
n-6	9.97 (3.00)	8.78 (3.84)	-1.24 (-1.94, -0.53)	0.001	

**IL-1RA, pg/ml**[6]					0.288
n-3	158 (84.5)	157 (128)	6.03 (-30.4, 42.5)	0.744	
n-6	144 (87.4)	139 (85.8)	-14.5 (-32.1, 3.22)	0.108	
**Other adipokines**					
**Adiponectin, pg/ml**[6]					0.514
n-3	113 (62.2)	108 (56.2)	-6.92 (-18.7, 4.85)	0.247	
n-6	132 (86.1)	112 (60.9)	-2.26 (-13.0, 8.51)	0.678	
**Apelin, ng/ml**[6]					0.293
n-3	315 (289)	345 (309)	12.3 (-89.7, 114)	0.812	
n-6	452 (811)	351 (570)	-32.0 (-140, 76.1)	0.559	

[1] Pooled period data of fasting serum and plasma levels were analyzed with cLMM adjusted for the main effect of period and subject-averaged baselines, Values were transformed by the natural logarithm before the analyses.
[2] Values are arithmetic means (SDs) of fasting blood levels at baseline and follow-up.
[3] Absolute model-adjusted mean change scores (95% CIs) from baseline to follow-up.
[4] P-values for absolute changes from baseline to follow-up within treatments (time effects).
[5] P-values for absolute changes from baseline to follow-up between treatments (group differences in time effects).
[6] One influential outlier was excluded from the final analyses of hsCRP, SAAt, SAA1t, SAA2t, S100A8, S100At, Il-8, MIP-1α, MIP-1β, IL-1RA, and adiponectin, and two outliers were excluded from apelin.
Abbreviations: cLMM, constrained linear-mixed effects model; Tx, treatment.

Supplemental Table 6. Period-specific responses in relative changes for circulating levels of inflammatory markers after 7 wk of supplementation with n-3 or n-6 PUFAs[1]

Variable and group	Baseline[2]	Follow-up[2]	Relative change[3]	Time[4]	Time × prd[5]	Group × prd[6]
**Acute-phase proteins**						
**hsCRP, µg/ml[7]**						
**n-3 : P1**	1.53 (0.64, 3.65)	1.64 (0.55, 4.93)	13.7 (-18.3, 58.2)	0.444	0.576	0.831
**n-3 : P2**	1.80 (0.82, 3.92)	1.83 (0.78, 4.27)	1.77 (-17.5, 25.5)	0.869		0.549
**n-6 : P1**	2.17 (0.94, 5.00)	2.35 (1.06, 5.22)	8.55 (-17.2, 42.3)	0.549	0.712	
**n-6 : P2**	1.44 (0.58, 3.54)	1.75 (0.58, 5.31)	21.4 (-29.5, 109)	0.481		
**SAAt, µg/ml[7]**						
**n-3 : P1**	2.41 (1.40, 4.14)	3.43 (1.71, 6.90)	42.0 (2.66, 96.4)	0.034	0.217	0.684
**n-3 : P2**	2.31 (1.21, 4.39)	2.52 (1.45, 4.38)	9.22 (-16.2, 42.4)	0.512		0.516
**n-6 : P1**	2.94 (1.37, 6.34)	3.66 (1.79, 7.45)	24.2 (-29.4, 118)	0.448	0.892	
**n-6 : P2**	2.93 (1.72, 5.00)	3.82 (1.30, 11.2)	30.6 (-18.8, 110)	0.267		
**SAA1t, µg/ml[7]**						
**n-3 : P1**	1.78 (0.99, 3.18)	2.61 (1.26, 5.41)	46.3 (0.11, 114)	0.049	0.213	0.501
**n-3 : P2**	1.79 (0.94, 3.41)	1.96 (1.11, 3.48)	9.51 (-15.4, 41.7)	0.487		0.565
**n-6 : P1**	2.29 (1.05, 5.00)	2.81 (1.38, 5.73)	22.6 (-13.9, 74.7)	0.255	0.838	
**n-6 : P2**	2.21 (1.31, 3.72)	2.89 (0.96, 8.69)	31.5 (-25.9, 133)	0.346		
**SAA2t, µg/ml[7]**						
**n-3 : P1**	0.57 (0.31, 1.06)	0.73 (0.33, 1.60)	26.5 (-9.20, 76.2)	0.163	0.517	0.928
**n-3 : P2**	0.49 (0.23, 1.02)	0.53 (0.30, 0.93)	8.03 (-23.7, 52.9)	0.660		0.592
**n-6 : P1**	0.63 (0.30, 1.34)	0.82 (0.38, 1.73)	29.8 (-19.2, 109)	0.277	0.949	
**n-6 : P2**	0.66 (0.32, 1.34)	0.84 (0.28, 2.50)	27.0 (-21.9, 107)	0.332		
**S100At, µg/ml[7]**						
**n-3 : P1**	2.24 (0.91, 5.56)	2.14 (0.78, 5.90)	-2.31 (-32.6, 41.7)	0.901	0.376	0.640
**n-3 : P2**	2.12 (0.76, 5.93)	2.61 (1.13, 6.02)	23.4 (-14.4, 78.0)	0.257		0.229
**n-6 : P1**	2.51 (1.16, 5.42)	2.20 (0.94, 5.14)	-12.6 (-34.4, 16.5)	0.355	0.004	
**n-6 : P2**	1.82 (0.78, 4.22)	3.02 (1.41, 6.46)	66.7 (19.9, 132)	0.003		
**S100A8, µg/ml[7]**						
**n-3 : P1**	1.50 (0.57, 3.95)	1.45 (0.50, 4.20)	-1.55 (-34.3, 47.5)	0.939	0.366	0.498
**n-3 : P2**	1.40 (0.45, 4.31)	1.78 (0.73, 4.35)	27.3 (-15.6, 92.2)	0.247		0.295
**n-6 : P1**	1.75 (0.77, 3.95)	1.45 (0.57, 3.64)	-17.2 (-38.9, 12.1)	0.220	0.003	
**n-6 : P2**	1.24 (0.52, 2.96)	2.09 (0.95, 4.60)	69.0 (20.4, 137)	0.003		
**S100A9, µg/ml**						
**n-3 : P1**	0.39 (0.17, 0.90)	0.32 (0.13, 0.76)	-15.5 (-33.5, 7.33)	0.166	0.050	0.508
**n-3 : P2**	0.34 (0.12, 0.94)	0.44 (0.19, 0.99)	28.3 (-8.92, 80.7)	0.152		0.390
**n-6 : P1**	0.39 (0.19, 0.79)	0.37 (0.17, 0.81)	-4.83 (-26.8, 23.7)	0.709	0.020	
**n-6 : P2**	0.30 (0.12, 0.72)	0.47 (0.22, 1.04)	58.0 (13.1, 121)	0.008		
**Other adipokines**						
**Eotaxin, pg/ml**						
**n-3 : P1**	59.1 (41.1, 85.0)	57.3 (38.0, 86.4)	-2.97 (-10.4, 5.09)	0.456	0.682	0.676
**n-3 : P2**	46.7 (32.1, 67.8)	44.2 (28.4, 68.6)	-5.34 (-13.4, 3.47)	0.224		0.659
**n-6 : P1**	49.8 (34.1, 72.8)	49.5 (33.3, 73.6)	-0.63 (-8.25, 7.62)	0.876	0.734	
**n-6 : P2**	55.4 (37.2, 82.4)	53.9 (35.1, 82.7)	-2.65 (-10.9, 6.42)	0.551		
**IL-8, pg/ml[7]**						
**n-3 : P1**	4.64 (2.30, 9.36)	4.80 (3.06, 7.52)	2.65 (-24.7, 40.0)	0.868	0.152	0.764

Variable and group	Baseline[2]	Follow-up[2]	Relative change[3]	Time[4]	Time × prd[5]	Group × prd[6]
**n-3 : P2**	3.52 (2.02, 6.14)	2.87 (1.47, 5.60)	-23.6 (-41.2, -0.75)	0.044		0.243
**n-6 : P1**	3.21 (1.13, 9.10)	3.52 (2.05, 6.04)	9.78 (-19.9, 50.5)	0.559	0.480	
**n-6 : P2**	4.42 (2.90, 6.72)	4.19 (2.33, 7.57)	-5.06 (-26.5, 22.7)	0.688		
**MCP-1, pg/ml**						
**n-3 : P1**	53.3 (37.4, 76.0)	51.9 (36.3, 74.2)	-2.84 (-12.0, 7.34)	0.568	0.604	0.725
**n-3 : P2**	34.9 (22.8, 53.5)	32.7 (19.5, 54.9)	-6.31 (-15.0, 3.27)	0.187		0.283
**n-6 : P1**	40.8 (26.9, 61.9)	38.7 (25.4, 58.8)	-5.35 (-15.1, 5.55)	0.320	0.252	
**n-6 : P2**	49.0 (33.2, 72.3)	42.0 (28.6, 61.7)	-14.3 (-24.8, -2.27)	0.022		
**MIP-1α, pg/ml**[7]						
**n-3 : P1**	1.59 (1.10, 2.30)	1.59 (1.12, 2.24)	1.79 (-10.6, 15.9)	0.787	0.133	0.456
**n-3 : P2**	1.50 (1.05, 2.15)	1.32 (0.84, 2.10)	-11.8 (-23.0, 1.00)	0.069		0.464
**n-6 : P1**	1.58 (1.00, 2.48)	1.50 (0.90, 2.48)	-5.04 (-16.6, 8.17)	0.433	0.976	
**n-6 : P2**	1.46 (0.95, 2.24)	1.38 (0.95, 2.00)	-5.31 (-17.3, 8.44)	0.427		
**MIP-1β pg/ml**[7]						
**n-3 : P1**	17.8 (10.7, 29.6)	17.2 (11.6, 25.4)	-4.20 (-18.4, 12.4)	0.596	0.015	0.234
**n-3 : P2**	16.6 (8.04, 34.4)	11.1 (3.53, 35.1)	-33.1 (-47.2, -15.1)	0.001		0.452
**n-6 : P1**	22.1 (13.2, 36.9)	18.6 (9.12, 38.0)	-15.7 (-26.7, -3.14)	0.016	0.498	
**n-6 : P2**	14.8 (8.10, 27.0)	11.3 (5.37, 23.7)	-23.6 (-40.6, -1.63)	0.037		
**RANTES, ng/ml**						
**n-3 : P1**	10.5 (7.30, 15.0)	9.49 (6.74, 13.4)	-7.62 (-14.7, 0.087)	0.052	0.394	0.050
**n-3 : P2**	10.7 (8.14, 14.1)	9.35 (6.91, 12.6)	-12.7 (-21.1, -3.28)	0.010		0.082
**n-6 : P1**	12.8 (9.41, 17.5)	10.6 (8.14, 13.7)	-17.6 (-24.0, -10.6)	<0.001	0.005	
**n-6 : P2**	9.30 (6.55, 13.2)	9.32 (6.70, 13.0)	-0.78 (-10.3, 9.81)	0.879		
**TNF, pg/ml**						
**n-3 : P1**	10.1 (7.79, 13.0)	8.61 (5.74, 12.9)	-14.4 (-27.1, 0.47)	0.057	0.027	0.932
**n-3 : P2**	9.01 (6.02, 13.5)	5.91 (3.23, 10.8)	-34.5 (-44.8, -22.2)	<0.001		0.144
**n-6 : P1**	11.0 (8.11, 14.9)	9.32 (5.06, 17.2)	-15.2 (-27.8, -0.50)	0.043	0.527	
**n-6 : P2**	8.12 (5.74, 11.5)	6.39 (3.88, 10.5)	-21.4 (-33.9, -6.53)	0.007		
**IL-1RA, pg/ml**[7]						
**n-3 : P1**	155 (95.4, 251)	82.7 (14.4, 476)	-45.7 (-70.0, -1.92)	0.043	0.024	0.142
**n-3 : P2**	115 (52.9, 248)	133 (62.8, 280)	15.1 (-12.1, 50.8)	0.303		0.509
**n-6 : P1**	141 (75.2, 266)	132 (78.0, 223)	-11.4 (-33.5, 18.1)	0.407	0.580	
**n-6 : P2**	75.3 (13.7, 414)	75.7 (14.4, 397)	0.13 (-27.2, 37.7)	0.993		
**Other adipokines**						
**Adiponectin, pg/ml**[7]						
**n-3 : P1**	104 (60.4, 180)	104 (62.8, 173)	0.074 (-20.2, 25.5)	0.995	0.531	0.814
**n-3 : P2**	91.9 (50.9, 166)	82.9 (43.6, 158)	-9.80 (-28.8, 14.3)	0.390		0.757
**n-6 : P1**	97.5 (42.0, 226)	101 (57.3, 179)	3.97 (-17.1, 30.4)	0.735	0.242	
**n-6 : P2**	110 (58.5, 208)	94.5 (56.2, 159)	-14.4 (-32.4, 8.46)	0.196		
**Apelin, ng/ml**[7]						
**n-3 : P1**	190 (60.3, 596)	151 (35.9, 635)	-20.4 (-59.8, 57.8)	0.510	0.157	0.430
**n-3 : P2**	177 (39.3, 797)	307 (126, 751)	73.7 (-25.1, 303)	0.196		0.824
**n-6 : P1**	272 (84.0, 883)	147 (37.5, 579)	-45.9 (-72.7, 7.18)	0.078	0.062	
**n-6 : P2**	155 (34.2, 701)	235 (79.6, 694)	51.9 (-34.5, 252)	0.326		

[1] Fasting blood levels were analyzed with cLMM. Values were transformed by the natural logarithm before the analyses.

[2] Values are geometric means (1 SD ranges) of fasting blood levels at baseline and follow-up within treatments and periods.
[3] Relative model-adjusted mean change scores (95% CIs) from baseline to follow-up as percentages calculated from the model estimates, % = $(\exp^{\text{estimate}} - 1) \times 100$.
[4] P-values for relative changes from baseline to follow-up within treatments and periods (time effects).
[5] P-values for relative changes from baseline to follow-up within treatments and between periods (period differences in time effects), The first and second values refer to between-period differences after the n-3 and n-6 interventions, respectively.
[6] P-values for relative changes from baseline to follow-up between treatments and within periods (group differences in time effects within each stratum of period), The first and second values refer to between-treatment differences in P1 and P2, respectively.
[7] One influential outlier was excluded from the final analyses of hsCRP, SAAt, SAA1t, SAA2t, S100A8, S100At, Il-8, MIP-1α, MIP-1β, IL-1RA, and adiponectin, and two outliers were excluded from apelin.
Abbreviations: CI, confidence interval; cLMM, constrained linear-mixed effects model; P1, period 1; P2, period 2; prd, period.

Supplemental Table 7. Relative changes of circulating levels of inflammatory markers after 7 wk of supplementation with n-3 or n-6 stratified by n-3 index in RBCMs[1]

Variable and group	Baseline[2]	Follow-up[2]	Relative change[3]	Time[4]	Time × CVR[5]	Group × CVR [6]
**Acute-phase proteins**						
**hsCRP, µg/ml[7]**						
**n-3: Low n-3 index**	2.09 (1.05, 4.19)	2.73 (1.07, 6.97)	0.64 (-0.088, 1.37)	0.085		0.638
**n-3: High n-3 index**	1.45 (0.61, 3.46)	1.33 (0.54, 3.28)	0.033 (-0.47, 0.54)	0.896	0.178	0.049
**n-6: Low n-3 index**	2.63 (1.33, 5.19)	2.28 (1.11, 4.67)	0.36 (-0.82, 1.55)	0.553		
**n-6: High n-3 index**	1.40 (0.56, 3.50)	1.90 (0.64, 5.65)	0.90 (0.035, 1.77)	0.041	0.451	
**SAAt, µg/ml[7]**						
**n-3: Low n-3 index**	2.44 (1.24, 4.80)	3.74 (1.71, 8.19)	2.30 (-0.16, 4.82)	0.067		0.892
**n-3: High n-3 index**	2.31 (1.34, 3.98)	2.56 (1.54, 4.25)	0.12 (-1.73, 2.00)	0.900	0.166	0.050
**n-6: Low n-3 index**	3.15 (1.67, 5.96)	3.46 (1.69, 7.09)	1.86 (-4.03, 8.10)	0.541		
**n-6: High n-3 index**	2.82 (1.44, 5.51)	3.91 (1.43, 10.7)	5.06 (0.41, 9.94)	0.033	0.413	
**SAA1t, µg/ml[7]**						
**n-3: Low n-3 index**	1.76 (0.87, 3.56)	2.81 (1.24, 6.38)	0.32 (-0.65, 1.30)	0.514		0.592
**n-3: High n-3 index**	1.80 (1.03, 3.13)	2.00 (1.18, 3.39)	-0.091 (-0.79, 0.61)	0.797	0.474	0.008
**n-6: Low n-3 index**	2.32 (1.25, 4.31)	2.57 (1.19, 5.55)	0.91 (-1.29, 3.16)	0.416		
**n-6: High n-3 index**	2.21 (1.11, 4.39)	3.03 (1.12, 8.22)	1.98 (0.43, 3.55)	0.013	0.440	
**SAA2t, µg/ml[7]**						
**n-3: Low n-3 index**	0.61 (0.28, 1.34)	0.86 (0.41, 1.80)	0.25 (-0.11, 0.61)	0.174		0.856
**n-3: High n-3 index**	0.49 (0.27, 0.90)	0.51 (0.28, 0.94)	-0.006 (-0.28, 0.27)	0.967	0.268	0.043
**n-6: Low n-3 index**	0.77 (0.35, 1.68)	0.83 (0.46, 1.50)	0.12 (-1.32, 1.57)	0.873		
**n-6: High n-3 index**	0.58 (0.29, 1.15)	0.83 (0.28, 2.45)	1.14 (0.031, 2.26)	0.044	0.270	
**S100At, µg/ml[7]**						
**n-3: Low n-3 index**	2.11 (0.75, 5.95)	2.91 (0.99, 8.54)	1.35 (-0.41, 3.14)	0.132		0.234
**n-3: High n-3 index**	2.22 (0.88, 5.63)	2.10 (0.93, 4.75)	0.28 (-0.96, 1.54)	0.656	0.333	0.505
**n-6: Low n-3 index**	2.70 (1.44, 5.07)	2.83 (1.42, 5.63)	0.20 (-1.13, 1.55)	0.763		
**n-6: High n-3 index**	1.86 (0.77, 4.52)	2.44 (1.01, 5.91)	0.74 (-0.30, 1.79)	0.162	0.532	
**S100A8, µg/ml[7]**						
**n-3: Low n-3 index**	1.40 (0.46, 4.24)	1.97 (0.63, 6.14)	0.98 (-0.32, 2.30)	0.138		0.256

Variable and group	Baseline[2]	Follow-up[2]	Relative change[3]	Time[4]	Time × CVR[5]	Group × CVR [6]
**n-3: High n-3 index**	1.48 (0.54, 4.08)	1.42 (0.59, 3.41)	0.20 (-0.72, 1.14)	0.669	0.338	0.493
**n-6: Low n-3 index**	1.83 (0.95, 3.55)	1.89 (0.89, 4.00)	0.18 (-0.80, 1.18)	0.715		
**n-6: High n-3 index**	1.29 (0.51, 3.29)	1.65 (0.64, 4.24)	0.55 (-0.23, 1.33)	0.165	0.566	
**S100A9, µg/ml**						
**n-3: Low n-3 index**	0.35 (0.13, 0.95)	0.45 (0.19, 1.06)	0.034 (-0.15, 0.22)	0.710		0.558
**n-3: High n-3 index**	0.37 (0.15, 0.90)	0.33 (0.14, 0.77)	-0.004 (-0.15, 0.14)	0.957	0.745	0.317
**n-6: Low n-3 index**	0.47 (0.25, 0.89)	0.46 (0.24, 0.89)	-0.029 (-0.20, 0.14)	0.744		
**n-6: High n-3 index**	0.28 (0.12, 0.66)	0.40 (0.17, 0.93)	0.079 (-0.052, 0.21)	0.235	0.329	
**Cytokines and chemokines**						
**Eotaxin, pg/ml**						
**n-3: Low n-3 index**	55.8 (41.4, 75.0)	53.1 (36.9, 76.5)	-0.12 (-4.34, 4.29)	0.955		0.154
**n-3: High n-3 index**	51.0 (33.3, 78.1)	48.7 (30.1, 79.0)	-1.76 (-4.95, 1.53)	0.288	0.548	0.628
**n-6: Low n-3 index**	51.4 (34.8, 76.1)	55.6 (37.4, 82.7)	3.42 (-0.95, 7.98)	0.126		
**n-6: High n-3 index**	53.2 (35.9, 78.7)	49.5 (32.6, 75.1)	-2.64 (-5.80, 0.62)	0.111	0.030	
**IL-8, pg/ml**[7]						
**n-3: Low n-3 index**	4.93 (2.66, 9.13)	4.33 (2.11, 8.90)	0.11 (-0.80, 1.03)	0.809		0.519
**n-3: High n-3 index**	3.64 (1.91, 6.92)	3.44 (1.98, 5.99)	-0.52 (-1.22, 0.18)	0.144	0.260	0.728
**n-6: Low n-3 index**	3.34 (1.00, 11.2)	4.04 (2.16, 7.57)	-0.24 (-1.15, 0.68)	0.610		
**n-6: High n-3 index**	4.04 (2.61, 6.25)	3.73 (2.18, 6.38)	-0.38 (-1.07, 0.32)	0.279	0.796	
**MCP-1, pg/ml**						
**n-3: Low n-3 index**	48.7 (33.0, 71.7)	47.5 (32.1, 70.5)	2.18 (-2.17, 6.73)	0.327		0.352
**n-3: High n-3 index**	40.7 (25.6, 64.7)	37.9 (22.1, 65.0)	-3.80 (-6.85, -0.65)	0.019	0.029	0.232
**n-6: Low n-3 index**	44.6 (30.7, 64.9)	45.5 (32.0, 64.6)	-0.30 (-4.94, 4.56)	0.900		
**n-6: High n-3 index**	44.8 (29.0, 69.1)	37.6 (24.8, 56.8)	-6.07 (-9.37, -2.65)	0.001	0.050	
**MIP-1α, pg/ml**[7]						
**n-3: Low n-3 index**	1.69 (1.26, 2.25)	1.68 (1.24, 2.30)	0.025 (-0.15, 0.20)	0.782		0.145
**n-3: High n-3 index**	1.47 (0.99, 2.19)	1.33 (0.85, 2.07)	-0.13 (-0.26, 0.006)	0.062	0.175	0.234
**n-6: Low n-3 index**	1.53 (0.90, 2.58)	1.50 (1.07, 2.12)	-0.13 (-0.30, 0.051)	0.160		
**n-6: High n-3 index**	1.51 (1.02, 2.23)	1.40 (0.86, 2.29)	-0.035 (-0.17, 0.10)	0.611	0.417	

Variable and group	Baseline[2]	Follow-up[2]	Relative change[3]	Time[4]	Time × CVR[5]	Group × CVR [6]
**MIP-1β pg/ml[7]**						
**n-3: Low n-3 index**	22.0 (14.0, 34.5)	20.6 (14.0, 30.5)	-1.81 (-4.86, 1.34)	0.255		0.456
**n-3: High n-3 index**	15.0 (7.72, 29.1)	11.0 (4.06, 29.6)	-3.58 (-5.90, -1.20)	0.004	0.366	0.602
**n-6: Low n-3 index**	21.0 (13.0, 33.8)	17.4 (8.19, 36.9)	-0.23 (-3.37, 3.01)	0.889		
**n-6: High n-3 index**	16.6 (8.75, 31.3)	13.0 (6.08, 28.0)	-2.75 (-5.08, -0.36)	0.024	0.207	
**RANTES, pg/ml**						
**n-3: Low n-3 index**	9.80 (6.99, 13.8)	8.67 (6.35, 11.8)	-1.18 (-2.64, 0.30)	0.116		0.547
**n-3: High n-3 index**	11.1 (8.15, 15.0)	9.88 (7.18, 13.6)	-1.66 (-2.76, -0.54)	0.004	0.607	0.303
**n-6: Low n-3 index**	9.80 (7.71, 12.4)	9.06 (7.11, 11.6)	-0.78 (-2.24, 0.71)	0.301		
**n-6: High n-3 index**	11.6 (7.70, 17.6)	10.5 (7.58, 14.5)	-1.13 (-2.24, -0.013)	0.048	0.703	
**TNF, pg/ml**						
**n-3: Low n-3 index**	9.06 (5.91, 13.9)	6.62 (3.33, 13.2)	-1.55 (-2.56, -0.52)	0.004		0.540
**n-3: High n-3 index**	9.81 (7.43, 13.0)	7.45 (4.75, 11.7)	-2.01 (-2.79, -1.22)	<0.001	0.481	0.151
**n-6: Low n-3 index**	8.18 (5.06, 13.2)	6.33 (2.77, 14.5)	-1.14 (-2.31, 0.054)	0.061		
**n-6: High n-3 index**	10.3 (8.15, 13.0)	8.66 (6.12, 12.3)	-1.25 (-2.18, -0.32)	0.009	0.875	
**IL-1RA, pg/ml[7]**						
**n-3: Low n-3 index**	152 (98.3, 235)	142 (67.5, 298)	6.97 (-18.8, 40.9)	0.629		0.398
**n-3: High n-3 index**	124 (59.2, 261)	87.8 (17.9, 430)	-25.2 (-41.2, -4.76)	0.019	0.028	0.331
**n-6: Low n-3 index**	130 (71.3, 236)	116 (52.3, 259)	-7.80 (-29.4, 20.4)	0.548		
**n-6: High n-3 index**	90.3 (18.6, 438)	90.0 (20.5, 395)	-14.0 (-30.0, 5.73)	0.151	0.679	
**Other adipokines**						
**Adiponectin, pg/ml[7]**						
**n-3: Low n-3 index**	84.8 (50.5, 142)	69.0 (37.1, 128)	-19.3 (-33.0, -2.85)	0.024		0.546
**n-3: High n-3 index**	106 (59.4, 191)	111 (67.7, 181)	-2.49 (-15.6, 12.6)	0.729	0.112	0.961
**n-6: Low n-3 index**	104 (50.9, 212)	82.9 (49.7, 138)	-12.2 (-33.2, 15.4)	0.347		
**n-6: High n-3 index**	104 (48.1, 223)	108 (62.7, 185)	-3.01 (-21.5, 19.9)	0.775	0.569	
**Apelin, ng/ml[7]**						
**n-3: Low n-3 index**	157 (31.0, 793)	183 (33.5, 998)	141 (-69.3, 1791)	0.399		0.326
**n-3: High n-3 index**	200 (64.3, 625)	237 (96.9, 578)	-17.5 (-85.4, 364)	0.825	0.340	0.265

Variable and group	Baseline[2]	Follow-up[2]	Relative change[3]	Time[4]	Time × CVR[5]	Group × CVR [6]
**n-6: Low n-3 index**	164 (29.7, 910)	186 (27.6, 1254)	428 (-35.2, 4194)	0.119		
**n-6: High n-3 index**	234 (74.8, 730)	186 (97.6, 355)	-57.6 (-92.4, 138)	0.326	0.030	

[1] Fasting blood levels were analyzed with cLMM, Values were transformed by the natural logarithm before the analyses.
[2] Values are geometric means (1 SD ranges) of fasting blood levels at baseline and follow-up within treatments and periods.
[3] Relative model-adjusted mean change scores (95% CIs) from baseline to follow-up as percentages calculated from the model estimates, $\% = (\exp^{estimate} - 1) \times 100$.
[4] P-values for relative changes from baseline to follow-up within treatments and different n-3 index levels (time effects).
[5] P-values for relative changes from baseline to follow-up within treatments and between high and low n-3 index. The first and second values refer to differences between low and high n-3 index after the n-3 and n-6 interventions, respectively.
[6] P-values for relative changes from baseline to follow-up between treatments and within low and high n-3 index. The first and second values refer to between-treatment differences in low and high n-3 index, respectively.
[7] One influential outlier was excluded from the final analyses of hsCRP, SAAt, SAA1t, SAA2t, S100A8, S100At, Il-8, MIP-1α, MIP-1β, IL-1RA, and adiponectin, and two outliers were excluded from apelin.
Abbreviations: cLMM, constrained linear-mixed effects model; CVR, Covariate Ratio; RBCMs, red blood cell membranes.

Supplemental Table 8. Relative changes of circulating levels of inflammatory markers after 7 wk of supplementation with n-3 or n-6 stratified by LA level in RBCMs[1]

Variable and group	Baseline[2]	Follow-up[2]	Relative change[3]	Time[4]	Time × CVR[5]	Group × CVR [6]
**Acute-phase proteins**						
**hsCRP, µg/ml[7]**						
**n-3: Low LA**	1.70 (0.81, 3.58)	1.76 (0.76, 4.07)	0.50 (-0.047, 1.04)	0.073		0.361
**n-3: High LA**	1.62 (0.66, 3.99)	1.71 (0.57, 5.11)	-0.009 (-0.56, 0.55)	0.975	0.200	0.239
**n-6: Low LA**	1.88 (0.85, 4.13)	2.03 (0.91, 4.52)	0.96 (-0.073, 2.00)	0.068		
**n-6: High LA**	1.67 (0.63, 4.44)	2.03 (0.67, 6.18)	0.59 (-0.44, 1.63)	0.259	0.604	
**SAAt, µg/ml[7]**						
**n-3: Low LA**	2.05 (1.22, 3.46)	2.97 (1.91, 4.62)	0.88 (-1.33, 3.14)	0.437		0.705
**n-3: High LA**	2.69 (1.44, 5.03)	2.91 (1.32, 6.42)	1.07 (-1.04, 3.23)	0.318	0.900	0.088
**n-6: Low LA**	3.07 (1.82, 5.16)	3.24 (1.81, 5.82)	1.97 (-3.19, 7.40)	0.459		
**n-6: High LA**	2.83 (1.32, 6.05)	4.25 (1.40, 12.9)	5.84 (0.74, 11.2)	0.025	0.304	
**SAA1t, µg/ml[7]**						
**n-3: Low LA**	1.54 (0.91, 2.61)	2.25 (1.40, 3.62)	0.50 (-0.26, 1.26)	0.196		0.135
**n-3: High LA**	2.05 (1.07, 3.93)	2.28 (1.01, 5.11)	-0.41 (-1.19, 0.37)	0.298	0.084	0.029
**n-6: Low LA**	2.33 (1.39, 3.91)	2.43 (1.39, 4.23)	1.89 (-0.023, 3.83)	0.053		
**n-6: High LA**	2.18 (1.01, 4.70)	3.30 (1.05, 10.3)	1.69 (-0.28, 3.70)	0.092	0.892	
**SAA2t, µg/ml[7]**						
**n-3: Low LA**	0.46 (0.23, 0.93)	0.66 (0.36, 1.20)	0.12 (-0.20, 0.45)	0.446		0.668
**n-3: High LA**	0.61 (0.33, 1.14)	0.59 (0.27, 1.29)	0.064 (-0.24, 0.37)	0.679	0.789	0.103
**n-6: Low LA**	0.67 (0.34, 1.32)	0.76 (0.34, 1.68)	0.41 (-0.86, 1.69)	0.529		
**n-6: High LA**	0.62 (0.28, 1.35)	0.89 (0.31, 2.54)	1.09 (-0.12, 2.31)	0.078	0.445	
**S100At, µg/ml[7]**						
**n-3: Low LA**	2.00 (0.71, 5.65)	2.08 (0.96, 4.55)	0.42 (-1.04, 1.91)	0.569		0.772
**n-3: High LA**	2.37 (0.97, 5.76)	2.65 (0.94, 7.48)	0.85 (-0.64, 2.36)	0.263	0.690	0.645
**n-6: Low LA**	2.06 (1.02, 4.19)	2.45 (1.12, 5.36)	0.66 (-0.55, 1.89)	0.286		
**n-6: High LA**	2.20 (0.88, 5.50)	2.69 (1.15, 6.32)	0.48 (-0.66, 1.62)	0.409	0.828	
**S100A8, µg/ml[7]**						
**n-3: Low LA**	1.29 (0.42, 3.96)	1.40 (0.60, 3.25)	0.32 (-0.77, 1.42)	0.564		0.763
**n-3: High LA**	1.62 (0.62, 4.22)	1.82 (0.61, 5.39)	0.60 (-0.50, 1.72)	0.284	0.720	0.671
**n-6: Low LA**	1.41 (0.68, 2.94)	1.64 (0.70, 3.84)	0.50 (-0.41, 1.41)	0.281		
**n-6: High LA**	1.53 (0.58, 3.99)	1.83 (0.74, 4.51)	0.35 (-0.49, 1.20)	0.413	0.815	
**S100A9, µg/ml**						
**n-3: Low LA**	0.35 (0.13, 0.99)	0.35 (0.16, 0.76)	-0.037 (-0.20, 0.13)	0.656		0.357
**n-3: High LA**	0.37 (0.16, 0.85)	0.39 (0.16, 0.99)	0.045 (-0.11, 0.20)	0.557	0.467	0.921
**n-6: Low LA**	0.34 (0.16, 0.74)	0.40 (0.19, 0.84)	0.052 (-0.10, 0.20)	0.504		
**n-6: High LA**	0.34 (0.14, 0.80)	0.44 (0.19, 1.01)	0.036 (-0.11, 0.18)	0.626	0.885	
**Other adipokines**						
**Eotaxin, pg/ml**						
**n-3: Low LA**	52.6 (34.6, 79.9)	49.5 (29.4, 83.2)	-1.63 (-5.37, 2.25)	0.401		0.715
**n-3: High LA**	52.8 (36.9, 75.4)	51.1 (35.4, 73.7)	-0.73 (-4.31, 2.98)	0.692	0.736	0.315
**n-6: Low LA**	54.9 (37.5, 80.3)	51.3 (34.1, 77.2)	-2.41 (-6.11, 1.44)	0.214		
**n-6: High LA**	50.5 (33.9, 75.2)	52.0 (34.2, 79.0)	1.34 (-2.31, 5.14)	0.472	0.164	
**IL-8, pg/ml[7]**						

Variable and group	Baseline[2]	Follow-up[2]	Relative change[3]	Time[4]	Time × CVR[5]	Group × CVR [6]
**n-3: Low LA**	3.82 (2.21, 6.61)	3.44 (2.07, 5.73)	-0.55 (-1.35, 0.26)	0.178		0.579
**n-3: High LA**	4.29 (2.07, 8.92)	4.03 (1.98, 8.21)	-0.055 (-0.82, 0.72)	0.886	0.363	0.489
**n-6: Low LA**	3.85 (2.53, 5.87)	3.54 (1.86, 6.74)	-0.29 (-1.08, 0.50)	0.463		
**n-6: High LA**	3.69 (1.30, 10.5)	4.13 (2.53, 6.74)	-0.36 (-1.13, 0.40)	0.348	0.895	
**MCP-1, pg/ml**						
**n-3: Low LA**	41.9 (26.4, 66.6)	40.6 (24.0, 68.6)	-1.26 (-5.04, 2.67)	0.521		0.136
**n-3: High LA**	44.8 (29.2, 68.9)	41.8 (25.7, 67.9)	-2.16 (-5.78, 1.60)	0.254	0.740	0.508
**n-6: Low LA**	46.2 (30.2, 70.7)	39.9 (26.8, 59.4)	-4.58 (-8.75, -0.22)	0.040		
**n-6: High LA**	43.4 (29.1, 64.8)	40.7 (27.0, 61.2)	-3.57 (-7.62, 0.67)	0.097	0.735	
**MIP-1α, pg/ml[7]**						
**n-3: Low LA**	1.62 (1.14, 2.30)	1.44 (0.91, 2.30)	-0.15 (-0.30, 0.009)	0.065		0.418
**n-3: High LA**	1.48 (1.02, 2.16)	1.46 (1.00, 2.11)	-0.003 (-0.15, 0.15)	0.970	0.187	0.491
**n-6: Low LA**	1.71 (1.22, 2.38)	1.53 (0.98, 2.38)	-0.074 (-0.23, 0.083)	0.355		
**n-6: High LA**	1.36 (0.83, 2.25)	1.36 (0.88, 2.10)	-0.063 (-0.21, 0.086)	0.406	0.921	
**MIP-1β pg/ml[7]**						
**n-3: Low LA**	16.4 (9.60, 28.2)	11.8 (4.17, 33.5)	-2.91 (-4.98, -0.80)	0.008		0.949
**n-3: High LA**	18.0 (8.95, 36.0)	16.0 (8.03, 31.7)	-2.51 (-4.47, -0.52)	0.014	0.785	0.328
**n-6: Low LA**	18.9 (11.9, 29.9)	14.0 (7.04, 27.8)	-2.81 (-5.55, 0.006)	0.051		
**n-6: High LA**	17.4 (8.67, 34.8)	15.0 (6.46, 34.7)	-1.10 (-3.71, 1.59)	0.417	0.378	
**RANTES, ng/ml**						
**n-3: Low LA**	10.8 (7.62, 15.2)	9.04 (6.40, 12.8)	-2.10 (-3.35, -0.84)	0.001		0.358
**n-3: High LA**	10.4 (7.74, 14.0)	9.77 (7.26, 13.1)	-0.92 (-2.13, 0.30)	0.136	0.184	0.447
**n-6: Low LA**	10.9 (6.78, 17.5)	9.67 (6.82, 13.7)	-1.56 (-2.82, -0.29)	0.016		
**n-6: High LA**	11.0 (8.59, 14.0)	10.2 (7.87, 13.1)	-0.50 (-1.71, 0.73)	0.424	0.231	
**TNF, pg/ml**						
**n-3: Low LA**	9.26 (6.39, 13.4)	6.32 (3.27, 12.2)	-2.38 (-3.34, -1.42)	<0.001		0.491
**n-3: High LA**	9.81 (7.22, 13.3)	7.95 (5.35, 11.8)	-1.65 (-2.56, -0.73)	0.001	0.271	0.033
**n-6: Low LA**	9.87 (7.58, 12.9)	6.92 (3.56, 13.4)	-1.99 (-2.95, -1.03)	<0.001		
**n-6: High LA**	9.08 (5.93, 13.9)	8.52 (5.20, 13.9)	-0.48 (-1.40, 0.45)	0.311	0.026	
**IL-1RA, pg/ml[7]**						
**n-3: Low LA**	129 (61.1, 271)	75.0 (13.3, 422)	-22.0 (-41.3, 3.56)	0.085		0.981
**n-3: High LA**	138 (79.0, 243)	142 (62.1, 322)	-4.25 (-25.9, 23.7)	0.737	0.224	0.771
**n-6: Low LA**	93.1 (15.3, 566)	75.7 (13.8, 414)	-22.4 (-38.6, -1.77)	0.035		
**n-6: High LA**	113 (60.5, 211)	128 (74.4, 220)	0.32 (-20.6, 26.7)	0.978	0.123	
**Other adipokines**						
**Adiponectin, pg/ml[7]**						
**n-3: Low LA**	89.1 (47.1, 169)	94.9 (45.8, 197)	-0.85 (-15.6, 16.4)	0.917		0.479
**n-3: High LA**	107 (65.1, 174)	91.3 (59.4, 140)	-14.7 (-26.6, -1.02)	0.036	0.175	0.231
**n-6: Low LA**	115 (52.2, 254)	91.3 (51.4, 162)	-9.39 (-29.4, 16.2)	0.434		
**n-6: High LA**	94.4 (47.1, 189)	104 (62.5, 174)	-1.59 (-22.1, 24.3)	0.892	0.632	
**Apelin, ng/ml[7]**						
**n-3: Low LA**	189 (36.5, 975)	197 (39.0, 999)	12.6 (-84.2, 703)	0.905		0.429
**n-3: High LA**	178 (66.4, 479)	233 (107, 506)	21.7 (-81.6, 703)	0.837	0.945	0.830
**n-6: Low LA**	244 (98.2, 608)	181 (38.1, 859)	-35.1 (-90.7, 354)	0.660		
**n-6: High LA**	176 (32.6, 945)	191 (77.1, 472)	40.6 (-79.0, 841)	0.723	0.490	

[1] Fasting blood levels were analyzed with cLMM, Values were transformed by the natural logarithm before the analyses.
[2] Values are geometric means (1 SD ranges) of fasting blood levels at baseline and follow-up within treatments and periods.
[3] Relative model-adjusted mean change scores (95% CIs) from baseline to follow-up as percentages calculated from the model estimates, % = $(\exp^{estimate} - 1) \times 100$.
[4] P-values for relative changes from baseline to follow-up within treatments and different RBCM LA levels (time effects).
[5] P-values for relative changes from baseline to follow-up within treatments and between high and low RBCM LA levels. The first and second values refer to differences between low and high RBCM LA levels after the n-3 and n-6 interventions, respectively.
[6] P-values for relative changes from baseline to follow-up between treatments and within low and high RBCM LA levels. The first and second values refer to between-treatment differences in low and high n-3 index, respectively.
[7] One influential outlier was excluded from the final analyses of hsCRP, SAAt, SAA1t, SAA2t, S100A8, S100At, Il-8, MIP-1α, MIP-1β, IL-1RA, and adiponectin, and two outliers were excluded from apelin.
Abbreviations: cLMM, constrained linear-mixed effects model; CVR, Covariate Ratio; LA, linoleic acid; RBCMs, red blood cell membranes

Supplemental Table 9. Correlation analysis matrix between changes in fatty acid profile in RBCMs and circulating inflammatory markers after 7 wk supplementation with n-3 or n-6

	EPA		DHA		EPA and DHA		LA		ARA	
	**R[1]**	**95% BCa CI[2]**	**R[1]**	**95% BCa CI[2]**	**R[1]**	**95% BCa CI[2]**	**R[1]**	**95% BCa CI[2]**	**R[1]**	**95% BCa CI[2]**
**hsCRP**										
**n-3**	0.195	−0.044,0.418	0.277	−0.069, 0.473	0.157	−0.090, 0.380	0.053	−0.308, 0.425	−0.052	−0.281,0.159
**n-6**	−0.061	−0.359, 0.330	0.077	−0.264, 0.422	0.022	−0.265, 0.384	0.091	−0.278, 0.391	0.139	−0.258, 0.395
**SAAt[3]**										
**n-3**	−0.016	−0.420, 0.309	−0.164	−0.421, 0.069	−0.089	−0.425, 0.207	−0.129	−0.464, 0.290	0.046	−0.278
**n-6**	−0.011	−0.328, 0.379	0.088	−0.264, 0.414	0.065	−0.193, 0.387	−0.125	−0.386, 0.108	0.130	−0.246, 0.392
**SAA1t[3]**										
**n-3**	0.031	−0.389, 0.343	−0.112	−0.362, 0.116	−0.028	−0.368, 0.241	−0.167	−0.494, 0.247	0.047	−0.278, 0.349
**n-6**	−0.010	−0.329, 0.389	0.111	−0.244, 0.246	0.077	−0.194, 0.399	−0.150	−0.398, 0.081	0.138	−0.236, 0.397
**SAA2t[3]**										
**n-3**	−0.119	−0.477, 0.235	−0.236	−0.526, 0.077	−0.224	−0.532, 0.112	0.056	−0.245, 0.376	0.028	−0.296, 0.316
**n-6**	0.002	−0.303, 0.363	0.023	−0.329, 0.364	0.037	−0.218, 0.355	−0.055	−0.363, 0.181	0.098	−0.290, 0.350
**S100At**										
**n-3**	0.098	−0.148, 0.273	0.096	−0.221, 0.332	0.070	−0.161, 0.228	−0.175	−0.468, 0.244	−0.003	−0.311, 0.231
**n-6**	**−0.336***	−0.609, −0.010	−0.099	−0.417, 0.254	−0.269	−0.535, 0.099	0.203	−0.188, 0.510	**0.411***	0.131, 0.618
**S100A8[3]**										
**n-3**	0.048	−0.174, 0.280	−0.034	−0.321, 0.242	0.007	−0.223, 0.227	−0.002	−0.317, 0.348	−0.092	−0,373, 0.195
**n-6**	**−0.338***	−0.604, −0.014	−0.080	−0.411, 0.282	−0.261	−0.532, 0.112	0.182	−0.202, 0.498	**0.419****	0.132, 0.627
**S100A9**										
**n-3**	0.150	−0.082, 0.351	0.094	−0.185, 0.351	0.104	−0.125, 0.307	−0.085	−0.392, 0.266	−0.102	−0.423, 0.211
**n-6**	−0.293	−0.569, 0.027	−0.107	−0.425, 0.274	−0.247	−0.542, 0,147	0.225	−0.218, 0.538	**0.375***	0.080, 0.589
**Eotaxin**										
**n-3**	−0.109	−0.382, 0.155	−0.050	−0.338, 0.201	−0.032	−0.295, 0.233	−0.120	−0.396, 0.218	0.159	−0.204, 0.516
**n-6**	−0.074	−0.044, 0.306	−0.046	−0.341, 0.253	−0.037	−0.392, 0.321	0.105	−0.204, 0.413	−0.164	−0.547, 0.295
**IL-8**										
**n-3**	−0.258	−0.448, −0.042	−0.163	−0.375, 0.098	−0.171	−0.346, 0.012	−0.162	−0.489, 0.247	**0.419****	0.171, 0.620

**n-6**	0.017	−0.295, 0.254	−0.064	−0.399, 0.257	−0.007	−0.378, 0.295	0.211	−0.033, 0.494	−0.186	−0.451, 0.148
**MCP-1**										
**n-3**	0.265	0.003, 0.534	0.095	−0.216, 0.415	−0.199	−0.095, 0.492	−0.018	−0.403, 0.298	−0.278	−0.512, 0.033
**n-6**	0.183	−0.161, 0.429	0.098	−0.261, 0.405	0.157	−0.234, 0.461	0.037	−0.267, 0.380	**−0.399***	−0.671, 0.060
**MIP-1α**										
**n-3**	0.046	−0.243, 0.333	−0.110	−0.354, 0.145	−0.065	−0.334, 0.222	−0.110	−0.417, 0.160	0.026	−0.291, 0.323
**n-6**	−0.086	−0.357, 0.160	−0.078	−0.331, 0.200	−0.071	−0.357, 0.211	0.208	−0.156, 0.535	−0.163	−0.479, 0.207
**MIP-1β**										
**n-3**	0.045	−0.289, 0.354	0.003	−0.341, 0.265	0.034	−0.331, 0.323	−0.091	−0.373, 0.156	0.092	−0.266, 0.447
**n-6**	0.030	−0.297, 0.348	−0.022	−0.299, 0.424	0.044	−0.280, 0.338	0.224	0.007, 0.479	−0.224	−0.600, 0.233
**RANTES**										
**n-3**	−0.228	−0.517, 0.082	−0.197	−0.381, 0.093	−0.234	−0,483, 0.058	−0.019	−0.334, 0.366	**0.341***	0.078, 0.588
**n-6**	**−0.426****	−0.614, −0.227	−0.109	−0.419, 0.185	−0.311	−0.543, −0.057	−0.218	−0.501, 0.160	**0.458****	0.236, 0.679
**TNF**										
**n-3**	**−0.335***	−0.659, 0.104	−0.305	−0.620, 0.103	**−0.332***	−0.671, 0.139	0.104	−0.140, 0.326	**0.510****	0.188, 0.727
**n-6**	−0.016	−0.330, 0.337	−0.046	−0.317, 0.334	−0.026	−0.329, 0.358	0.168	−0.036, 0.358	−0.074	−0.440, 0.255
**IL-1RA**[3]										
**n-3**	−0.112	−0.503, 0.369	−0.099	−0.334, 0.203	−0.137	−0.438, 0.239	−0.046	−0.434, 0.365	0.043	−0.218, 0.316
**n-6**	−0.174	−0.461, 0.125	0.058	−0.254, 0.380	−0.085	−0.365, 0.218	0.092	−0.219, 0.411	0.199	−0.323, 0.608
**Adiponectin**										
**n-3**	−0.146	−0.451, 0.192	−0.287	−0.551, 0.037	−0.253	−0.522, 0.090	0.103	−0.307, 0.488	0.074	−0.318, 0.144
**n-6**	**0.340***	−0.079, 0.624	0.310	−0.038, 0.572	**0.343***	−0.029, 0.616	0.159	−0.192, 0.480	−0.245	−0.526, 0.108
**Apelin**										
**n-3**	−0.020	−0.429, 0.374	−0.090	−0.420, 0.253	0.005	−0.394, 0.357	0.036	−0.288, 0.307	0.048	−0.266, 0.423
**n-6**	−0.033	−0.384, 0.350	0.036	−0.285, 0.350	−0.020	−0.348, 0.327	0.050	−0.293, 0.370	0.054	−0.179, 0.322

[1] Values are Pearson's rs from bivariate correlation analyses using log_2 fold change.
[2] 95% BCa CI obtained from bootstrapping procedure using 1000 replicates.
[3] One outlier was removed from IL-1RA, SAAt, SAA1t, and SAA2t (n-3).
* Correlation is significant at 0.05**,** ** Correlation is significant at 0.01.
Abbreviations**:** BCa Cl, bias-corrected and accelerated confidence interval; DHA, docosahexaenoic acid; EPA, eicosapentaenoic acid; LA, linoleic acid; ARA, arachidonic acid; RBCMs, red blood cell membranes.

Supplemental Table 10. Correlation analysis matrix between changes in fatty acid profile in serum and circulating inflammatory markers after 7 wk supplementation with n-3 or n-6

	EPA		DHA		EPA and DHA		LA		ARA	
	**R[1]**	**95% BCa CI[2]**	**R[1]**	**95% BCa**	**R[1]**	**95% BCa CI[2]**	**R[1]**	**95% BCa CI[2]**	**R[1]**	**95% BCa CI[2]**
**hsCRP**										
**n-3**	0.123	−0.112, 0.327	0.156	−0.168, 0.415	0.110	−0.149, 0.338	−0.212	−0.527, 0.217	−0.110	−0.346, 0.145
**n-6**	−0.057	−0.270, 0.150	0.053	−0.246, 0.354	0.017	−0.169, 0.192	0.162	−0.255, 0.555	0.056	−0.262, 0.327
**SAAt[3]**										
**n-3**	0.024	−0.435, 0.406	−0.068	−0.492, 0.261	−0.006	−0.450, 0.340	**−0.401****	−0.667, −0.008	−0.072	−0.256, 0.113
**n-6**	0.077	−0.135, 0.355	0.064	−0.301, 0.406	0.099	−0.154, 0.365	0.094	−0.357, 0.458	0.035	−0.302, 0.320
**SAA1t[3]**										
**n-3**	0.076	−0.421, 0.423	−0.008	−0.409, 0.326	0.053	−0.419, 0.404	**−0.385***	−0.649, 0.012	−0.076	−0.253, 0.088
**n-6**	0.068	−0.149, 0.295	0.068	−0.263, 0.378	0.097	−0.138, 0.345	0.082	−0.391, 0.471	0.026	−0.297, 0.297
**SAA2t[3]**										
**n-3**	−0.127	−0.503, 0.199	−0.209	−0.565, 0.179	−0.174	−0.528, 0.164	**−0.330***	−0.594, −0.010	−0.038	−0.261, 0.211
**n-6**	0.120	−0.095, 0.366	0.057	−0.295, 0.385	0.116	−0.172, 0.411	0.125	−0.359, 0.531	0.086	−0.196, 0.344
**S100At**										
**n-3**	0.057	−0.208, 0.268	0.144	−0.125, 0.351	0.077	−0.168, 0.266	−0.215	−0.560, 0.345	−0.135	−0.444, 0.104
**n-6**	−0.178	−0.453, 0.146	0.005	−0.334, 0.403	−0.090	−0.384, 0.275	0.221	−0.076, 0.460	0.129	−0.104, 0.357
**S100A8[3]**										
**n-3**	−0.028	−0.292, 0.234	−0.000	−0.272, 0.229	−0.013	−0.276, 0.233	0.049	−0.257, 0.352	−0.220	−0.576, 0.161
**n-6**	−0.186	−0.413, 0.081	0.006	−0.296, 0.343	−0.094	−0.383, 0.208	0.217	−0.043, 0.419	0.127	−0.096, 0.378
**S100A9**										
**n-3**	0.132	−0.129, 0.372	0.139	−0.125, 0.358	0.124	−0.120, 0.373	−0.078	−0.459, 0.334	−0.051	−0.451, 0.309
**n-6**	−0.115	−0.388, 0.194	0.023	−0.289, 0.368	−0.046	−0.363, 0.264	0.214	−0.168, 0.489	0.126	−0.120, 0.410
**Eotaxin**										
**n-3**	−0.067	−0.354, 0.205	0.042	−0.220, 0.276	0.018	−0.271, 0.279	0.021	−0.329, 0.369	0.285	−0.022, 0.510
**n-6**	−0.174	−0.431, 0.117	−0.161	−0.402, 0.092	−0.172	−0.410, 0.116	−0.029	−0.349, 0.317	−0.308	−0.615, 0.090
**IL-8**										
**n-3**	−0.147	−0.408, 0.128	−0.031	−0.282, 0.232	−0.050	−0.334, 0.233	−0.059	−0.411, 0.288	**0.417****	0.118, 0.638

**n-6**	−0.034	−0.305, 0.273	−0.114	−0.359, 0.236	−0.076	−0.315, 0.235	0.127	−0.096, 0.388	−0.208	−0.501, 0.087
**MCP-1**										
**n-3**	0.258	−0.023, 0.542	0.174	−0.156, 0.524	0.227	−0.075, 0.526	0.057	−0.379, 0.423	−0.201	−0.510, 0.096
**n-6**	0.077	−0.186, 0.346	−0.061	−0.297, 0.134	−0.006	−0.252, 0.200	−0.095	−0.428, 0.268	**−0.536****	−0.750, −0.153
**MIP-1α**										
**n-3**	0.031	−0.275, 0.323	−0.056	−0.327, 0.228	−0.006	−0.281, 0.238	−0.265	−0.527, 0.019	−0.014	−0.293, 0.233
**n-6**	−0.031	−0.251, 0.179	−0.146	−0.361, 0.068	−0.081	−0.311, 0.153	0.027	−0.253, 0.289	**−0.502****	−0.694, −0.277
**MIP-1β**										
**n-3**	0.064	−0.227, 0.336	0.102	−0.217, 0.381	0.093	−0.216, 0.360	−0.163	−0.423, 0.073	0.211	−0.087, 0.443
**n-6**	0.089	−0.132, 0.277	−0.021	−0.274, 0.182	0.049	−0.168, 0.222	0.101	−0.201, 0.387	**−0.455****	−0.761, 0.114
**RANTES**										
**n-3**	−0.202	−0.534, 0.240	−0.084	−0.352, 0.230	−0.149	−0.499, 0.264	−0.077	−0.395, 0.368	**0.417****	0.165, 0.606
**n-6**	−0.312	−0.545, −0.029	−0.212	−0.428, 0.071	−0.285	−0.498, −0.034	−0.190	−0.479, 0.124	0.016	0.255, 0.273
**TNF**										
**n-3**	−0.251	−0.571, 0.200	−0.216	−0.529, 0.174	−0.247	−0.546, 0.167	−0.009	−0.306, 0.221	0.306	0.024, 0.553
**n-6**	−0.038	−0.275, 0.215	−0.126	−0.397, 0.204	−0.086	−0.334, 0.225	0.173	−0.088, 0.412	−0.183	−0.546, 0.177
**IL-1RA**[3]										
**n-3**	−0.243	−0.602, 0.228	−0.177	−0.546, 0.335	−0.238	−0.581, 0.321	−0.084	−0.362, 0.216	0.068	−0.299, 0.465
**n-6**	−0.258	−0.516, 0.034	−0.066	−0.335, 0.227	−0.175	−0.403, 0.050	0.134	−0.141, 0.381	−0.154	−0.548, 0.302
**Adiponectin**										
**n-3**	−0.037	−0.407, 0.304	−0.144	−0.465, 0.221	−0.086	−0.430, 0.291	−0.207	−0.600, 0.235	−0.044	−0.350, 0.254
**n-6**	**0.382***	−0.092, 0.692	**0.363***	0.026, 0.622	**0.394***	0.028, 0.670	**0.329***	0.012, 0.570	−0.090	−0.285, 0.153
**Apelin**										
**n-3**	0.039	−0.420, 0.491	−0.062	−0.422, 0.269	0.035	−0.341, 0.493	0.065	−0.316, 0.407	0.299	−0.059, 0.545
**n-6**	0.069	−0.212, 0.322	0.142	−0.149, 0.423	0.105	−0.159, 0.378	0.052	−0.353, 0.402	0.038	−0.187, 0.263

[1] Values are Pearson's rs from bivariate correlation analyses using $\log_2$ fold change.
[2] 95% BCa CI obtained from bootstrapping procedure using 1000 replicates.
[3] One outlier was removed from IL-1RA, SAAt, SAA1t, and SAA2t (n-3).
* Correlation is significant at 0.05**,** ** Correlation is significant at 0.01.
Abbreviations**:** BCa Cl, bias-corrected and accelerated confidence interval; DHA, docosahexaenoic acid; EPA, eicosapentaenoic acid; LA, linoleic acid; ARA, arachidonic acid.

Supplemental Table 11. Period-specific responses in relative changes in endothelial function, blood pressure, and pulse markers after 7 wk of supplementation with n-3 or n-6 PUFAs[1]

Variable and group	Baseline[2]	Follow-up[2]	Relative change[3]	Time[4]	Time × prd[5]	Group × prd[6]
**VRI, index**						
**n-3 : P1**	1.55 (1.26, 1.89)	1.47 (1.00, 2.16)	-5.00 (-24.1, 18.8)	0.650	0.277	0.813
**n-3 : P2**	1.22 (0.73, 2.01)	1.42 (0.90, 2.25)	16.7 (-13.4, 57.3)	0.308		0.199
**n-6 : P1**	1.57 (1.20, 2.06)	1.44 (0.90, 2.31)	-8.55 (-27.0, 14.5)	0.433	0.871	
**n-6 : P2**	1.34 (0.94, 1.92)	1.19 (0.62, 2.29)	-11.3 (-34.1, 19.3)	0.425		
**Systolic BP, mmHg**						
**n-3 : P1**	114 (102, 127)	115 (103, 130)	1.19 (-2.62, 5.14)	0.544	0.245	0.332
**n-3 : P2**	119 (107, 132)	116 (105, 129)	-2.02 (-5.75, 1.86)	0.301		0.845
**n-6 : P1**	119 (106, 134)	124 (113, 135)	3.92 (0.010, 7.99)	0.049	0.055	
**n-6 : P2**	120 (105, 137)	118 (107, 131)	-1.48 (-5.23, 2.42)	0.447		
**Diastolic BP,**						
**n-3 : P1**	73.3 (63.2, 84.9)	71.9 (61.2, 84.5)	-1.89 (-6.72, 3.19)	0.455	0.139	0.557
**n-3 : P2**	72.8 (64.1, 82.6)	75.4 (67.4, 84.3)	3.58 (-1.59, 9.03)	0.176		0.187
**n-6 : P1**	74.4 (64.9, 85.3)	71.5 (63.1, 80.9)	-3.96 (-8.69, 1.02)	0.116	0.460	
**n-6 : P2**	76.2 (66.5, 87.2)	75.1 (63.7, 88.7)	-1.33 (-6.26, 3.87)	0.607		
**Pulse, beats/min**						
**n-3 : P1**	57.3 (50.9, 64.5)	58.4 (51.5, 66.3)	1.96 (-1.65, 5.70)	0.289	0.588	0.992
**n-3 : P2**	60.9 (51.8, 71.5)	63.0 (53.7, 74.0)	3.54 (-0.81, 8.08)	0.111		0.975
**n-6 : P1**	60.5 (52.5, 69.8)	61.7 (53.0, 71.8)	1.99 (-2.85, 7.07)	0.423	0.727	
**n-6 : P2**	60.9 (54.2, 68.4)	63.0 (53.4, 74.2)	3.41 (-2.79, 10.0)	0.284		

[1] Fasting blood levels were analyzed with cLMM. Values were transformed by the natural logarithm before the analyses.

[2] Values are geometric means (1 SD ranges) of fasting blood levels at baseline and follow-up within treatments and periods.

[3] Relative model-adjusted mean change scores (95% CIs) from baseline to follow-up as percentages calculated from the model estimates, % = $(\exp^{\text{estimate}} - 1) \times 100$.

[4] P-values for relative changes from baseline to follow-up within treatments and periods (time effects).

[5] P-values for relative changes from baseline to follow-up within treatments and between periods (period differences in time effects), The first and second values refer to between-period differences after the n-3 and n-6 interventions, respectively.

[6] P-values for relative changes from baseline to follow-up between treatments and within periods (group differences in time effects within each stratum of period), The first and second values refer to between-treatment differences in P1 and P2, respectively.

Abbreviations: BP, blood pressure, CI, confidence interval; cLMM, constrained linear-mixed effects model; P1, period 1; P2, period 2; prd, period; VRI, vascular reaction index.

Supplemental Table 12. Correlation analysis matrix between changes in blood pressure, pulse and VRI and inflammatory markers after 7 wk supplementation with n-3 or n-6

	**Systolic BP**		**Diastolic BP**		**Pulse**		**VRI**	
	**R[1]**	**95% BCa CI[2]**	**R[1]**	**95% BCa CI[2]**	**R[1]**	**95% BCa CI[2]**	**R[1]**	**95% BCa CI[2]**
**hsCRP**								
**n-3**	0.082	−0.286, 0.453	0.068	−0.390, 0.463	0.191	−0.087, 0.503	0.078	−0.316, 0.454
**n-6**	−0.295	−0.492, −0.063	−0.015	−0.327, 0.252	0.224	−0.068, 0.482	0.062	−0.243, 0.338
**SAAt[3]**								
**n-3**	0.058	−0.229, 0.375	−0.262	−0.589, 0.168	**0.437****	0.184, 0.646	−0.297	−0.575, 0.039
**n-6**	−0.180	−0.411, 0.127	0.047	−0.210, 0.276	0.122	−0.141, 0.390	0.038	−0.227, 0.324
**SAA1t[3]**								
**n-3**	0.085	−0.189, 0.410	−0,299	−0.616, 0.137	**0.440****	0.180, 0.643	−0.306	−0,583, 0.010
**n-6**	−0.179	−0.401, 0.122	0.065	−0.186, 0.300	0.121	−0.140, 0.394	0.043	−0.227, 0.331
**SAA2t[3]**								
**n-3**	−0,047	−0.413, 0.316	−0,110	−0.388, 0.023	**0.365***	0.111, 0.604	−0,200	−0.467, 0.145
**n-6**	−0.179	−0.410, 0.113	−0.024	−0.341, 0.240	0.133	−0.148, 0.402	0.032	−0.239, 0.333
**S100At**								
**n-3**	0.056	−0.300, 0,416	−0.015	−0.270, 0.267	0.140	−0.193, 0.458	−0.153	−0.454, 0.178
**n-6**	−0.074	−0.303, 0.176	0.052	−0.262, 0.318	0.232	−0.146, 0.488	−0.101	−0.388, 0.239
**S100A8[3]**								
**n-3**	0.080	−0.335, 0.476	0.025	−0.257, 0.287	0.176	−0.198, 0.523	−0.149	−0.457, 0.144
**n-6**	−0.075	−0.299, 0.166	0.048	−0.267, 0.316	0.239	−0.115, 0.484	−0.078	−0.381, 0.282
**S100A9**								
**n-3**	−0.118	−0.437, 0.262	0.095	−0.193, 0.363	0.012	−0.307, 0.324	−0.070	−0.317, 0.208
**n-6**	−0.049	−0.283, 0.219	0.085	−0.244, 0.353	0.295	−0.110, 0.557	−0.161	−0.382, 0.104
**Eotaxin**								
**n-3**	0.092	−0.182, 0.355	−0.156	−0.427, 0.114	−0.024	−0.400, 0.339	−0.162	−0.406, 0.143
**n-6**	0.274	−0.096, 0.548	−0.136	−0.469, 0.282	−0.205	−0.522, 0.161	0.030	−0.175, 0.194
**IL-8**								
**n-3**	0.089	−0.237, 0.439	0.196	−0.160, 0.523	−0.083	−0.411, 0.288	−0.021	−0.286, 0.263
**n-6**	0.281	−0.046, 0.499	−0.030	−0.296, 0.291	0.199	−0.041, 0.466	0.118	−0.110, 0.309

**MCP-1**								
**n-3**	0.281	−0.030, 0.557	−0.274	−0.577, 0.118	0.235	−0.021, 0.471	−0.071	−0.475, 0.313
**n-6**	**0.479****[**]**	0.143, 0.702	−0.094	−0.321, 0.134	−0.008	−0.212, 0.203	0.073	−0.085, 0.271
**MIP-1α**								
**n-3**	0.040	−0.268, 0.390	−0.083	−0.342, 0.200	**0.407***	0.123, 0.605	**−0.351***	−0.564, −0.061
**n-6**	0.177	−0.130, 0.442	−0.035	−0.303, 0.254	0.097	−0.198, 0.369	−0.193	−0.446 0.150
**MIP-1β**								
**n-3**	−0.194	−0.489, 0.191	−0.172	−0.387, 0.101	0.036	−0.359, 0.538	−0.301	−0.572, 0.063
**n-6**	0.128	−0.196, 0.388	0.105	−0.123, 0.351	0.225	−0.065, 0.488	−0.164	−0.370, 0.087
**RANTES**								
**n-3**	0.062	−0.269, 0.282	−0.061	−0.447, 0.420	0.123	−0.152, 0.351	−0.011	−0.254, 0.271
**n-6**	0.007	−0.393, 0.458	0.216	−0.174, 0.580	0.069	−0.221, 0.350	−0.211	−0.536, 0.133
**TNF**								
**n-3**	−0.045	−0.353, 0.237	−0.065	−0.311, 0.174	−0.119	−0.508,0.329	**−0.440***	−0.705, −0.118
**n-6**	0.184	−0.147, 0.429	0.290	−0.067, 0.560	0.091	−0.139, 0.303	−0.051	−0.208, 0.142
**IL-1RA**[3]								
**n-3**	−0.054	−0.432, 0.282	0.308	−0.093, 0.590	0.205	−0.122, 0.511	0.009	−0.323, 0.304
**n-6**	0.128	−0.167, 0.385	0.062	−0.233, 0.310	0.055	−0.213, 0.256	−0.170	−0.449, 0.152

[1] Values are Pearson’s rs from bivariate correlation analyses using log_2 fold change,

[2] 95% BCa CI obtained from bootstrapping procedure using 1000 replicates.

[3] One outlier was removed from IL-1RA, SAAt, SAA1tm, and SAA2t (n-3)

* Correlation is significant at 0.05

** Correlation is significant at 0.01

Abbreviations: BCa CI, bias-corrected and accelerated confidence interval; BP, blood pressure; VRI, vascular reaction index

Supplemental Table 13. Relative changes in subcutaneous adipose tissue fatty acid levels after seven wk of supplementation with n-3 or n-6 PUFAs [1]

Variable and	Baseline (n = 39)[2]	Follow-up (n =38)[2]	Relative change[3]	Time[4]	Tx × time[5]
**Total fatty acids**					
**PUFA n-3, wt%**					<0.001
n-3	2.17 (1.78, 2.65)	2.36 (1.94, 2.88)	8.60 (6.06, 11.2)	<0.001	
n-6	2.31 (1.92, 2.77)	2.26 (1.90, 2.70)	-1.67 (-4.03, 0.74)	0.167	
**PUFA n-6, wt%**					<0.001
n-3	13.1 (11.9, 14.4)	13.1 (11.9, 14.4)	-0.18 (-1.01, 0.65)	0.664	
n-6	13.3 (12.2, 14.5)	13.9 (12.8, 15.1)	4.56 (3.67, 5.46)	<0.001	
**PUFA n-9, wt%**					0.382
n-3	0.021 (0.013, 0.035)	0.020 (0.012, 0.032)	-3.85 (-9.36, 1.99)	0.185	
n-6	0.019 (0.014, 0.026)	0.019 (0.014, 0.025)	-0.70 (-5.42, 4.26)	0.773	
**MUFA, wt%**					0.888
n-3	54.5 (50.9, 58.3)	54.1 (50.7, 57.7)	-0.72 (-1.17, -0.28)	0.002	
n-6	54.4 (52.0, 56.9)	54.1 (51.2, 57.0)	-0.66 (-1.39, 0.067)	0.074	
**SFA, wt%**					0.040
n-3	29.4 (26.5, 32.6)	29.7 (27.0, 32.6)	0.86 (-0.29, 2.03)	0.138	
n-6	29.2 (26.3, 32.3)	28.9 (25.7, 32.5)	-0.86 (-2.03, 0.32)	0.147	
**Trans FA, wt%**					0.021
n-3	0.47 (0.38, 0.59)	0.48 (0.39, 0.59)	1.65 (-0.44, 3.79)	0.119	
n-6	0.49 (0.40, 0.61)	0.48 (0.38, 0.61)	-1.87 (-3.94, 0.25)	0.082	
**Omega-3 (n-3)**					
**C18:3 n-3, ALA, wt%**					0.090
n-3	1.13 (0.95, 1.34)	1.15 (0.95, 1.38)	1.65 (-0.74, 4.10)	0.170	
n-6	1.24 (0.98, 1.55)	1.22 (0.97, 1.53)	-1.28 (-3.66, 1.16)	0.291	
**C20:4 n-3, ETA, wt%**					0.005
n-3	0.092 (0.074, 0.11)	0.097 (0.077, 0.12)	5.29 (2.03, 8.65)	0.002	
n-6	0.097 (0.071, 0.13)	0.096 (0.071, 0.13)	-1.41 (-4.55, 1.82)	0.377	
**C20:5 n-3, EPA, wt%**					<0.001
n-3	0.15 (0.11, 0.21)	0.23 (0.17, 0.31)	50.2 (40.8, 60.2)	<0.001	
n-6	0.16 (0.12, 0.20)	0.16 (0.12, 0.20)	-1.38 (-7.71, 5.38)	0.673	
**C21:5 n-3, Heneicosapentaenoic acid, wt%**					<0.001
n-3	0.008 (0.004, 0.016)	0.012 (0.007, 0.021)	44.3 (28.8, 61.6)	<0.001	
n-6	0.009 (0.005, 0.016)	0.008 (0.005, 0.014)	-2.20 (-12.9, 9.83)	0.699	
**C22:5 n-3, DPA, wt%**					<0.001
n-3	0.38 (0.28, 0.50)	0.41 (0.31, 0.52)	7.36 (4.63, 10.2)	<0.001	
n-6	0.38 (0.28, 0.51)	0.37 (0.27, 0.50)	-2.00 (-4.96, 1.05)	0.189	
**C22:6 n-3, DHA, wt%**					<0.001
n-3	0.36 (0.22, 0.60)	0.42 (0.27, 0.65)	16.0 (11.0, 21.3)	<0.001	
n-6	0.37 (0.24, 0.56)	0.35 (0.23, 0.53)	-3.67 (-7.38, 0.18)	0.061	
**Omega-6 (n-6)**					
**C18:2 n-6, LA, wt%**					<0.001
n-3	11.9 (10.6, 13.3)	11.9 (10.6, 13.3)	-0.033 (-0.56, 0.50)	0.899	
n-6	12.2 (11.1, 13.3)	12.8 (11.7, 13.9)	4.91 (3.71, 6.12)	<0.001	
**C18:3 n-6, GLA, wt**					0.113
n-3	0.058 (0.046, 0.073)	0.058 (0.046, 0.073)	-0.94 (-7.04, 5.57)	0.766	
n-6	0.057 (0.044, 0.073)	0.060 (0.049, 0.074)	6.23 (-0.48, 13.4)	0.068	

**C20:2 n-6, Eicosadienoic acid, wt%**					0.051
n-3	0.20 (0.17, 0.23)	0.20 (0.17, 0.22)	-2.32 (-4.71, 0.13)	0.063	
n-6	0.20 (0.17, 0.23)	0.20 (0.17, 0.24)	1.16 (-1.38, 3.77)	0.362	
**C20:3 n-6, DGLA, wt%,**					0.372
n-3	0.24 (0.18, 0.32)	0.24 (0.17, 0.32)	-0.85 (-4.19, 2.60)	0.616	
n-6	0.25 (0.18, 0.34)	0.25 (0.18, 0.34)	1.34 (-2.16, 4.97)	0.448	
**C20:4 n-6, ARA, wt%**					0.296
n-3	0.45 (0.32, 0.63)	0.45 (0.32, 0.62)	-0.56 (-2.76, 1.69)	0.614	
n-6	0.44 (0.34, 0.56)	0.44 (0.35, 0.56)	1.52 (-1.85, 5.01)	0.370	
**C22:2 n-6, Docosadienoic acid, wt%**					0.974
n-3	0.007 (0.006, 0.008)	0.007 (0.006, 0.008)	-0.19 (-5.85, 5.81)	0.947	
n-6	0.006 (0.005, 0.007)	0.006 (0.005, 0.007)	-0.32 (-6.11, 5.82)	0.914	
**C22:4 n-6, Adrenic acid, wt%**					0.370
n-3	0.16 (0.11, 0.24)	0.16 (0.11, 0.22)	-2.91 (-5.72, -0.017)	0.049	
n-6	0.16 (0.11, 0.21)	0.15 (0.11, 0.21)	-0.99 (-4.32, 2.46)	0.558	
**C22:5 n-6, Docosapentaenoic acid, wt%**					0.028
n-3	0.032 (0.024, 0.043)	0.033 (0.025, 0.044)	2.78 (-0.30, 5.95)	0.076	
n-6	0.030 (0.023, 0.040)	0.029 (0.022, 0.037)	-1.83 (-4.84, 1.27)	0.235	
**Omega-7 (n-7)**					
**C16:1 n-7, Palmitoleic acid, wt%**					0.947
n-3	3.66 (2.69, 4.99)	3.59 (2.62, 4.92)	-1.63 (-6.12, 3.08)	0.481	
n-6	3.43 (2.53, 4.65)	3.37 (2.53, 4.50)	-1.84 (-6.44, 2.99)	0.437	
**C18:1 n-7, Cis-vaccenic acid, wt%**					0.877
n-3	1.97 (1.72, 2.26)	1.96 (1.73, 2.23)	-0.64 (-2.03, 0.78)	0.368	
n-6	2.04 (1.81, 2.31)	2.03 (1.79, 2.31)	-0.50 (-1.62, 0.64)	0.378	
C20:**1 n-7, Paullinic acid, wt%**					0.866
n-3	0.042 (0.037, 0.048)	0.042 (0.037, 0.048)	0.31 (-2.08, 2.76)	0.795	
n-6	0.039 (0.032, 0.047)	0.039 (0.032, 0.048)	0.66 (-2.78, 4.22)	0.702	
**C22:1 n-7, Cis-15-docosenoic acid, wt%**					0.096
n-3	0.002 (0.001, 0.003)	0.002 (0.001, 0.003)	8.81 (-4.20, 23.6)	0.187	
n-6	0.002 (0.001, 0.003)	0.002 (0.001, 0.003)	-6.25 (-17.7, 6.83)	0.323	
**Omega-9 (n-9)**					
**C16:1 n-9, Cis-7-hexadecenoic acid, wt%**					0.491
n-3	0.56 (0.50, 0.63)	0.56 (0.49, 0.63)	-1.13 (-3.43, 1.23)	0.334	
n-6	0.57 (0.50, 0.64)	0.57 (0.49, 0.65)	0.022 (-2.37, 2.47)	0.985	
**C18:1 n-9, Oleic acid, wt%**					0.720
n-3	46.5 (43.8, 49.3)	46.2 (43.7, 48.8)	-0.65 (-1.01, -0.29)	0.001	
n-6	46.6 (44.7, 48.6)	46.4 (44.2, 48.6)	-0.54 (-1.03, -0.052)	0.031	
**C20:1 n-9, Gondoic acid, wt%**					0.766
n-3	0.73 (0.61, 0.86)	0.71 (0.60, 0.85)	-1.72 (-4.36, 1.01)	0.207	
n-6	0.71 (0.59, 0.85)	0.70 (0.57, 0.87)	-1.15 (-3.88, 1.67)	0.410	
**C20:3 n-9, Mead acid, wt%**					0.382
n-3	0.021 (0.013, 0.035)	0.020 (0.012, 0.032)	-3.85 (-9.36, 1.99)	0.185	
n-6	0.019 (0.014, 0.026)	0.019 (0.014, 0.025)	-0.70 (-5.42, 4.26)	0.773	
**C22:1 n-9, Erucic acid, wt%**					0.807
n-3	0.044 (0.032, 0.061)	0.044 (0.031, 0.062)	-0.62 (-4.48, 3.40)	0.753	
n-6	0.040 (0.029, 0.055)	0.040 (0.029, 0.056)	0.072 (-3.92, 4.23)	0.972	
**C24:1 n-9, Nervonic, wt%**					0.789
n-3	0.015 (0.011, 0.021)	0.015 (0.010, 0.022)	-0.84 (-6.84, 5.54)	0.785	

n-6	0.013 (0.009, 0.019)	0.013 (0.009, 0.019)	0.34 (-5.89, 6.99)	0.915	
**Omega 11 (n-11)**					
**C20:1 n-11, Gadoleic acid, wt%**					0.738
n-3	0.15 (0.11, 0.21)	0.15 (0.11, 0.21)	-0.62 (-3.97, 2.85)	0.716	
n-6	0.16 (0.11, 0.22)	0.16 (0.11, 0.22)	-1.43 (-4.84, 2.11)	0.414	
**Saturated fatty acids**					
**C14:0, Myristic acid, wt%**					0.007
n-3	2.86 (2.50, 3.28)	2.90 (2.56, 3.28)	1.17 (0.005, 2.36)	0.049	
n-6	2.73 (2.24, 3.33)	2.70 (2.18, 3.34)	-1.29 (-2.57, 0.016)	0.053	
**C15:0, Pentadecanoic acid, wt%**					0.110
n-3	0.30 (0.26, 0.36)	0.31 (0.26, 0.36)	0.86 (-0.18, 1.92)	0.102	
n-6	0.31 (0.25, 0.38)	0.31 (0.25, 0.38)	-0.27 (-1.23, 0.70)	0.571	
**C16:0, Palmitic acid, wt%**					0.106
n-3	20.2 (18.3, 22.2)	20.3 (18.5, 22.2)	0.53 (-0.40, 1.46)	0.257	
n-6	20.2 (18.2, 22.3)	20.1 (18.0, 22.4)	-0.55 (-1.49, 0.40)	0.244	
**C17:0, Heptadecanoic acid, wt%**					0.074
n-3	0.22 (0.18, 0.28)	0.23 (0.18, 0.28)	1.82 (-0.20, 3.88)	0.076	
n-6	0.24 (0.20, 0.29)	0.23 (0.19, 0.29)	-0.77 (-2.79, 1.30)	0.453	
**C18:0, Stearic acid, wt%**					0.132
n-3	4.25 (3.24, 5.56)	4.34 (3.31, 5.68)	2.08 (-1.52, 5.81)	0.253	
n-6	4.18 (3.24, 5.38)	4.10 (3.13, 5.38)	-1.82 (-5.38, 1.86)	0.318	
**C20:0, Arachidic acid, wt%**					0.669
n-3	0.15 (0.11, 0.20)	0.15 (0.11, 0.20)	0.91 (-3.22, 5.22)	0.662	
n-6	0.13 (0.096, 0.17)	0.13 (0.096, 0.16)	-0.36 (-4.55, 4.02)	0.865	
**C22:0, Behenic acid, wt%**					0.504
n-3	0.030 (0.019, 0.047)	0.030 (0.018, 0.049)	-0.52 (-5.31, 4.50)	0.830	
n-6	0.026 (0.018, 0.037)	0.027 (0.020, 0.037)	1.86 (-3.24, 7.24)	0.471	
**C23:0, Tricosylic acid, wt%**					0.962
n-3	0.005 (0.004, 0.006)	0.005 (0.004, 0.007)	-6.40 (-16.4, 4.83)	0.244	
n-6	0.004 (0.003, 0.005)	0.004 (0.003, 0.005)	-6.05 (-16.4, 5.52)	0.283	
**C24:0, Lignoceric acid, wt%**					0.379
n-3	0.018 (0.012, 0.027)	0.018 (0.011, 0.028)	-0.92 (-7.92, 6.61)	0.799	
n-6	0.014 (0.010, 0.020)	0.015 (0.011, 0.020)	3.16 (-2.96, 9.67)	0.309	

[1] Pooled data of fasting adipose tissue fatty acid levels (measured as wt%) were analyzed with cLMM adjusted for the main effect of period and subject-averaged baselines. Values were transformed by the natural logarithm before the analyses.
[2] Values are geometric means (1 SD ranges) of fasting levels at baseline and follow-up.
[3] Relative changes from baseline to follow-up as percentages (95% CIs) calculated from cLMM estimates: % = $(\exp^{estimate} - 1) \times 100$,
[4] P-values (from cLMMs) for relative changes from baseline to follow-up within treatments (time effects).
[5] P-values (from cLMMs) for relative changes from baseline to follow-up between treatments (group differences in time effects).
Abbreviations: ALA, alpha-linolenic acid; ARA, arachidonic acid; cLMM, constrained linear-mixed effects model; DHA, docosahexaenoic acid; DGLA, dihomo-gamma-linolenic acid; DPA, docosapentaenoic acid; EPA, eicosapentaenoic acid; ETA, eicosatetraenoic acid; GLA, gamma-linolenic acid; n-3, omega-3 PUFAs; n-6, omega-6 PUFAs; PUFAs, polyunsaturated fatty acids; RBCMs, red blood cell membranes; Tx, treatment; wt%, weight percentage of total fatty acids.

Supplemental Table 14. Correlations between fasting fatty acid levels in SAT and RBCMs[1]

	Baseline[2]		Follow-up[3]		Relative change[3]	
**Variable and group**	**Pearson's *r***	**P-value**	**Pearson's *r***	**P-value**	**Pearson's *r***	**P-value**
**n-3 index, wt%**	0.81 (0.62, 0.89)	<0.001	0.71 (0.46, 0.84)	<0.001	0.76 (0.62, 0.84)	<0.001
**n-3**	0.84 (0.66, 0.93)	<0.001	0.82 (0.62, 0.88)	<0.001	0.11 (-0.19, 0.55)	0.658
**n-6**	0.75 (0.26, 0.92)	<0.001	0.87 (0.53, 0.95)	<0.001	-0.16 (-0.58, 0.19)	0.530
**ALA, wt%**						
**n-3**						
**n-6**						
**EPA, wt%**	0.72 (0.56, 0.82)	<0.001	0.77 (0.61, 0.87)	<0.001	0.83 (0.71, 0.89)	<0.001
**n-3**	0.79 (0.57, 0.88)	<0.001	0.75 (0.57, 0.85)	<0.001	0.17 (-0.29, 0.56)	0.511
**n-6**	0.62 (0.21, 0.84)	0.008	0.81 (0.58, 0.90)	<0.001	0.061 (-0.49, 0.53)	0.816
**DPA, wt%**	0.20 (-0.12, 0.49)	0.242	0.32 (-0.035, 0.57)	0.053	0.57 (0.34, 0.71)	<0.001
**n-3**	0.45 (-0.21, 0.78)	0.062	0.63 (0.10, 0.89)	0.005	0.010 (-0.40, 0.52)	0.969
**n-6**	0.038 (-0.34, 0.45)	0.886	0.16 (-0.28, 0.54)	0.549	-0.087 (-0.47, 0.43)	0.741
**DHA, wt%**	0.80 (0.61, 0.89)	<0.001	0.74 (0.47, 0.86)	<0.001	0.60 (0.43, 0.74)	<0.001
**n-3**	0.84 (0.64, 0.93)	<0.001	0.76 (0.51, 0.86)	<0.001	0.19 (-0.19, 0.59)	0.443
**n-6**	0.73 (0.16, 0.90)	0.001	0.84 (0.44, 0.95)	<0.001	-0.32 (-0.64, 0.038)	0.214
**LA, wt%**	0.38 (0.085, 0.65)	0.021	0.44 (0.13, 0.64)	0.007	0.79 (0.67, 0.85)	<0.001
**n-3**	0.46 (-0.14, 0.75)	0.056	0.39 (-0.11, 0.65)	0.114	0.22 (-0.32, 0.61)	0.387
**n-6**	0.32 (-0.23, 0.74)	0.207	0.17 (-0.18, 0.55)	0.521	0.18 (-0.39, 0.66)	0.495
**GLA, wt%**	0.33 (-0.076, 0.63)	0.052	0.34 (0.022, 0.59)	0.041	0.35 (0.074, 0.56)	0.037
**n-3**	0.49 (0.004, 0.82)	0.040	0.43 (-0.046, 0.74)	0.072	0.46 (0.21, 0.68)	0.056
**n-6**	0.14 (-0.45, 0.73)	0.581	0.29 (-0.20, 0.59)	0.262	-0.015 (-0.39, 0.42)	0.954
**DGLA, wt%**	0.56 (0.34, 0.70)	<0.001	0.41 (0.11, 0.62)	0.012	0.16 (-0.13, 0.43)	0.357
**n-3**	0.51 (0.069, 0.73)	0.029	0.48 (0.096, 0.78)	0.042	-0.097 (-0.66, 0.55)	0.702
**n-6**	0.61 (0.29, 0.79)	0.009	0.48 (0.18, 0.70)	0.051	0.26 (-0.25, 0.59)	0.312
**ARA, wt%**	0.51 (0.11, 0.76)	0.002	0.37 (-0.048, 0.66)	0.025	0.23 (-0.15, 0.51)	0.178
**n-3**	0.64 (-0.12, 0.86)	0.004	0.59 (0.043, 0.80)	0.011	0.28 (-0.15, 0.74)	0.260
**n-6**	0.31 (-0.26, 0.84)	0.234	0.29 (-0.32, 0.77)	0.262	0.039 (-0.39, 0.62)	0.883

[1] Values are Pearson's correlation coefficients (95% BCa CIs) from bivariate correlation analyses using fasting fatty acid levels (measured as wt%) in SAT and RBCMs, Data were log-transformed before the analyses, The n-6/n-3 ratio was calculated from the total levels of n-6 and n-3 PUFAs, The n-3 index is the total sum of EPA and DHA measured in RBCMs (wt%),
[2] Correlations are shown for all participants at the first baseline visit (B1) before any intervention (grey cells) and for pooled period data (B1 and B2) within groups (white cells).
[3] Correlations are shown for pooled period data for all participants (grey cells) and pooled period data within treatment groups (white cells) for follow-up scores and for change scores measured as additive, symmetric percentages (sympercents; see main text).
Abbreviations: ARA, arachidonic acid; ALA, α-linolenic acid; DGLA, dihomo-γ-linolenic acid; DHA, docosahexaenoic acid; DPA, docosapentaenoic acid; EPA, eicosapentaenoic acid; GLA, γ-linolenic acid; LA,

linoleic acid; n-3, omega-3 PUFAs; n-6, omega-6 PUFAs; RBCMs, red blood cell membranes; SAT, subcutaneous adipose tissue; wt%, weight percentage of total fatty acids; 95% BCa CI, 95% bootstrapped (bias-corrected and accelerated, BCa) confidence interval.

Supplemental Table 15. Correlation analysis matrix between changes in fatty acid profile in subcutaneous adipose tissue (SAT) and circulating inflammatory markers after 7 wk supplementation with n-3 or n-6

	**EPA**		**DHA**		**EPA and DHA**		**LA**		**ARA**	
	**R[1]**	**95% BCa CI[2]**	**R[1]**	**95% BCa CI[2]**	**R[1]**	**95% BCa CI[2]**	**R[1]**	**95% BCa CI[2]**	**R[1]**	**95% BCa CI[2]**
**hsCRP**										
**n-3**	−0.275	−0.598, 0.127	−0.021	−0.493, 0.453	−0.136	−0.513, 0.290	0.024	−0.478, 0.534	0.269	−0.179, 0.590
**n-6**	−0.122	−0.571, 0.286	0.394	−0.073, 0.818	0.260	−0.235, 0.633	−0.058	−0.465, 0.329	−0.017	−0.420, 0.321
**SAAt**										
**n-3**	−0.078	−0.342, 0.23	0.042	−0.386, 0.523	−0.022	−0.344, 0.365	−0.022	−0.476, 0.736	0.169	−0.188, 0.376
**n-6**	−0.024	−0.449, 0.328	0.348	−0.176, 0.717	0.272	−0.435, 0.687	−0.197	−0.390, −0.056	−0.025	−0.381, 0.323
**SAA1t**										
**n-3**	−0.121	−0.386, 0.171	0.004	−0.452, 0.461	−0.067	−0.419, 0.327	−0.071	−0.509, 0.705	0.142	−0.235, 0.356
**n-6**	−0.036	−0.429, 0.294	0.346	−0.183, 0.713	0.266	−0.413, 0.670	−0.210	−0.400, −0.09	−0.028	−0.384, 0.332
**SAA2t**										
**n-3**	0.117	−0.219, 0.424	0.200	−0.209, 0.676	0.175	−0.157, 0.518	0.212	−0.311, 0.757	0.341	−0.097, 0.607
**n-6**	0.025	−0.437, 0.412	0.335	−0.131, 0.69	0.275	−0.374, 0.682	−0.148	−0.400, 0.216	−0.009	−0.376, 0.357
**S100At**										
**n-3**	−0.120	−0.526, 0.308	−0.067	−0.46, 0.325	−0.098	−0.500, 0.268	−0.336	−0.615, 0.095	0.229	−0.140, 0.510
**n-6**	−0.066	−0.468, 0.374	0.079	−0.302, 0.447	0.065	−0.367, 0.556	−0.279	−0.689, 0.425	−0.040	−0.457, 0.477
**S100A8**										
**n-3**	−0.162	−0.527, 0.208	−0.095	−0.504, 0.293	−0.132	−0.550, 0.225	−0.310	−0.597, 0.137	0.246	−0.165, 0.542
**n-6**	−0.082	−0.481, 0.337	0.099	−0.304, 0.489	0.075	−0.380, 0.571	−0.320	−0.724, 0.414	−0.054	−0.495, 0.417
**S100A9**										
**n-3**	0.169	−0.226, 0.54	0.118	−0.179, 0.462	0.143	−0.149, 0.452	−0.312	−0.648, 0.163	0.137	−0.193, 0.408
**n-6**	0.002	−0.397, 0.426	0.077	−0.323, 0.453	0.089	−0.321, 0.544	−0.168	−0.549, 0.461	0.010	−0.408, 0.521
**Eotaxin**										
**n-3**	−0.083	−0.501, 0.281	0.071	−0.439, 0.501	−0.028	−0.510, 0.446	−0.036	−0.550, 0.541	0.078	−0.351, 0.550
**n-6**	**−0.537***	−0.804, −0.114	**−0.647***	−0.882, −0.339	**−0.712****	−0.895, −0.461	−0.062	−0.470, 0.481	**−0.657****	−0.834, −0.334
**IL-8**										
**n-3**	0.147	−0.193, 0.429	0.205	−0.274, 0.597	0.196	−0.269, 0.625	−0.237	−0.589, 0.236	0.036	−0.424, 0.509

**n-6**	−0.173	−0.634, 0.778	−0.090	−0.529, 0.146	−0.144	−0.552, 0.377	0.109	−0.330, 0.778	−0.265	−0.599, 0.472
**MCP-1**										
**n-3**	0.150	−0.25, 0.586	0.082	−0.271, 0.531	0.131	−0.263, 0.603	0.010	−0.599, 0.496	−0.259	−0.568, 0.172
**n-6**	−0.458	−0.808, 0.153	**−0.591***	−0.88, −0.142	**−0.625****	−0.885, −0.203	−0.018	−0.365, 0.360	**−0.588***	−0.854, −0.116
**MIP-1α**										
**n-3**	0.139	−0.293, 0.537	−0.074	−0.422, 0.309	0.034	−0.402, 0.447	−0.178	−0.633, 0.324	−0.248	−0.616, 0.268
**n-6**	−0.300	−0.62, 0.197	−0.025	−0.426, 0.321	−0.136	−0.478, 0.244	0.108	−0.298, 0.397	−0.319	−0.578, 0.128
**MIP-1β**										
**n-3**	0.074	−0.484, 0.591	−0.116	−0.500, 0.398	−0.053	−0.543, 0.548	0.175	−0.432, 0.673	0.295	−0.202, 0.654
**n-6**	−0.227	−0.611, 0.175	−0.036	−0.686, 0.427	−0.106	−0.654, 0.353	0.214	−0.108, 0.530	−0.248	−0.574, 0.046
**RANTES**										
**n-3**	0.086	−0.301, 0.503	0.123	−0.607, 0.692	0.097	−0.460, 0.624	**0.593****	0.243, 0.825	−0.002	−0.413, 0.343
**n-6**	−0.240	−0.642, 0.299	0.133	−0.429, 0.65	0.001	−0.530, 0.565	0.016	−0.360, 0.308	−0.028	−0.543, 0.547
**TNF**										
**n-3**	−0.057	−0.586, 0.58	−0.283	−0.687, 0.364	−0.244	−0.738, 0.501	0.139	−0.513, 0.773	**0.616****	0.006, 0.834
**n-6**	−0.227	−0.58, 0.184	−0.020	−0.327, 0.309	−0.109	−0.441, 0.254	−0.066	−0.784, 0.585	−0.223	−0.529, 0.079
**IL-1RA**[3]										
**n-3**	0.149	−0.217, 0.62	−0.202	−0.677, 0.590	−0.038	−0.560, 0.641	−0.138	−0.647, 0.511	0.236	−0.516, 0.658
**n-6**	−0.420	−0.773, 0.145	−0.462	−0.691, −0.198	**−0.511***	−0.744, −0.131	−0.186	−0.622, 0.384	**−0.539***	−0.803, −0.135
**Adiponectin**										
**n-3**	0.056	−0.251, 0.329	0.258	−0.206, 0.621	0.196	−0.200, 0.524	**0.470***	0.101, 0.727	−0.142	−0.571, 0.293
**n-6**	−0.112	−0.632, 0.424	−0.137	−0.554, 0.277	−0.121	−0.525, 0.241	−0.376	−0.614, −0.157	−0.106	−0.522, 0.28
**Apelin**										
**n-3**	0.051	−0.349, 0.369	0.007	−0.403, 0.233	0.013	−0.431, 0.281	0.160	−0.084, 0.546	0.220	−0.335, 0.608
**n-6**	−0.258	−0.674, 0.559	0.179	−0.427, 0.619	−0.014	−0.529, 0.569	0.213	−0.149, 0.686	−0.194	−0.608, 0.496

[1] Values are Pearson's rs from bivariate correlation analyses using log_2 fold change,
[2] 95% BCa CI obtained from bootstrapping procedure using 1000 replicates.
[3] One outlier was removed from IL-1RA (n-3).
* Correlation is significant at 0.05**,** ** Correlation is significant at 0.01.
Abbreviations**:** BCa Cl, bias-corrected and accelerated confidence interval; DHA, docosahexaenoic acid; EPA, eicosapentaenoic acid; LA, linoleic acid; ARA, arachidonic acid; RBCMs, red blood cell membranes.

Supplemental Table 16. Genes regulated in the same direction in adipose tissue after 7 wk supplementation with n-3 or n-6 (q-value > 0.1, FC ≥ 0.1 or ≤ −0.1)

Upregulated genes	Downregulated genes
SAA1	CD52
SAA2	MMP9
LOC55908	COL8A1
CETP	FOSB
DGAT2	CCL2
DEFB32	SPP1
LOC645313	IL1RN
TF	TM4SF19
THBS4	ITGB2
CDKN2C	DHRS9
RERGL	LCP1
SAA4	PLA2G7
CRHBP	C1orf162
HP	IFI30
LRRN3	TPR
TMEM170B	ACP5
ACSL1	MATK
PPP2R1B	HCST
CD36	ALCAM
HEY1	PLEK
PTPLB	MMP7
LPL	FBP1
SLC2A5	SPOCD1
APOLD1	GSN
H19	CRIP1
IGF1	ZBTB16
C2orf40	ANGPTL4
RSPO3	ATF3
GPAM	AADACL1
SCD	CCL3L3
ELOVL5	GLIPR1
ANGPT1	MS4A6E
AGTR1	
CDO1	
CXCL12	
KLB	
FGFRL1	
RBP7	
LOC401052	
LOC643031	
VEGFC	
LTF	
EMCN	

Supplemental Table 17. Top 50 up- and down-regulated genes in adipose tissue after 7 wk supplementation with n-3 or n-6

Probe_ID	Gene name	Fold change	Pfp	P-value
**Omega-3**				
**Up-regulated**				
LOC100008589_ILMN_3251587	LOC100008589	1.382	2.16E-10	9.14E-15
ELOVL6_ILMN_2111187	ELOVL6	1.364	1E-08	2.12E-12
SAA1_ILMN_1701017	SAA1	1.350	3.22E-11	6.8E-16
SAA1_ILMN_2304512	SAA1	1.313	4.66E-09	5.9E-13
LOC55908_ILMN_1764714	LOC55908	1.305	5.45E-09	5.76E-13
DGAT2_ILMN_1681520	DGAT2	1.298	1.03E-08	1.74E-12
CES1_ILMN_1675386	CES1	1.291	6.01E-09	5.08E-13
SAA2_ILMN_1728262	SAA2	1.288	2.02E-10	1.28E-14
KANK4_ILMN_3241554	KANK4	1.272	6.73E-08	2.7E-11
FCN3_ILMN_1780060	FCN3	1.264	1.88E-08	5.95E-12
ILMN_1847494	N/A	1.244	1.04E-06	8.39E-10
SAA1_ILMN_1808732	SAA1	1.243	9.46E-09	2.6E-12
DEFB32_ILMN_1683887	DEFB32	1.243	1.13E-08	1.67E-12
ELOVL6_ILMN_1700546	ELOVL6	1.243	1.57E-05	2.22E-08
CETP_ILMN_2098013	CETP	1.232	9.11E-09	2.12E-12
CSN1S1_ILMN_1653006	CSN1S1	1.228	1.06E-08	2.01E-12
TF_ILMN_1768425	TF	1.221	5.47E-08	1.97E-11
CDKN2C_ILMN_1656415	CDKN2C	1.217	6.12E-07	4.52E-10
CES4_ILMN_2150095	CES4	1.215	6.68E-08	2.54E-11
PGM3_ILMN_1693620	PGM3	1.209	2.76E-05	4.25E-08
HEY1_ILMN_1788203	HEY1	1.208	1.42E-05	1.99E-08
LOC645313_ILMN_3277905	LOC645313	1.207	1.89E-08	6.38E-12
LRRN3_ILMN_1773650	LRRN3	1.203	5.15E-06	5.88E-09
ACSL1_ILMN_1684585	ACSL1	1.202	7.26E-06	9.05E-09
STK39_ILMN_1791328	STK39	1.200	6.79E-05	1.45E-07
NNAT_ILMN_1707734	NNAT	1.198	1.84E-06	1.59E-09
LPL_ILMN_1786444	LPL	1.197	2.62E-05	3.87E-08
CSN1S1_ILMN_2317364	CSN1S1	1.192	9.7E-08	4.92E-11
APLNR_ILMN_1700183	APLNR	1.191	2.2E-05	3.21E-08
CD36_ILMN_1665132	CD36	1.188	8.81E-06	1.16E-08
MRAP_ILMN_1705676	MRAP	1.187	4.07E-05	7.84E-08
ANKRD38_ILMN_1776936	ANKRD38	1.187	3.06E-06	2.84E-09
TSPAN3_ILMN_1655469	TSPAN3	1.185	0.000111	2.91E-07
PTPLB_ILMN_2170353	PTPLB	1.184	2.59E-05	3.89E-08
LPIN1_ILMN_1671554	LPIN1	1.181	6.5E-05	1.33E-07
TUBB2A_ILMN_2038775	TUBB2A	1.177	3.49E-06	3.47E-09
PTPLB_ILMN_1690806	PTPLB	1.177	2.06E-05	2.96E-08
PODXL_ILMN_2413158	PODXL	1.177	8.95E-05	2.16E-07
LRRN3_ILMN_2048591	LRRN3	1.175	5.35E-05	1.06E-07
MME_ILMN_1678170	MME	1.174	8.93E-05	2.11E-07
ANGPT1_ILMN_2086890	ANGPT1	1.173	0.000135	3.78E-07
STOX1_ILMN_1664014	STOX1	1.173	8.94E-05	2.13E-07
SERPINI1_ILMN_1814333	SERPINI1	1.171	8.26E-05	1.9E-07
LOC100133565_ILMN_3239610	LOC100133565	1.171	8.94E-06	1.15E-08
GLYAT_ILMN_1668510	GLYAT	1.171	6.82E-05	1.49E-07
PRDX2_ILMN_1767766	PRDX2	1.168	3.99E-05	7.6E-08
MRAP_ILMN_1659769	MRAP	1.167	9.27E-05	2.33E-07
DHRS11_ILMN_1756701	DHRS11	1.166	4.05E-05	7.53E-08
IDH1_ILMN_1696432	IDH1	1.165	7.24E-05	1.62E-07

**Down-regulated**				
LOC389342_ILMN_3280565	LOC389342	0.741	2.41E-10	1.53E-14
CD52_ILMN_2208903	CD52	0.750	2.19E-09	1.85E-13
HBG1_ILMN_1796678	HBG1	0.755	6.9E-11	1.46E-15
HBG2_ILMN_2084825	HBG2	0.765	5.61E-11	2.37E-15
AQP9_ILMN_1715068	AQP9	0.773	3.28E-08	5.55E-12
CXCL9_ILMN_1745356	CXCL9	0.774	2.4E-07	7.6E-11
IL1RN_ILMN_1774874	IL1RN	0.779	3.96E-07	1.67E-10
LTB_ILMN_2376205	LTB	0.812	1.1E-05	1.2E-08
FOSB_ILMN_1751607	FOSB	0.821	2.89E-07	9.77E-11
CXCL10_ILMN_1791759	CXCL10	0.821	2.94E-07	1.06E-10
COL8A1_ILMN_1685433	COL8A1	0.823	6.95E-08	1.76E-11
C6_ILMN_1688242	C6	0.825	3.38E-07	1.36E-10
FOS_ILMN_1669523	FOS	0.826	4.54E-06	4.22E-09
CYFIP2_ILMN_1677200	CYFIP2	0.829	0.002306	1.17E-05
CCL2_ILMN_1720048	CCL2	0.829	3.01E-07	1.15E-10
TNC_ILMN_1719759	TNC	0.829	0.000922	3.06E-06
FGFBP2_ILMN_1761945	FGFBP2	0.835	7.57E-07	3.84E-10
ITGB2_ILMN_1654396	ITGB2	0.835	1.3E-06	7.4E-10
ENPP2_ILMN_1780799	ENPP2	0.838	4.98E-05	8.31E-08
FLNC_ILMN_1715748	FLNC	0.840	0.000954	3.24E-06
S100A8_ILMN_1729801	S100A8	0.841	1.3E-08	1.65E-12
C1orf162_ILMN_1754894	C1orf162	0.842	2.27E-06	1.58E-09
ENPP2_ILMN_2373791	ENPP2	0.842	5.12E-05	8.77E-08
LCP1_ILMN_1662932	LCP1	0.843	1.69E-06	1.03E-09
HBA1_ILMN_3240144	HBA1	0.843	4E-06	3.38E-09
CSF3R_ILMN_2371280	CSF3R	0.844	1.91E-06	1.21E-09
LILRB3_ILMN_1784884	LILRB3	0.844	9.72E-06	9.86E-09
PRKCB1_ILMN_1806908	PRKCB1	0.845	1.87E-05	2.56E-08
ITGB2_ILMN_2175912	ITGB2	0.846	1.96E-06	1.32E-09
TOB1_ILMN_1672004	TOB1	0.846	0.000167	3.35E-07
CCL5_ILMN_2098126	CCL5	0.847	1.09E-05	1.18E-08
FGR_ILMN_1795158	FGR	0.847	2.68E-05	3.85E-08
IFI30_ILMN_1807277	IFI30	0.850	2.85E-06	2.17E-09
FGR_ILMN_2368318	FGR	0.850	4.22E-06	3.65E-09
ACP5_ILMN_2078599	ACP5	0.850	1.17E-05	1.34E-08
PLEK_ILMN_1795762	PLEK	0.852	1.78E-05	2.3E-08
FBP1_ILMN_1728799	FBP1	0.854	0.000435	1.11E-06
MATK_ILMN_2319000	MATK	0.855	1.26E-05	1.46E-08
DHRS9_ILMN_1733998	DHRS9	0.858	1.51E-06	8.9E-10
SPOCD1_ILMN_1784948	SPOCD1	0.860	0.000445	1.15E-06
SLC7A7_ILMN_1810275	SLC7A7	0.861	7.76E-05	1.43E-07
CIDEA_ILMN_2390318	CIDEA	0.861	4.61E-06	4.19E-09
RAC2_ILMN_1709795	RAC2	0.862	1.84E-05	2.44E-08
TYROBP_ILMN_1778977	TYROBP	0.862	1.96E-05	2.73E-08
LAPTM5_ILMN_1772359	LAPTM5	0.864	4.51E-05	6.96E-08
VCAN_ILMN_1687301	VCAN	0.865	7.6E-05	1.37E-07
HCST_ILMN_1699931	HCST	0.866	1.47E-05	1.74E-08
C7_ILMN_1687848	C7	0.866	4.9E-05	8.08E-08
MMP7_ILMN_1685403	MMP7	0.866	0.000375	9.04E-07
**Omega-6**				
**Up-regulated**				
MYH11_ILMN_1660086	MYH11	1.637	1.54E-15	3.25E-20
ACTG2_ILMN_1795325	ACTG2	1.577	2.58E-11	1.09E-15

LOC55908_ILMN_1764714	LOC55908	1.382	9.26E-10	1.17E-13
S100A9_ILMN_1750974	S100A9	1.327	3.45E-10	2.91E-14
RERGL_ILMN_3243185	RERGL	1.319	8.75E-10	9.25E-14
TF_ILMN_1768425	TF	1.309	3.81E-09	5.64E-13
S100A8_ILMN_1729801	S100A8	1.298	7.32E-11	4.64E-15
CRHBP_ILMN_1761312	CRHBP	1.280	3.41E-07	1.52E-10
COL1A1_ILMN_1701308	COL1A1	1.271	4.34E-08	1.01E-11
FCN1_ILMN_1668063	FCN1	1.253	1.38E-06	8.75E-10
PPBP_ILMN_1767281	PPBP	1.250	3.56E-08	7.51E-12
CASQ2_ILMN_1722329	CASQ2	1.250	1.75E-07	5.91E-11
PPP1R1B_ILMN_2304495	PPP1R1B	1.240	7.38E-08	2.03E-11
ACTA2_ILMN_1671703	ACTA2	1.226	4.66E-06	3.94E-09
HP_ILMN_1812433	HP	1.225	6.72E-07	3.69E-10
CCL5_ILMN_1773352	CCL5	1.220	8.51E-06	8.45E-09
DGAT2_ILMN_1681520	DGAT2	1.211	4.98E-06	4.31E-09
FPR1_ILMN_2092118	FPR1	1.208	3.07E-05	4.28E-08
DDIT4_ILMN_1661599	DDIT4	1.208	1.59E-05	2.09E-08
RGS2_ILMN_2197365	RGS2	1.206	1.1E-06	6.51E-10
CALD1_ILMN_1717990	CALD1	1.203	2.04E-05	2.76E-08
LOC645313_ILMN_3277905	LOC645313	1.196	1.71E-07	6.52E-11
VNN2_ILMN_1678939	VNN2	1.191	1.46E-05	1.66E-08
PCK1_ILMN_1731948	PCK1	1.189	2.51E-05	3.44E-08
HBA1_ILMN_3240144	HBA1	1.189	1.34E-05	1.44E-08
APOLD1_ILMN_1723522	APOLD1	1.188	1.48E-05	1.78E-08
VCAM1_ILMN_2307903	VCAM1	1.187	3.42E-07	1.59E-10
THBS4_ILMN_1736078	THBS4	1.184	4.47E-08	1.13E-11
OLFML2B_ILMN_1765557	OLFML2B	1.184	0.0001	1.82E-07
SORBS2_ILMN_2407879	SORBS2	1.180	0.00036	9.82E-07
SAA1_ILMN_2304512	SAA1	1.178	1.04E-06	5.93E-10
COPG2IT1_ILMN_3242623	COPG2IT1	1.178	0.0001	1.84E-07
MYLK_ILMN_1691476	MYLK	1.175	0.00057	1.7E-06
EMCN_ILMN_1685917	EMCN	1.174	0.000551	1.63E-06
H19_ILMN_2148527	H19	1.173	9.71E-05	1.74E-07
PTPLB_ILMN_1690806	PTPLB	1.172	7.23E-05	1.13E-07
HBA2_ILMN_2127842	HBA2	1.170	0.001681	7.46E-06
LOC653879_ILMN_1713182	LOC653879	1.168	0.000364	1E-06
GPAM_ILMN_1758213	GPAM	1.168	8.77E-05	1.52E-07
CETP_ILMN_2098013	CETP	1.167	1.57E-05	1.99E-08
TMEM170B_ILMN_3236428	TMEM170B	1.166	1.82E-06	1.19E-09
SAA1_ILMN_1701017	SAA1	1.166	5.53E-09	9.36E-13
RBP7_ILMN_1716465	RBP7	1.165	0.001323	5.31E-06
ENPEP_ILMN_1675325	ENPEP	1.164	0.000662	2.06E-06
KLB_ILMN_1731688	KLB	1.164	7.92E-05	1.29E-07
PI3_ILMN_1693192	PI3	1.161	0.005281	3.73E-05
ALPL_ILMN_1701603	ALPL	1.160	8.68E-05	1.45E-07
SAA2_ILMN_1728262	SAA2	1.160	5.55E-07	2.81E-10
SAA4_ILMN_1668055	SAA4	1.158	8.22E-06	7.99E-09
**Down-regulated**				
TM4SF19_ILMN_2413644	TM4SF19	0.651	2.82E-12	1.19E-16
MMP9_ILMN_1796316	MMP9	0.675	1.13E-12	2.39E-17
SPP1_ILMN_1651354	SPP1	0.716	5.67E-12	3.6E-16
SPP1_ILMN_2374449	SPP1	0.729	1.93E-11	1.63E-15
MATK_ILMN_2319000	MATK	0.730	3.64E-10	5.38E-14
TM4SF19_ILMN_1808325	TM4SF19	0.732	5.05E-09	9.61E-13

IL1RN_ILMN_1774874	IL1RN	0.733	2.81E-10	3.57E-14
FCGBP_ILMN_2302757	FCGBP	0.766	2.3E-11	2.43E-15
PLA2G7_ILMN_1701195	PLA2G7	0.791	7.59E-09	1.77E-12
LOC392437_ILMN_3288717	LOC392437	0.791	2.25E-07	8.08E-11
SPOCD1_ILMN_1784948	SPOCD1	0.792	1.44E-06	9.75E-10
THBS1_ILMN_1686116	THBS1	0.794	1.41E-07	4.78E-11
RARRES1_ILMN_1800091	RARRES1	0.799	7.38E-07	3.59E-10
IL1RN_ILMN_1689734	IL1RN	0.806	7.49E-07	3.32E-10
ACP5_ILMN_2078599	ACP5	0.808	1.29E-07	4.08E-11
HAVCR2_ILMN_1693826	HAVCR2	0.814	1.49E-06	9.42E-10
TFRC_ILMN_1674243	TFRC	0.815	6.9E-07	2.92E-10
ILMN_1867119	N/A	0.815	3.32E-06	2.95E-09
FBP1_ILMN_1728799	FBP1	0.816	6.96E-06	7.94E-09
AADACL1_ILMN_1676336	AADACL1	0.817	2.52E-06	2.02E-09
CHI3L1_ILMN_3307868	CHI3L1	0.827	1.5E-05	1.96E-08
C13orf33_ILMN_1696048	C13orf33	0.829	8.54E-06	9.93E-09
ALCAM_ILMN_1670870	ALCAM	0.831	1.23E-06	6.77E-10
FOSB_ILMN_1751607	FOSB	0.834	7.2E-07	3.65E-10
SLC31A2_ILMN_1758938	SLC31A2	0.840	1.18E-05	1.49E-08
FKBP5_ILMN_1778444	FKBP5	0.843	1.86E-06	1.38E-09
ALDH1A3_ILMN_2139970	ALDH1A3	0.845	2.68E-06	2.27E-09
EMILIN2_ILMN_1697268	EMILIN2	0.845	1.15E-05	1.38E-08
RARRES1_ILMN_1743620	RARRES1	0.846	8E-05	1.54E-07
MS4A6E_ILMN_1759933	MS4A6E	0.847	9.22E-05	1.85E-07
PLEK_ILMN_1795762	PLEK	0.847	3.09E-06	2.68E-09
IFI30_ILMN_1807277	IFI30	0.849	1.28E-06	7.27E-10
DHRS9_ILMN_1733998	DHRS9	0.849	1.92E-06	1.34E-09
CD52_ILMN_2208903	CD52	0.851	2.16E-07	8.22E-11
GPC4_ILMN_1789502	GPC4	0.852	7.58E-05	1.39E-07
TPR_ILMN_1780349	TPR	0.853	7.28E-07	3.39E-10
SLC15A3_ILMN_2085862	SLC15A3	0.853	2.36E-05	3.24E-08
NRIP3_ILMN_1759563	NRIP3	0.853	0.000253	6.95E-07
LIPA_ILMN_1718063	LIPA	0.854	6.04E-06	5.87E-09
ATF3_ILMN_2374865	ATF3	0.855	6.47E-05	1.15E-07
CDC20_ILMN_1663390	CDC20	0.855	0.000126	2.75E-07
TUBA1C_ILMN_3251341	TUBA1C	0.858	0.000377	1.18E-06
CXCL16_ILMN_1728478	CXCL16	0.861	1.59E-05	2.12E-08
TM7SF4_ILMN_1793730	TM7SF4	0.861	0.001486	7.85E-06
ITGB2_ILMN_1654396	ITGB2	0.861	3.97E-06	3.61E-09
MMP7_ILMN_2192072	MMP7	0.862	5.84E-05	1E-07
ZBTB16_ILMN_2305407	ZBTB16	0.863	0.00015	3.45E-07
LCP1_ILMN_1662932	LCP1	0.865	1.88E-06	1.35E-09
LOXL4_ILMN_2179083	LOXL4	0.866	0.001427	7.36E-06

Supplemental Table 18. Top up- and down-regulated "hallmark gene sets" from Gene Set Enrichment Analysis (GSEA) in adipose tissue after 7 wk supplementation with n-3 or n-6

**Omega-3**				
**Pathway**	**Size**	**NES**	**AdjP**	**Leading Edge Genes**
ADIPOGENESIS	185	2.87	1.67E-09	CHCHD10, SLC25A1, MRAP, ITSN1, DGAT1, ADIPOR2, PFKFB3, ITIH5, PHYH, REEP6, CD151, ARL4A, COL4A1, LPL, PPARG, CMBL, TST, NKIRAS1, SLC5A6, QDPR, SAMM50, COL15A1, PPM1B, LPCAT3, NDUFAB1, STOM, AIFM1, SULT1A1, YWHAG, PEMT, CD36, ALDOA, GPHN, SORBS1, AK2, ANGPT1, SUCLG1, ME1, IMMT, RTN3, COQ3, PDCD4, GPAM, ELOVL6, PRDX3, DLD, IDH3A, ACLY, CYC1, CDKN2C, RNF11, CS, FAH, ECHS1, CPT2, LEP, MYLK, PGM1, UCP2, MGLL, PTGER3, ACADM, ETFB, ATP1B3, DLAT, HIBCH, MCCC1, BCL6, ACOX1, IDH1, SCARB1, ACADL, SLC25A10, UBQLN1, PTCD3, COQ9, GPD2, SDHC
FATTY ACID METABOLISM	154	2.68	1.67E-09	SUCLA2, LDHA, ADIPOR2, BPHL, PDHA1, AQP7, REEP6, GRHPR, ACO2, BLVRA, XIST, PCBD1, ACADVL, ALAD, MDH1, CD36, CA2, ALDOA, ELOVL5, ALDH3A2, ACOT2, HSD17B4, SUCLG1, BCKDHB, ME1, GPD1, SLC22A5, PDHB, HADH, OSTC, DLD, SERINC1, HSP90AA1, UROS, ECHS1, CPT2, DHCR24, CCDC58, MGLL, CRYZ, ACADM, ETFDH, ACSL1, SDHD, HIBCH, ERP29, EHHADH, ACOX1, IDH1, ACADL, FASN, HSD17B7, ALDH9A1, GLUL, G0S2, EPHX1, FH, GCDH, NSDHL, CRAT, GPD2, PTS, SDHC, ENO3, NBN, RDH11, GSTZ1, RAP1GDS1
OXIDATIVE PHOSPHORYLATION	179	2.52	1.67E-09	NDUFA6, SUCLA2, LDHA, PDHA1, PHYH, NDUFV2, MTRR, CYCS, ACO2, NNT, NDUFS4, ACADVL, MRPL15, PDHX, NDUFAB1, COX10, AIFM1, CYB5A, MDH1, LDHB, SLC25A20, TIMM10, UQCRFS1, ALDH6A1, SUCLG1, MRPS15, IMMT, MRPL35, PDHB, VDAC1, COX5A, PRDX3, DLD, UQCRH, IDH3A, CYC1, OAT, CS, ECHS1, NDUFC1, ACADM, NDUFB2, ETFDH, ETFB, DLAT, SDHD, MTX2, NDUFB5, OPA1, IDH1, FH, NDUFS1, CYB5R3, RHOT1, ATP6V0E1, MRPS22, SLC25A3, NDUFB3, SDHC, SLC25A5, NDUFS2, TIMM13
MTORC1 SIGNALING	190	2.09	6.10E-08	ACACA, PSMC4, VLDLR, LDHA, XBP1, STC1, PSAT1, QDPR, GSR, PPA1, DDIT4, CCT6A, TCEA1, HSPA5, PSMA3, ALDOA, ELOVL5, GLRX, HK2, FADS2, FADS1, SCD, ME1, IMMT, ELOVL6, ACLY, TUBG1, PSME3, DHCR24, PGM1, PSMD14, PLOD2, HSP90B1, TXNRD1, PHGDH, ATP2A2, HMBS, HSPD1, HSPE1, IDH1, IGFBP5, CACYBP, SLC6A6, EGLN3, PSPH, DHFR, ETF1, PSMA4, CYB5B
GLYCOLYSIS	194	2.08	4.62E-08	PSMC4, VLDLR, LDHA, IRS2, KDELR3, STC1, PFKFB1, PPP2CB, IGFBP3, DDIT4, PRPS1, PYGL, HSPA5, CYB5A, MDH1, UGP2, GFPT1, ALDOA, GLRX, PGAM1, HK2, ALDH7A1, ME1, GLCE, NT5E, DLD, PC, DPYSL4, CITED2, B4GALT7, AGL, DCN, PLOD2, HAX1, IDH1, RPE, SLC25A10, ABCB6, ALDH9A1, CHST12, B4GALT1, SDC1, ENO1, EGLN3, PGK1, P4HA1, ECD, EGFR, TPI1, SRD5A3, EXT2, GMPPB, NSDHL, G6PD, SLC25A13, P4HA2, NOL3, LHPP, XYLT2, HS2ST1, SAP30, SLC35A3, COPB2, SDHC, BPNT1, SOD1, LDHC, GALK2, GYS2, CLDN9, CENPA, SDC3, MPI, B3GAT1, CLN6, ALG1
PEROXISOME	101	2.00	4.55E-05	HRAS, ITGB1BP1, CTBP1, TSPO, CAT, MVP, SEMA3C, ELOVL5, PABPC1, SIAH1, HSD17B4, FADS1, ISOC1, DHCR24, PEX11A, IDH2, CNBP, ACSL1, CADM1, EHHADH, ACOX1, IDH1, FDPS, ALDH9A1, ECH1, ABCB1, CRAT, SCP2, ERCC1, ABCC5, SOD1, ALB, ESR2, RDH11, ABCD2, CRABP1, DIO1, ABCB9, RXRG, PEX13, CLN6, IDE, SCGB1A1
UV RESPONSE DN	134	1.93	4.14E-05	TJP1, VLDLR, DYRK1A, CAP2, DLC1, ICA1, PPARG, NR1D2, APBB2, SNAI2, PMP22, EFEMP1, ANXA2, BCKDHB, ADD3, GRK5, GJA1, CAV1, MGMT, DBP, MGLL, CITED2, RND3, ATP2C1, F3, COL11A1, TFPI, COL3A1, COL5A2, IGFBP5, IRS1, ATP2B4, ATRX, ANXA4, ZMIZ1, NOTCH2, PRDM2, PDLIM5, ACVR2A, DLG1, LTBP1, NEK7, TGFBR2, IGF1R, CDON, PRKAR2B
MYC TARGETS V1	185	1.92	9.93E-06	MRPS18B, PSMC4, CANX, EEF1B2, RANBP1, LDHA, EIF1AX, AIMP2, ACP1, TFDP1, CCT5, EIF4G2, PSMA2, C1QBP, RAD23B,

				PSMA6, NDUFAB1, CCT7, PABPC1, SYNCRIP, VDAC1, COX5A, PRDX3, CYC1, BUB3, IFRD1, G3BP1, CNBP, PSMD14, TCP1, NME1, HSPD1, HSPE1, RPLP0, NCBP2, RAN, SSBP1, HNRNPR, PCNA, UBE2L3, PRDX4, UBE2E1, PGK1, GLO1, UBA2, GNL3, VBP1, ETF1, PSMA4, SF3B3, RUVBL2, NHP2, SLC25A3, RPL6, KPNA2, PSMD1, EIF3J, PSMA7, XPOT, COPS5, SNRPA1, RRP9
CHOLESTEROL HOMEOSTASIS	72	1.85	1.08E-03	ACSS2, LPL, PPARG, CPEB2, CLU, ERRFI1, FBXO6, CTNNB1, ANTXR2, GNAI1, ALDOC, FADS2, SCD, MAL2, ATF5, GSTM2, FDPS, PCYT2, FASN, HSD17B7, CBS, NSDHL, MVD, ABCA2
ANDROGEN RESPONSE	96	1.85	5.56E-04	TSC22D1, MYL12A, GSR, PMEPA1, ELOVL5, ELL2, TNFAIP8, FADS1, SCD, PGM3, AZGP1, DBI, CCND1, AKT1, DHCR24, AKAP12, STK39, ITGAV, UAP1, RPS6KA3, B4GALT1, NCOA4, ZMIZ1, STEAP4, CDK6, SRP19, SPCS3, ACSL3, ANKH, ZBTB10, PDLIM5
MYOGENESIS	197	1.83	4.55E-05	MYOM2, MYH11, SGCG, APP, TNNT3, DMD, MYOM1, ITGB1, LPIN1, MYO1C, CLU, DTNA, SOD3, TEAD4, COL15A1, IGFBP3, NAV2, VIPR1, CKMT2, AK1, OCEL1, CD36, APLNR, SORBS1, SCD, NOTCH1, PC, IFRD1, NQO1, SPARC, GABARAPL2, MYLK, ITGB5, CDH13, MRAS, AGL, ACSL1, IGF1, CASQ2, COL3A1, HSPB8, TPM3, APOD, FDPS, RB1, LSP1, SORBS3, PKIA, SPTAN1, CRAT, ABLIM1, ADAM12, BIN1, FGF2, SGCD, HRC, SH3BGR, ENO3, MYL6B, PDE4DIP, TSC2, MYL1, KCNH1, ATP2A1, ACHE, PSEN2, MB, MEF2A, NCAM1, AKT2, CFD, NOS1
PROTEIN SECRETION	95	1.80	0.001	RAB9A, ICA1, RER1, SNAP23, RAB2A, LAMP2, DNM1L, TMED10, VAMP7, CAV2, AP1G1, CLCN3, CTSC, CLTC, SEC22B, VAMP4, SCAMP3, RPS6KA3, CLTA, GOLGA4, ABCA1, EGFR, SCAMP1, SEC31A, ADAM10, COPE, BNIP3, AP2S1, GOSR2, COPB2, SOD1, COPB1, BET1, GALC, ARFIP1, TOM1L1, KRT18, STAM, RAB14
XENOBIOTIC METABOLISM	196	1.69	0.001	CES1, SLC46A3, AKR1C2, AKR1C3, BPHL, ACP1, ID2, HACL1, ACO2, GSR, PGD, PTGR1, PGRMC1, CYB5A, CNDP2, PEMT, ACP2, CD36, CA2, ELOVL5, RBP4, CDO1, SAR1B, ENPEP, CSAD, JUP, PC, FAH, NQO1, IRF8, ETFDH, ABCC3, IGF1, ATP2A2, ACOX1, IDH1, ALDH9A1, GART, COMT, NDRG2, SLC6A6, LONP1, LEAP2, SLC35B1, PTS, MCCC2, NFS1, ABCD2, PDLIM5, LCAT, BCAR1, CYP4F2, ASL, REG1A, GAD1
BILE ACID METABOLISM	111	1.68	0.002	PHYH, HACL1, AR, BBOX1, HSD17B4, FADS2, FADS1, ISOC1, CYP46A1, DHCR24, PEX11A, IDH2, GCLM, ACSL1, ABCA9, IDH1, PXMP2, PECR, PEX19, ALDH9A1, AMACR, SCP2, PEX7, AKR1D1, SOD1, ABCD2, ABCA2, PAOX, DIO1, RXRG, SLCO1A2, ALDH8A1, PEX13, PEX26, SOAT2, SLC23A1
SPERMATOGENESIS	128	1.68	0.001	YBX2, ARL4A, LPIN1, DMC1, POMC, SLC2A5, PRKAR2A, SLC12A2, ELOVL3, SHE, AGFG1, TALDO1, MLLT10, PIAS2, GSG1, CCT6B, NEFH, LDHC, CFTR, MAP7, EZH2, SYCP1, ACRV1, THEG, PHF7, PDHA2, ZC3H14, NPHP1, STAM2, ACE, CLGN, TNNI3, CST8, PAPOLB, DBF4, DDX25, IDE, TKTL1, GRM8, GAD1, MLF1, ZPBP, CRISP2, KIF2C, HOXB1, NOS1
HEDGEHOG SIGNALING	35	1.61	0.016	VLDLR, TLE1, AMOT, HEY1, NRCAM, CDK6, PML, GLI1, ACHE
NOTCH SIGNALING	32	1.61	0.028	LFNG, NOTCH3, NOTCH1, ST3GAL6, CCND1, DTX1, FBXW11, SAP30, NOTCH2, ARRB1, MAML2, PRKCA, PSEN2, WNT2
REACTIVE OXYGEN SPECIES PATHWAY	44	1.61	0.028	NDUFA6, MSRA, GSR, PRDX2, MGST1, GLRX, NQO1, GCLM, TXNRD1, GLRX2, LSP1, PRDX4, NDUFS2, FTL
HYPOXIA	188	1.56	0.003	SLC25A1, VLDLR, LDHA, PFKFB3, TPST2, IRS2, GRHPR, KDELR3, STC1, BNIP3L, DTNA, ERRFI1, SLC2A5, IGFBP3, DDIT4, PCK1, HSPA5, BCL2, UGP2, ALDOA, ANXA2, GLRX, WSB1, BGN, ALDOC, HK2, LOX, CAV1, PGM1, CITED2, AKAP12, F3, DCN, BTG1, LXN, ETS1, SELENBP1, SCARB1, SLC6A6, ENO1, PGK1, P4HA1, ILVBL, P4HA2, SAP30, JMJD6, ENO3, LDHC, EDN2
G2M CHECKPOINT	180	1.51	0.008	DMD, KIF5B, TFDP1, SS18, RAD23B, MNAT1, SFPQ, RAD21, SLC12A2, SYNCRIP, AMD1, MARCKS, BUB3, CDKN2C, CCND1, POLQ, G3BP1, ATF5, HSPA8, HIRA, NUP98, GSPT1, ILF3, ATRX, HIF1A, MEIS1, ABL1, PML, E2F4, KATNA1, SAP30, SMC4, NOTCH2, KPNA2, NCL, SMC2, ARID4A, FOXN3, RPS6KA5, CDC6, EZH2, UCK2, LBR, RBL1, CENPA, CDC25A, INCENP, FBXO5, STIL, BIRC5, DTYMK

PI3K AKT MTOR SIGNALING	103	1.43	0.028	HRAS, ACACA, PLCB1, MAPKAP1, PRKAR2A, ITPR2, MAPK10, MAP2K3, RALB, AKT1, NCK1, VAV3, PPP2R1B, HSP90B1, CLTC, PTPN11, RPS6KA3, YWHAB, EGFR, MKNK1, GSK3B, PFN1, EIF4E, UBE2D3, IL4, TSC2, PITX2, UBE2N, IL2RG, SMAD2, GNA14, MAP3K7, FGF22
WNT BETA CATENIN SIGNALING	42	1.42	0.073	NOTCH4, CTNNB1, HEY1, GNAI1, NOTCH1, JAG2, SKP2, RBPJ, NCSTN, ADAM17, TCF7
UNFOLDED PROTEIN RESPONSE	106	1.41	0.028	TUBB2A, XBP1, KIF5B, KDELR3, FKBP14, PSAT1, DDIT4, SLC30A5, HSPA5, IFIT1, BAG3, BANF1, DNAJA4, SRPRB, HSP90B1, PDIA6, TSPYL2, SDAD1, SEC31A, TTC37, EXOSC2, DCTN1, IMP3, GOSR2, NHP2, SPCS3, SHC1, PARN, SSR1, LSM1, EIF4E, HSPA9, XPOT, CNOT4, CNOT6, RRP9
HEME METABOLISM	186	1.37	0.028	FBXO7, BNIP3L, C3, TNRC6B, BLVRA, NNT, PRDX2, EPB42, ALAD, TCEA1, LMO2, CAT, SDCBP, LAMP2, CA2, ELL2, MAP2K3, CAST, RNF19A, ALDH6A1, ERMAP, FOXO3, GLRX5, PC, UROS, GYPC, UCP2, CLCN3, PICALM, GCLM, HMBS, SELENBP1, TRAK2, TNS1, ABCB6, SYNJ1, RIOK3, NCOA4, EPOR, FECH, GAPVD1, FBXO9, P4HA2, ARHGEF12, PSMD9, YPEL5, ICAM4, KLF3, ADD2, EPB41, GYPE, TFDP2, IGSF3, DCUN1D1, CTSE, TRIM58, LPIN2, SPTA1, NEK7, TRIM10, SLC6A9, GATA1, BTRC
ANGIOGENESIS	36	1.31	0.113	APP, POSTN, LPL, STC1, JAG2, SLCO2A1, ITGAV, COL3A1, COL5A2, PRG2, PTK2
E2F TARGETS	183	1.27	0.058	TUBB, SHMT1, RANBP1, RBBP7, PNN, PRPS1, AK2, RAD21, SYNCRIP, CDKN2C, TUBG1, NME1, RAN, PCNA, PSIP1, PRDX4, PMS2, SMC6, PHF5A, IPO7, GSPT1, ILF3, TBRG4, PRKDC, PAICS, RFC2, STMN1, POLE4, RAD50, PAN2, SMC4, RAD51C, KPNA2, RFC3, NBN, BRCA1, EZH2, LBR, TRIP13, SSRP1, TCF19, CDC25A, CCNE1, NASP, RAD1, PPP1R8, BIRC5, ASF1A, KIF2C
DNA REPAIR	138	1.27	0.087	MRPL40, POLR2E, RALA, SDCBP, AK1, BCAP31, TAF1C, NME1, NCBP2, TAF9, PCNA, RAD51, RAE1, ARL6IP1, CSTF3, RFC2, BOLA2, RRM2B, DDB1, POLE4, NUDT9, SNAPC5, TAF6, SMAD5, TK2, SAC3D1, DCTN4, ERCC1, PDE4B, TAF13, GTF2H3, RFC3, POLA1, ERCC8, EDF1, APRT, SUPT4H1, SSRP1, ERCC4, PRIM1, BOLA2B
ESTROGEN RESPONSE EARLY	188	1.27	0.065	NRIP1, CALB2, TUBB2B, XBP1, DYNLT3, DLC1, CXCL12, AR, SLC39A6, IL6ST, NAV2, MAST4, BCL2, ELOVL5, RHOBTB3, AMFR, SLC22A5, ADD3, LRIG1, RARA, GJA1, CCND1, PEX11A, PODXL, WWC1, TSKU, CHPT1, HSPB8, SCARB1, FASN, MLPH, MAPT, B4GALT1, RBBP8, AFF1, ABLIM1, RPS6KA2, SLC19A2, SYNGR1, ABAT, GFRA1, TMPRSS3, CBFA2T3, KRT18, LAD1, MSMB, IGF1R, RAPGEFL1, HR, ADCY1, SLC1A1, TFF1, SEC14L2, CELSR2
TGF BETA SIGNALING	53	1.26	0.147	TJP1, FKBP1A, ID2, PMEPA1, CTNNB1, SPTBN1, PPM1A, SMAD6, HIPK2, LEFTY2, TGIF1, XIAP, RHOA, BMPR2, UBE2D3, TRIM33, SMAD1, SMURF2
ESTROGEN RESPONSE LATE	194	1.24	0.076	NRIP1, XBP1, TPSAB1, DYNLT3, TSPAN13, ID2, FGFR3, TST, SNX10, CXCL12, IL6ST, PTPN6, LTF, BCL2, MDK, CA2, ELOVL5, ALDH3A2, ASS1, TFPI2, AMFR, SLC22A5, ADD3, PDCD4, CAV1, CCND1, JAK1, IDH2, MEST, PTGER3, CPE, ETFB, CHPT1, HSPB8, SCARB1, ATP2B4, METTL3, COX6C, AFF1, RPS6KA2, DCXR, CDC6, TMPRSS3, DNAJC12, CACNA2D2, LSR, STIL, PRKAR2B, HR
APICAL SURFACE	43	1.21	0.177	APP, ADIPOR2, SLC22A12, DCBLD2, B4GALT1, ADAM10, RTN4RL1, CROCC, AKAP7, LYPD3, BRCA1, IL2RG
PANCREAS BETA CELLS	40	1.19	0.216	FOXO1, SRP14, LMO2, SRPRB, SCGN, SRP9, AKT3, FOXA2, DCX, PKLR, CHGA, STXBP1, ISL1, PAX6, PAX4, GCG, NEUROD1
KRAS SIGNALING DN	188	1.19	0.113	LGALS7B, OXT, P2RX6, MX1, YBX2, EDN1, LFNG, GAMT, FGFR3, SNN, SLC16A7, SPHK2, IFI44L, KRT1, FGGY, CPA2, CCDC106, MFSD6, LGALS7, ARHGDIG, CAMK1D, CDKAL1, TGM1, NRIP2, PLAG1, THNSL2, EDN2, KRT5, LYPD3, CD80, ASB7, CAPN9, AMBN, DLK2, SNCB, KLK8, TNNI3, KCNQ2, SMPX, CALCB, NR6A1, ITIH3, PNMT, BRDT, KLK7, SHOX2, SKIL, CLDN16, FGF22, FSHB, GPR19, SCGB1A1, CLPS, GPR3, KRT13, PCDHB1, CELSR2, NOS1
MITOTIC SPINDLE	194	1.17	0.133	ITSN1, NET1, NEDD9, MID1IP1, FSCN1, RAPGEF5, KIF5B, SOS1, VCL, SPTBN1, PKD2, KATNB1, MARCKS, EPB41L2, NCK1,

				CDC42EP4, CLIP1, ATG4B, MID1, KIF3B, CDC42EP1, TRIO, ROCK1, HOOK3, NIN, SPTAN1, ABL1, SAC3D1, KLC1, BIN1, KATNA1, TLK1, SMC4, NOTCH2, ARHGEF12, ALS2, CTTN, EPB41, PDLIM5, DLG1, BCAR1, BCR, ARHGEF7, INCENP, FBXO5, RHOF, KPTN, BIRC5
MYC TARGETS V2	57	1.05	0.353	AIMP2, HK2, NIP7, HSPD1, HSPE1, TFB2M, WDR43, TBRG4, GNL3, SLC19A1, MRTO4, RRP9
APOPTOSIS	157	-1.15	0.160	IER3, TAP1, RHOT2, GNA15, TIMP3, HMOX1, TIMP1, ADD1, PLCB2, SMAD7, PRF1, PLAT, IRF1, CD14, SLC20A1, DDIT3, GPX3, JUN, SOD2, CD2, TNFSF10, MCL1, BTG2, IL6, DAP, ENO2, MMP2, LEF1, TOP2A, PAK1, IGF2R, SATB1
APICAL JUNCTION	193	-1.21	0.099	ZYX, RAC2, MMP9, MSN, SYK, VWF, MYH10, VCAN, ACTN1, GNAI2, COL16A1, CAP1, CX3CL1, FLNC, NEXN, THBS3, DHX16, NLGN2, PKD1, GTF2F1, ICAM1, MMP2, INPPL1, RRAS, ITGA10, VAV2, ACTA1
IL2 STAT5 SIGNALING	189	-1.24	0.095	CD83, COL6A1, EOMES, COCH, GALM, TNFRSF1B, SLC2A3, PENK, PTGER2, GABARAPL1, CXCL10, FGL2, MXD1, SPP1, CAPG, GSTO1, ALCAM, IL10RA, TNFSF10, P2RX4, CD48, SELL, CST7, NFIL3, SOCS1, PDCD2L, FLT3LG, IGF2R, CKAP4, SERPINC1
INTERFERON ALPHA RESPONSE	91	-1.27	0.101	IRF7, TAP1, EPSTI1, IRF9, PROCR, TDRD7, CXCL10, IFI30, IRF1, CD47, TMEM140, NMI, PARP12, GBP2, GBP4, SELL, RIPK2, STAT2, PARP14, TRAFD1, ELF1, GMPR, OGFR, SLC25A28, RTP4
KRAS SIGNALING UP	193	-1.32	0.044	CFB, LCP1, F13A1, ITGB2, GPNMB, TRIB2, MAFB, IL7R, MMP9, ANGPTL4, MAP3K1, TNFRSF1B, PPBP, LY96, CXCL10, LAPTM5, SEMA3B, SPP1, PPP1R15A, DOCK2, TMEM176B, IL10RA, CTSS, HSD11B1, ITGBL1, SLPI, TMEM158, KCNN4, ANO1, TNFAIP3
COAGULATION	136	-1.34	0.056	C2, CFB, CTSB, TIMP3, TIMP1, F10, MMP9, THBS1, VWF, CD9, OLR1, C1QA, PLEK, COMP, CFI, PROS1, CTSH, LRP1, APOC1, MMP7, CSRP1, MMP14, MMP2
P53 PATHWAY	189	-1.35	0.028	IER3, TAP1, CTSD, PROCR, HMOX1, XPC, NINJ1, DEF6, NUPR1, MXD1, IFI30, MAPKAPK3, FOS, VAMP8, CTSF, DDIT3, JUN, PPP1R15A, TOB1, DRAM1, ST14, S100A4, BTG2, ERCC5, SOCS1, EPHA2, RRAD, HDAC3, TRAFD1, PRKAB1, RAD9A
EPITHELIAL MESENCHYMAL TRANSITION	195	-1.35	0.033	COL4A2, TIMP3, TIMP1, BASP1, MSX1, SDC4, THBS1, TNC, VCAN, COMP, COL16A1, COL12A1, SPP1, CAPG, PCOLCE, IGFBP4, ITGA5, ABI3BP, DPYSL3, LRP1, JUN, CALU, EMP3, SERPINH1, GLIPR1, THBS2, IL6, MMP14, ENO2, CRLF1, FLNA, TNFAIP3, SCG2
UV RESPONSE UP	151	-1.46	0.016	HLA-F, TAP1, RXRB, HMOX1, MSX1, JUNB, LYN, FOS, IRF1, GPX3, FOSB, SOD2, PDLIM3, MGAT1, FMO1, BTG2, CDC34, IL6, MMP14, ICAM1, DLG4, ENO2, DNAJB1, PSMC3, STARD3, RRAD, GAL, PPP1R2
COMPLEMENT	194	-1.52	0.004	IRF7, CCL5, C2, CFB, CTSB, CTSD, COL4A2, TIMP1, F10, WAS, GZMK, GZMA, GZMB, SPOCK2, PLAT, LYN, OLR1, GMFB, C1QA, C1QC, PLEK, PLA2G7, LIPA, GNAI2, ITGAM, FCN1, IRF1, EHD1, CBLB, CTSH, LRP1, APOC1, KYNU, SH2B3, CTSS, S100A9, CSRP1, CD55, JAK2, GATA3, IL6, SIRT6, RAF1, RHOG, MMP14, BRPF3, PPP4C, SERPINE1, PIK3CG, USP15, TNFAIP3, LTA4H, XPNPEP1, DOCK4, SERPINC1, ACTN2
INTERFERON GAMMA RESPONSE	194	-1.56	0.003	HLA-A, CCL5, HLA-B, TAP1, CFB, HLA-DRB1, HLA-DQA1, EPSTI1, PFKP, IRF9, ARID5B, GZMA, TDRD7, PTPN1, IDO1, MYD88, CXCL9, CXCL10, FGL2, SSPN, IFI30, VAMP8, MT2A, CCL2, IRF1, PSMB10, NMI, PARP12, SOD2, FPR1, IL10RA, TNFSF10, JAK2, RIPK1, IL6, ICAM1, SOCS1, RIPK2, RAPGEF6, TNFAIP2, TNFAIP3, TRAFD1, LATS2, OGFR, SLC25A28, RTP4, HLA-DQA2
IL6 JAK STAT3 SIGNALING	87	-1.65	0.004	IRF9, HMOX1, CD9, TNFRSF1A, LTBR, PTPN1, MYD88, TNFRSF1B, IL13RA1, CCR1, CXCL9, CXCL10, IRF1, CD14, JUN, IL17RA, IL6, SOCS1
TNFA SIGNALING VIA NFKB	196	-1.86	1.62E-05	CCL5, IER3, TAP1, CD83, KLF9, TRIP10, SDC4, IL7R, EGR2, NINJ1, SPSB1, JUNB, CEBPD, TNC, SLC2A3, OLR1, KLF2, CXCL10, NFKBIE, PLEK, MXD1, FOS, CCL2, IRF1, EHD1, EGR1, ZFP36, EIF1, SGK1, BCL3, RELB, FOSB, JUN, PPP1R15A, DUSP1, SOD2, DRAM1, MCL1, BTG2, IL6, NFIL3, ICAM1, IER2, RIPK2, SMAD3, TNFAIP2, TNFAIP3, PANX1, FUT4, TNFSF9, CLCF1

INFLAMMATORY RESPONSE	197	-1.98	1.33E-06	IRF7, CCL5, GNA15, TIMP1, IL7R, SEMA4D, LDLR, TNFRSF1B, LYN, PTGER2, OLR1, CXCL9, CXCL10, GPC3, MXD1, AQP9, STAB1, CCL2, CCL22, CX3CL1, IRF1, SLC11A2, PROK2, CCR7, ITGA5, CD14, AXL, MARCO, C5AR1, EMP3, NMI, FPR1, IL10RA, TNFSF10, P2RX7, P2RX4, CD48, SELL, RGS1, BTG2, CD55, IL6, RAF1, RHOG, MMP14, ICAM1, TLR1, ADORA2B, RIPK2, BEST1, FFAR2, SERPINE1, IL18RAP, HRH1, TNFSF9, CCL17, RTP4, SLC28A2
ALLOGRAFT REJECTION	195	-2.02	2.64E-07	RPS9, ELANE, HLA-A, HLA-E, CCL5, TAP1, HLA-DMB, C2, HLA-DRA, HLA-DQA1, HLA-DOA, IGSF6, CCL19, ITGB2, TIMP1, WAS, EIF3D, MMP9, GZMA, GZMB, PRF1, LYN, CCR1, CXCL9, SPI1, BCAT1, STAB1, CAPG, CCL2, CCL13, CCL22, CSK, PSMB10, BCL3, HCLS1, ITK, GBP2, CD2, CTSS, CD7, CD247, IL6, ICAM1, SOCS1, ITGAL, FLNA, HLA-DOB, CD4, TRAF2, HLA-DQA2

**Omega-6**				
**Pathway**	**Size**	**NES**	**AdjP**	**Leading Edge Genes**
MYOGENESIS	197	2.40	2.50E-09	MYH11, APP, PRNP, DMD, CACNA1H, SMTN, MYOM1, TPM2, COL4A2, COL6A2, SVIL, MYO1C, CLU, SOD3, AEBP1, FST, COL15A1, IGFBP3, VIPR1, CD36, APLNR, PPP1R3C, SORBS1, SCD, PC, GABARAPL2, MYLK, MYL4, COL1A1, MRAS, ACSL1, HSPB2, IGF1, CASQ2, COL3A1, GAA, COL6A3, SIRT2, IGFBP7, SH2B1, SLC6A8, PFKM, ITGA7, BIN1, HRC, SCHIP1, ADCY9, REEP1, DES, MAPRE3, ACTC1, CAMK2B, SPDEF, MYF6, ATP2A1, HBEGF, MAPK12, DMPK, PTP4A3, AGRN, KIFC3, KLF5, HDAC5, SYNGR2
ADIPOGENESIS	185	2.38	2.50E-09	CHCHD10, SLC25A1, DGAT1, ADIPOR2, PFKFB3, LPL, PPARG, CIDEA, CMBL, C3, ACO2, SLC5A6, LIFR, COL15A1, COX7B, STOM, YWHAG, CD36, DECR1, SORBS1, AK2, ANGPT1, ADCY6, ME1, IMMT, RTN3, VEGFB, COQ3, GPAM, LAMA4, PRDX3, DLD, IDH3A, ACLY, CDKN2C, CS, LIPE, ECHS1, APOE, MYLK, UCP2, MGLL, ACADM, DLAT, HIBCH, ALDH2, MCCC1, ADIPOQ, ACOX1, IDH1, ECH1, RREB1, TALDO1, UBQLN1, PREB, ATL2, G3BP2, SPARCL1, RETSAT, TKT, PTCD3, ITGA7, CD302, FZD4, LTC4S, AGPAT3, STAT5A, PEX14, PPP1R15B, CHUK
EPITHELIAL MESENCHYMAL TRANSITION	195	2.17	1.83E-08	POSTN, SAT1, TPM2, COL4A2, COL6A2, TGM2, BASP1, CDH6, CXCL12, GAS1, COL5A3, ECM2, FBLN1, IGFBP3, PMEPA1, TNC, NID2, MGP, VEGFA, ACTA2, BGN, COL16A1, TPM1, COL1A2, CTHRC1, COL5A1, IGFBP4, GJA1, MYLK, CALD1, SERPINH1, COL1A1, VEGFC, LUM, VCAM1, PLOD2, ITGAV, COL3A1, COL5A2, THY1, NTM, IGFBP2, SERPINE2, BMP1, LAMA3, FBLN5, CDH11, GPX7, SLIT3, FAP, PRRX1, ELN, FSTL1
OXIDATIVE PHOSPHORYLATION	179	2.14	8.82E-08	NDUFA6, SUCLA2, NQO2, PDHA1, RHOT2, NDUFV2, CYCS, ACO2, NNT, ACADVL, COX7B, UQCRC2, PDHX, LDHB, SLC25A20, DECR1, UQCRFS1, ALDH6A1, MRPS15, IMMT, MRPL35, PDHB, VDAC1, PRDX3, DLD, UQCRH, IDH3A, OAT, CS, ECHS1, ACADM, ETFDH, DLAT, SDHD, MTX2, IDH1, NDUFA3, ECH1, NDUFS1, CYB5R3, OGDH, LRPPRC, TIMM9, UQCRB, DLST, RETSAT, COX15, RHOT1, MRPL11, NDUFS8, ATP6V0E1, SLC25A4, SLC25A3, NDUFB3, MTRF1, SDHA, ISCA1, ABCB7, HSPA9, COX4I1, MRPS12, SUPV3L1, UQCRC1, IDH3B, NDUFS6, POLR2F, ALAS1, ACADSB, ACAA1, HTRA2, NDUFA1, ATP6V1G1, GRPEL1, HADHB, MRPS30
APICAL SURFACE	43	2.06	4.38E-04	APP, ADIPOR2, GAS1, LYN, CX3CL1, MAL, DCBLD2, THY1, B4GALT1, IL2RB, AFAP1L2, RTN4RL1, NTNG1, PCSK9, TMEM8B
FATTY ACID METABOLISM	153	2.03	6.15E-06	SUCLA2, ADIPOR2, BPHL, PDHA1, CIDEA, INMT, ACO2, XIST, ACADVL, CD36, CA2, DECR1, ELOVL5, ACOT2, HSD17B11, ME1, ADH1C, GPD1, PDHB, DLD, SERINC1, ECHS1, MGLL, ACADM, ETFDH, ACSL1, HIBCH, ERP29, EHHADH, ACOX1, IDH1, FASN, CD1D, HSD17B7, ALDH9A1, ECH1, IDI1, TP53INP2, AUH, DLST, RETSAT, AOC3, D2HGDH, LTC4S, ACSS1, APEX1, SDHA, HMGCS1, NBN, AADAT, GAD2, IDH3B, ACAA1, HADHB
HEDGEHOG SIGNALING	35	2.01	4.38E-04	VLDLR, CNTFR, AMOT, VEGFA, HEY1, HEY2, THY1, NRP1, PML, CRMP1, NF1, TLE3

UV RESPONSE DN	133	1.97	2.63E-05	TJP1, VLDLR, DLC1, ICA1, PPARG, NR1D2, RBPMS, SMAD7, KCNMA1, MAP1B, COL1A2, MAP2K5, MT1E, ADD3, FHL2, GJA1, DBP, MGLL, PTGFR, ATP2C1, COL1A1, INSIG1, COL3A1, COL5A2, IGFBP5, IRS1, ATP2B4, NFIB, ATXN1, NRP1, PIK3CD, KIT, FBLN5, ERBB2, FYN, NR3C1, MET, PDGFRB, MTA1, INPP4B, SCHIP1, YTHDC1, AKT3, RXRA, DLG1, MIOS, PEX14, TGFBR2
INTERFERON GAMMA RESPONSE	195	1.94	7.28E-06	HLA-A, CCL5, PSMB8, PSMB9, CFB, HLA-DQA1, C1R, IFITM3, IRF9, ISG15, LAP3, ARID5B, GZMA, XAF1, PIM1, PNPT1, CXCL10, FGL2, MVP, BST2, IFIT3, IFIT1, ISOC1, NAMPT, CD74, IFI44L, IFI44, IFIH1, SOD2, FPR1, VCAM1, CASP1, IL10RA, TNFSF10, LGALS3BP, CFH, IFNAR2, RIPK1, IL2RB, EIF2AK2, PELI1, HIF1A, IFIT2, VAMP5, PML, TNFAIP2, PDE4B, CASP4, TRAFD1, STAT4, FCGR1A, ADAR, SP110, LATS2, RNF31, ST8SIA4, TRIM25, OASL, RNF213, KLRK1, ITGB7, GPR18, CIITA, ZBP1, PTGS2, HLA-DQA2, IRF4, ZNFX1
APICAL JUNCTION	193	1.73	4.38E-04	TJP1, FSCN1, GAMT, CDH6, VCL, MDK, GNAI1, COL16A1, ACTG2, CX3CL1, MAP4K2, CADM2, VCAM1, INSIG1, MPZL2, THY1, ICAM2, JAM3, IRS1, MPZL1, LAMB3, B4GALT1, PKD1, BMP1, PLCG1, LAMA3, GTF2F1, DSC3, TRO, CDH11, TIAL1, SHC1, CADM3, CD34, CLDN14, NRXN2, VASP, BAIAP2, CTNND1, TAOK2, NF1, TMEM8B
COMPLEMENT	194	1.61	0.004	CCL5, NOTCH4, PSMB9, CFB, USP16, SERPINA1, COL4A2, F10, MAFF, APOBEC3F, APOBEC3G, C3, CLU, LAP3, GZMK, GZMA, GZMB, SPOCK2, PLAT, LYN, GNG2, LTF, C1QA, LAMP2, CD36, CA2, TFPI2, FCN1, ME1, KCNIP2, DYRK2, GP9, CTSC, GCA, DUSP6, CASP1, S100A9, CFH, CSRP1, PDGFB, F8, FYN, CASP4, CD46, GRB2, S100A12, LCK, PCSK9
INFLAMMATORY RESPONSE	197	1.60	0.004	CCL5, SLC7A2, IL7R, SEMA4D, TNFSF15, LYN, SLC4A4, CXCL10, BST2, MXD1, AQP9, APLNR, CSF3R, CX3CL1, PROK2, NAMPT, CCR7, DCBLD2, ATP2C1, FPR1, ATP2A2, TNFSF10, CD48, SELL, BTG2, IL2RB, PTGER4, PDPN, HIF1A, FFAR2, IL18RAP, PDE4B, KCNMB2, NMUR1, GABBR1, HPN, P2RY2, TLR3, NPFFR2, LCK, HBEGF, NOD2, IL1B, ICOSLG
ALLOGRAFT REJECTION	195	1.58	0.004	HLA-A, HLA-E, LTB, CCL5, HLA-DRA, HLA-DQA1, HLA-DOA, MAP4K1, CCL19, RPL9, SRGN, GZMA, GZMB, PRF1, LYN, F2R, CD8A, AKT1, CD74, ITK, CD2, CD3D, THY1, ETS1, CD1D, CD247, IL10, DEGS1, TAP2, IFNAR2, HDAC9, IL2RB, TLR6, HIF1A, ITGAL, IL18RAP, CD96, IL18, STAT4, SOCS5, KLRD1, ST8SIA4, CD79A, ABCE1, IL27RA, CD8B, ZAP70, TLR3, CRTAM, CCR2, TPD52, LCK, IL13, HLA-DQA2, IRF4, CCR5, TGFB2, IL1B, CD3G, ICOSLG
BILE ACID METABOLISM	111	1.54	0.014	AR, AQP9, HSD17B11, ISOC1, CYP46A1, LIPE, PEX11A, ACSL1, ABCA9, IDH1, PXMP2, ALDH9A1, IDI1, ATXN1, AMACR, PFKM, RETSAT, SLC29A1, RXRA, GSTK1, ABCA8, ABCA5, SLCO1A2, SULT2B1, LCK, ABCD3
KRAS SIGNALING UP	193	1.52	0.008	PSMB8, CFB, MAP4K1, CSF2RA, TLR8, F13A1, SPRY2, TSPAN7, TSPAN13, CIDEA, TRIB2, IL7R, IGFBP3, MAP3K1, PLAT, CLEC4A, PPBP, CXCL10, ENG, PLVAP, CA2, RBP4, YRDC, SERPINA3, DCBLD2, GYPC, CPE, DUSP6, MMD, TMEM100, MPZL2, ETS1, CFH, IL33, HDAC9, NRP1, SLPI, FBXO4, LAT2, ATG10, ABCB1, SPARCL1, PRDM1, CXCR4, CD37, PTBP2, CBL, PRRX1, IGF2, PTCD2, F2RL1, GNG11, ADAM8, RABGAP1L, SATB1, HOXD11, ACE, HBEGF, PTGS2, NAP1L2, SCG5, SDCCAG8, IL1B
PEROXISOME	101	1.51	0.022	DHRS3, MVP, SEMA3C, ELOVL5, PABPC1, SIAH1, HSD17B11, ISOC1, PEX11A, SOD2, ACSL1, EHHADH, ACOX1, IDH1, ALDH9A1, CLN8, ECH1, IDI1, ATXN1, DLG4, ABCB1, RETSAT, ERCC1, SLC25A4, GSTK1, PEX14, SULT2B1, HSD11B2, ACAA1, SCGB1A1, ABCD3
ANGIOGENESIS	36	1.49	0.083	APP, POSTN, LPL, VEGFA, JAG2, SLCO2A1, LUM, ITGAV, COL3A1, COL5A2, FSTL1, PGLYRP1
XENOBIOTIC METABOLISM	195	1.48	0.014	CFB, BPHL, F10, ACO2, FBLN1, TNFRSF1A, PTGR1, CD36, CA2, AQP9, ELOVL5, RBP4, CDO1, ADH1C, PTGDS, ENPEP, CSAD, IGFBP4, APOE, CYFIP2, ETFDH, IGF1, ATP2A2, ALDH2, ACOX1, IDH1, ALDH9A1, GSTT2, ECH1, NDRG2, RETSAT, ABHD6,

				CYP2S1, ESR1, PYCR1, MCCC2, PPARD, TMBIM6, BCAR1, GCNT2, ARG2
IL2 STAT5 SIGNALING	189	1.48	0.010	LTB, PRNP, SERPINB6, IFITM3, COL6A1, TGM2, MAFF, MYO1C, EOMES, NOP2, DHRS3, HOPX, SLC2A3, CD44, PIM1, CXCL10, FGL2, PRKCH, CA2, MXD1, HK2, LRIG1, ADAM19, ITGA6, IL10RA, ITGAV, TNFSF10, S100A1, CD48, SELL, IL10, CYFIP1, CST7, NRP1, IL2RB, AMACR, LCLAT1, P4HA1, PTH1R, ABCB1, CDC42SE2, SNX14, SPRY4, PLAGL1, SOCS2, CKAP4, CD79B, BMPR2, IL1R2, SMPDL3A, FAM126B, RABGAP1L, CAPN3, CCNE1, IL13, IL3RA, IRF4, IL18R1, SNX9, SYNGR2
WNT BETA CATENIN SIGNALING	42	1.47	0.083	NOTCH4, CTNNB1, HEY1, GNAI1, HEY2, JAG2, NCOR2, CSNK1E, SKP2, LEF1, PPARD
HYPOXIA	188	1.42	0.034	SLC25A1, VLDLR, PFKFB3, IRS2, TGM2, MAFF, SLC2A5, IGFBP3, MAP3K1, DDIT4, PCK1, SLC2A3, VEGFA, UGP2, STBD1, WSB1, PPP1R3C, BGN, ALDOC, HK2, COL5A1, TGFB3, NDST1, CSRP2, ETS1, SELENBP1, GAA, PDGFB, P4HA1, TPI1, PLAC8, NR3C1, CXCR4, PKP1, GCNT2, B3GALT6, PKLR, PGF
INTERFERON ALPHA RESPONSE	91	1.41	0.065	HLA-C, PSMB8, PSMB9, IRF9, ISG15, TRIM5, LAP3, PNPT1, CXCL10, BST2, IFIT3, CD74, IFI44L, IFIH1, CASP1, LGALS3BP, SELL, EIF2AK2, IFIT2, TRAFD1, ADAR, SP110, TRIM25, OASL, MOV10
MITOTIC SPINDLE	194	1.37	0.047	NET1, RHOT2, NEDD9, MID1IP1, FSCN1, MYH9, RAPGEF5, SOS1, VCL, SPTBN1, RABGAP1, WASF2, ARHGAP4, PKD2, FLNB, MARCKS, NUMA1, AKAP13, PALLD, SORBS2, ATG4B, YWHAE, MID1, KIF3B, FARP1, HDAC6, TUBGCP6, LRPPRC, RASA1, MAP1S, RAPGEF6, SAC3D1, MAP3K11, ARAP3, KLC1, BIN1, ARHGEF12, CDC42BPA, TBCD, ABR, CTTN, CDC42, ARFGEF1, EPB41, ARHGEF3, PLEKHG2, NCK2, CYTH2, TUBD1, PXN, DLG1, ARHGEF2, ARHGDIA, RASAL2, KIF15, BCAR1, CEP250, CLIP2, STK38L, LATS1, ARHGEF7, STAU1, SMC3, NEK2, ABI1, TAOK2, NF1, SASS6, KIF4A, ALMS1, PREX1, SHROOM1, PAFAH1B1
MTORC1 SIGNALING	190	1.34	0.081	ACACA, VLDLR, ADIPOR2, PSMG1, DDIT4, SLC2A3, NUPR1, FGL2, ELOVL5, CORO1A, MAP2K3, HK2, SCD, ME1, IMMT, SLA, ADD3, TM7SF2, NAMPT, ACLY, SERPINH1, SYTL2, CTSC, PLOD2, INSIG1, ATP2A2, HMBS, HSPD1, SLC9A3R1, IDH1, IGFBP5, BTG2, IDI1, RPA1, P4HA1, TPI1, NFYC, CXCR4, EEF1E1, HMGCS1, ARPC5L, HSPA9, PSMD12, PSMB5, POLR3G
ESTROGEN RESPONSE EARLY	188	1.23	0.100	NRIP1, ITPK1, SLC24A3, DLC1, SLC7A2, SVIL, TGM2, CXCL12, AR, DHRS3, CD44, ELOVL5, RHOBTB3, FLNB, ADD3, LRIG1, IGFBP4, FHL2, GJA1, CCND1, PEX11A, PODXL, PRSS23, PDLIM3, AKAP1, OLFML3, SLC9A3R1, NCOR2, FASN, B4GALT1, MED24, RPS6KA2, ADCY9, FAM102A, REEP1, BCL11B, NADSYN1, TMPRSS3, P2RY2, SULT2B1, PDZK1, LAD1, HR, ADCY1, KRT15, TFF3, KRT19, CELSR2
NOTCH SIGNALING	32	1.23	0.260	NOTCH3, HEYL, CCND1, DTX1, FBXW11, PPARD, ARRB1, TCF7L2
IL6 JAK STAT3 SIGNALING	87	1.17	0.260	LTB, CSF2RA, CNTFR, IRF9, TNFRSF1A, PIM1, A2M, CXCL10, CD36, CSF3R, PDGFC, IL17RA, IL10RB, CBL, IL1R2, GRB2, DNTT, IL3RA, INHBE, IL1B, IL18R1
GLYCOLYSIS	194	1.13	0.281	VLDLR, CACNA1H, IRS2, PFKFB1, IGFBP3, DDIT4, CD44, VEGFA, UGP2, GFPT1, PGAM1, HK2, COL5A1, ALDH7A1, ME1, GLCE, DLD, PC, AGL, PLOD2, HAX1, IDH1, RPE, ALDH9A1, B4GALT1, TALDO1, EGLN3, P4HA1, TPI1, TPST1, ARPP19, STMN1, PGLS, GMPPB, P4HA2, NOL3, MED24, MET, CXCR4, CTH, HS2ST1, IDUA, LDHC, HS6ST2, PAM, CHPF, CASP6, DEPDC1, LHX9, NDUFV3, B3GALT6, FBP2, LCT, B3GAT1, TGFA, GAPDHS, COG2, AGRN, GYS1, ALDOB, KIF2A, CHPF2, TFF3
SPERMATOGENESIS	128	1.08	0.373	PSMG1, YBX2, ARL4A, DMC1, SLC2A5, PRKAR2A, IP6K1, SLC12A2, ELOVL3, JAM3, TALDO1, PIAS2, GMCL1, HSPA2, CCT6B, NEFH, PARP2, TLE4, LDHC, CFTR, EZH2, PEBP1, NF2, ACRBP, PHF7, ZC3H14, CNIH2, NPHP1, DPEP3, ACE, TSSK2, AKAP4, PRM2, GFI1, NEK2, ALOX15, PGK2, GAPDHS, HSPA4L, TULP2, SCG5, IFT88, CAMK4, TEKT2, BRAF, HTR5A, ADCYAP1, ART3, NOS1
APOPTOSIS	158	1.05	0.417	APP, RHOT2, SAT1, NEDD9, IFITM3, ADD1, CLU, SMAD7, DFFA, PRF1, PLAT, CTNNB1, CD44, F2R, GPX1, DNM1L, BGN, CCND1,

				SOD2, LUM, CASP1, GSTM1, CD2, TNFSF10, FEZ1, BTG2, BMF, RETSAT, LEF1, ERBB2, PDGFRB, CTH, CASP4, IL18, CASP2, SATB1, CASP6, MADD, TGFB2, IL1B
PI3K AKT MTOR SIGNALING	103	1.02	0.480	ACACA, RPS6KA1, PLCB1, MKNK2, TNFRSF1A, CDKN1B, PRKAR2A, ITPR2, MAPK10, MAP2K3, SLA, AKT1, PPP2R1B, MAPK1, RIPK1, PLCG1, YWHAB, MKNK1, CXCR4, MAPK9, RAC1, EIF4E, UBE2D3, IL4, GRB2, RPTOR, FGF17, MAPK8, LCK, PRKAA2, MAP2K6, CAMK4, PAK4, AKT1S1
PROTEIN SECRETION	95	0.97	0.539	RAB9A, ICA1, LAMP2, DNM1L, TMED10, CAV2, TSPAN8, CLCN3, CTSC, SEC22B, VAMP4, ERGIC3, RAB5A, ABCA1, AP2S1, COPB1, GBF1, ARFIP1, STX16, TPD52, COG2, RAB14
KRAS SIGNALING DN	187	0.96	0.594	CNTFR, GAMT, FGFR3, SLC16A7, KCNMB1, PTGFR, IFI44L, GPRC5C, IGFBP2, BTG2, YPEL1, MFSD6, PKP1, IDUA, NRIP2, PLAG1, UGT2B17, LYPD3, INSL5, CAPN9, MSH5, PRODH, NUDT11, ACTC1, AMBN, DLK2, GTF3C5, HTR1B, SNCB, MEFV, EPHA5, CD40LG, COL2A1, TCL1A, TAS2R4, FGF16, HSD11B2, SERPINA10, ALOX12B, MYO15A, CPEB3, TG, PNMT, KLK7, TCF7L1, KRT15, IFNG, TGFB2, SCGB1A1, ATP4A, CELSR2, NOS1
PANCREAS BETA CELLS	40	0.85	0.733	LMO2, AKT3, VDR, NEUROG3, NKX2-2, PKLR, SPCS1, NEUROD1
DNA REPAIR	138	-0.75	0.952	XPC, STX3, SF3A3, HCLS1, GTF2H1, TYMS, HPRT1, COX17, ERCC2, SEC61A1, POLA1, TAF10, POM121, DUT, ELL, POLA2, REV3L, POLD4, NT5C, RFC4, GUK1, DAD1, POLR2J, TARBP2, GTF2B, RBX1, VPS37D, AAAS, RNMT, POLB, DDB2, PRIM1, POLR3GL, AK3, POLR3C, RAD52, CLP1, POLR2G, CMPK2, POLR2D
MYC TARGETS V2	57	-0.98	0.531	RCL1, AIMP2, LAS1L, MYC, NIP7, MCM5, MAP3K6, SRM, MRTO4, PLK4, PHB, MYBBP1A, PES1
UNFOLDED PROTEIN RESPONSE	106	-0.98	0.539	GEMIN4, HSPA5, CCL2, BAG3, HSP90B1, KHSRP, CALR, EXOSC2, ATP6V0D1, CKS1B, PARN, WFS1, CEBPG, YIF1A, DNAJB9
MYC TARGETS V1	185	-1.04	0.426	RANBP1, PRPS2, DEK, AIMP2, PSMA2, PABPC4, MYC, CDC20, PSMD8, LSM2, TYMS, MCM5, GSPT1, EXOSC7, RPS5, RRM1, FAM120A, SRM, TXNL4A, SNRPD2, PSMD7, PHB, YWHAQ, POLE3, PCBP1, SERBP1, PSMB3, MRPL9, LSM7, CCT4, CCNA2, HNRNPC
COAGULATION	136	-1.06	0.403	CTSB, MSRB2, MMP9, THBS1, CD9, GSN, FBN1, PLEK, PROS1, APOC1, MMP7, SERPINE1, CAPN2
TGF BETA SIGNALING	54	-1.14	0.337	CDKN1C, ID2, RAB31, THBS1, PPP1R15A, IFNGR2, BMP2, HDAC1, SERPINE1, ACVR1
P53 PATHWAY	189	-1.15	0.240	HRAS, IER3, TAP1, CTSD, PMM1, TAX1BP3, KLF4, IFI30, ZFP36L1, MAPKAPK3, VAMP8, NOTCH1, SLC3A2, INHBB, GM2A, FAM162A, PERP, PPP1R15A, ZBTB16, ST14, S100A4, BMP2, CGRRF1, CCND3, IRAK1, NDRG1, HDAC3, SEC61A1, PRKAB1, S100A10, DCXR, MXD4, TNFSF9, POM121, RAB40C, SLC35D1, DDB2, SP1, PLK3, ABHD4, CDKN2AIP, TCHH
HEME METABOLISM	187	-1.17	0.217	E2F2, CTSB, RCL1, HBZ, HBD, BLVRA, ACP5, EPB42, ELL2, AHSP, MPP1, SNCA, SLC11A2, BLVRB, UROS, TFRC, LRP10, FOXJ2, CCND3, HEBP1, ATP6V0A1, SIDT2, PIGQ, XPO7, SLC2A1, CPOX, VEZF1, RAP1GAP, UBAC1, ARL2BP, TMEM9B, KLF1, GMPS, SLC30A10, FBXO34, MARK3
ANDROGEN RESPONSE	96	-1.19	0.240	FKBP5, IQGAP2, SRF, ELL2, SLC38A2, FADS1, SGK1, DHCR24, ALDH1A3, UAP1, CCND3, ZMIZ1, CDK6, UBE2J1, NDRG1, HPGD, HMGCR, DNAJB9, MAP7, CENPN
CHOLESTEROL HOMEOSTASIS	72	-1.21	0.240	TNFRSF12A, CD9, LDLR, LGALS3, ALCAM, PLAUR, ATF5, S100A11, FDPS, ACTG1, NSDHL, ANXA5, GUSB, HMGCR
UV RESPONSE UP	151	-1.24	0.139	CDKN1C, TAP1, RXRB, SQSTM1, MSX1, PPT1, IRF1, GPX3, FOSB, TFRC, BMP2, CCND3, PSMC3, STIP1, CYB5B, GAL, RPN1, GLS, DNAJA1, CEBPG, NPTX2, TGFBRAP1, POLE3, FKBP4, ATP6V1F, CXCL2, TCHH
REACTIVE OXYGEN SPECIES PATHWAY	44	-1.24	0.251	MSRA, TXN, GPX3, TXNRD1, OXSR1, PRDX1, PRDX6, SOD1, HMOX2
TNFA SIGNALING VIA NFKB	196	-1.28	0.080	IER3, TAP1, SQSTM1, EDN1, CD83, ID2, KLF9, EGR2, SPSB1, LDLR, KLF4, NFKBIE, PLEK, CCL2, MYC, IRF1, EHD1, ZFP36, SGK1, PLAUR, BCL3, FOSB, KYNU, PPP1R15A, DUSP1, TIPARP,

				IFNGR2, BMP2, NFIL3, RIPK2, NFKB1, TRIB1, SERPINE1, DUSP5, PHLDA1, HES1, TNFSF9, SERPINB2, CXCL2
G2M CHECKPOINT	180	-1.38	0.038	E2F2, SLC7A1, CKS2, SFPQ, RAD21, MT2A, SYNCRIP, MYC, CDC20, BCL3, MCM6, PTTG1, ATF5, MCM5, GSPT1, NUSAP1, AURKA, KIF22, CCNB2, E2F4, TOP2A, RBM14, PRC1, SAP30, DR1, CKS1B, BARD1, E2F1, SUV39H1, PRMT5, PLK4, HMMR, CCNF, CDKN3, PBK, KIF11, TROAP, CENPE, GINS2, CENPF, DBF4, TPX2, ESPL1, CCNA2, CHAF1A, BUB1
ESTROGEN RESPONSE LATE	194	-1.42	0.026	FDFT1, FOXC1, ID2, RAB31, IMPA2, SNX10, UGDH, CD9, KLF4, FKBP5, SLC27A2, SLC2A8, PAPSS2, CYP26B1, CDC20, CXCL14, ZFP36, SERPINA5, BLVRB, SGK1, PERP, HSPB8, ST14, GLA, MAPK13, MYOF, TOP2A, GAL, DCXR, RNASEH2A, MICB, NXT1, FKBP4, XRCC3, GINS2, ARL3, KIF20A, SCNN1A
E2F TARGETS	183	-1.55	0.005	RANBP1, ASF1B, DEK, CKS2, PRPS1, RAD21, SYNCRIP, MYC, CDC20, MCM6, PTTG1, TFRC, MCM5, PHF5A, IPO7, DNMT1, GSPT1, AURKA, KIF22, CCNB2, GINS3, TOP2A, PAN2, CKS1B, BARD1, MELK, SUV39H1, RNASEH2A, MCM4, DUT, DSCC1, CBX5, PLK4, HMMR, TRIP13, RRM2, CDKN3, DCLRE1B, RAD51AP1, CENPE, NUP107, BUB1B, DLGAP5, TK1, UBE2T, BRCA2, SPC24, CDCA3, ESPL1, NUP205, ZW10
